# Disclosure is not documentation: an open science framework for documenting generative AI use in scholarly research and publication workflows

**DOI:** 10.1186/s41073-026-00245-8

**Published:** 2026-08-15

**Authors:** Jan Christopher Cwik

**Affiliations:** Faculty of Health Care, Hochschule Niederrhein, Reinarzstraße 49, 47805 Krefeld, Germany

**Keywords:** Generative AI, Large language models, AI disclosure, AI-use documentation, Open science, Research integrity, Peer review, Publication ethics, Scholarly publishing, Clinical psychology

## Abstract

**Background:**

Generative AI is increasingly used in scholarly research, writing, and publication workflows. Many journal and publisher policies ask authors to disclose relevant AI use, but disclosure alone rarely clarifies how AI-assisted work was performed, what information was entered, how outputs were evaluated, or how human responsibility was maintained. This creates a gap between AI-use disclosure as a publication requirement and AI-use documentation as an open science practice.

**Methods:**

This article develops a conceptual and practical framework for documenting generative AI use in scholarly workflows. The framework was informed by exploratory, non-systematic source and policy mapping, AI-assisted exploratory evidence mapping, manual review of selected recent literature, and development of accompanying Open Science Framework materials. These steps were used to identify recurring documentation expectations and unresolved policy gaps and to translate them into practical documentation domains, with particular attention to task specificity, proportionality, role-specific documentation, privacy-sensitive transparency, and clinically sensitive contexts.

**Results:**

The framework distinguishes disclosure from documentation and proposes documentation fields for minimal and extended AI use. Minimal documentation is intended for low-risk uses such as limited language polishing, whereas extended documentation is recommended when AI supports literature synthesis, coding, analysis, interpretation, manuscript drafting, peer-review-related work, clinical material, or research procedures. The framework is accompanied by reusable OSF materials, including documentation templates, prompt-log structures, declaration examples, checklists, clinical redaction guidance, and source-tracking materials.

**Conclusions:**

AI-use disclosure communicates that AI was used; documentation makes the AI-assisted workflow traceable, inspectable, and accountable. A task-specific, proportionate, role-specific, and privacy-sensitive documentation approach can support responsible AI use while protecting confidential, patient-related, peer-review-related, and methodologically sensitive information.

The accompanying bilingual materials are openly available on OSF: 10.17605/OSF.IO/A439J.

**Supplementary Information:**

The online version contains supplementary material available at 10.1186/s41073-026-00245-8.

## Background

In recent years, artificial intelligence (AI) has become increasingly embedded in scholarly work and scientific publishing, not as a single technology but as a heterogeneous set of tools used across research, writing, and publication workflows. These tools range from language support and manuscript preparation to literature discovery, coding, evidence mapping, systematic-review support, and specialized clinical or technical applications [1, 2]. Because such uses differ substantially in their function, input requirements, and potential influence on scholarly outputs, AI use cannot be adequately addressed through generic disclosure alone.

This development is not limited to manuscript preparation. Bibliometric and experimental evidence suggests that AI is becoming a consequential component of scientific work more broadly, influencing research trajectories, writing workflows, and the production of scholarly outputs [1, 3]. AI-assisted tools are also increasingly discussed across health-related fields, where they may support writing, literature synthesis, data-related workflows, and complex medical or clinical tasks, but require validation, domain expertise, and sustained human oversight [4]. AI use in scholarly work should therefore not be conceptualized merely as language polishing, but as part of a broader transformation of research workflows, knowledge production, and publication practices.

The potential benefits of these tools are accompanied by important risks. Early work in medical education already framed generative AI as a dual-use technology: potentially useful for writing assistance, simulation-based learning, and personalized educational support, but also associated with concerns about academic integrity, inaccurate or hallucinated information, bias, and overdependence [5]. In scientific writing, AI-generated text may appear fluent and authoritative while containing inaccurate mechanisms, fabricated references, or misleading identifiers [6–9]. These findings make human verification and transparent documentation central requirements of responsible AI use.

Generative AI may also affect the style, originality, and diversity of scholarly communication. Although AI tools can improve clarity, coherence, and organization, their widespread and insufficiently documented use may contribute to homogenized scientific writing, reduced authorial voice, and diminished transparency regarding the origin of scholarly ideas and formulations [10, 11]. This issue is not only technical or ethical, but also cultural, because generative AI changes how writing, authorship, literacy, and professional competence are understood and valued in academic contexts [12].

These concerns are particularly salient in peer review and editorial workflows, where AI use may involve confidential manuscripts, intellectual property, reviewer selection, editorial judgment, and human oversight. Recent studies on prompt injection, AI-assisted reviewer identification, and editors-in-chief’s perspectives show that generic AI-use disclosure is insufficient in these contexts and that role-specific documentation is needed [13–16].

The need for documentation becomes particularly pronounced in clinically sensitive fields such as clinical psychology, psychotherapy, psychiatry, and mental health research. In such contexts, AI-assisted workflows may involve patient-related information, clinical narratives, diagnostic content, emotionally evocative material, or vulnerable populations. Documentation must therefore balance transparency with confidentiality, data protection, output verification, and human oversight [17–19].

Recent publication policies and guidance documents increasingly recognize AI-related risks, but the reviewed literature suggests that these policies remain heterogeneous and unevenly operationalized. Many guidelines reject AI authorship, request or require disclosure of relevant AI use, emphasize human accountability, and protect peer-review confidentiality; however, they often provide limited operational guidance on how the underlying AI-assisted workflow should be documented [20–22]. Against this background, the present article argues that AI-use disclosure is necessary but insufficient, and proposes an open-science-oriented documentation framework to make AI-assisted scholarly workflows more transparent, traceable, interpretable, and accountable (see Table 1).

**Table 1:** Overview of the article

Section	Main function
Background	Explains why disclosure alone is insufficient for AI-assisted scholarly workflows
Methods	Describes the conceptual framework-development process and exploratory source/policy mapping
Results	Presents the documentation principles, documentation tiers, OSF materials, and clinical safeguards
Discussion	Interprets the framework’s implications for research integrity, authors, reviewers, journals, institutions, and sensitive-data disciplines
Limitations	Clarifies the exploratory evidence base, lack of empirical evaluation, confidentiality constraints, and need for future adaptation

### Generative AI in scientific writing

AI-assisted writing should not be treated as a single, homogeneous practice. In scholarly writing, digital and AI-supported tools operate along a continuum. Some provide low-level language support, whereas others can restructure arguments, generate or revise manuscript passages, synthesize sources, support coding, or contribute to literature-related work. The transparency relevance of AI use, therefore, depends less on the name of the tool than on its function in the workflow: what the system was asked to do, what material was entered, how its output was evaluated, and whether it influenced the final argument, interpretation, or presentation of findings.

Within this broader ecosystem, large language models have become particularly relevant because they can intervene at multiple stages of manuscript preparation. They may support early idea development, help structure a manuscript, revise or translate text, summarize source material, assist with literature-related tasks, generate code, or help prepare responses to reviewers. Prior work on AI chatbots in scientific writing has emphasized both this practical usefulness and the accompanying concerns about transparency, copyright, misinformation, and the responsibilities of authors, reviewers, editors, and journals [23]. Scoping and synthesis work similarly suggest that AI-chatbot applications extend beyond language editing to research design, programming, translation, content generation, and scientific knowledge synthesis, although earlier findings should be interpreted in light of the rapid development of new systems [24, 25].

The benefits of such tools are not merely cosmetic. Experimental evidence suggests that generative AI can materially alter professional writing workflows by reducing the time required for writing tasks and improving the quality of externally evaluated output [26]. From an open science perspective, this makes AI use relevant to the provenance of scholarly texts. If a tool changes how a text is drafted, revised, improved, or synthesized, readers and reviewers have a legitimate interest in understanding how it contributed to the final manuscript. At the same time, AI assistance may affect not only individual writing quality but also the diversity and originality of outputs across authors. Evidence that AI assistance can improve individual creative performance while reducing collective output diversity suggests that, when applied cautiously to scholarly communication, insufficiently documented AI use may shape the framing and originality of scientific contributions, not only their linguistic clarity [11].

These issues are especially relevant for researchers writing in a non-native language. AI-assisted translation, academic language editing, and revision support may reduce barriers to participation in English-language scholarly communication and make publication opportunities more accessible [27]. However, the same tools also raise questions about authorial voice, originality, critical thinking, and responsibility. Generative AI should therefore not be framed simply as a productivity tool, but as a technology that may influence the rhetorical, conceptual, and epistemic production of scientific texts. This broader cultural shift is reflected in discussions of generative AI writing as a disruption of established assumptions about academic writing, authorship, literacy, and professional competence [12].

Taken together, this literature supports a functional rather than purely tool-based understanding of AI use in scientific writing and scholarly publishing. Minor language polishing or translation support should not be treated in the same way as literature synthesis, coding assistance, manuscript drafting, or substantive content generation, because these uses differ in how strongly they may shape the scholarly workflow and the final manuscript. AI-use documentation should therefore be proportionate to the role of AI: limited assistance may require only brief reporting, whereas uses that affect arguments, interpretations, methods, outputs, or editorial interactions require more detailed documentation of task design, prompting, output handling, verification, and human oversight.

### Current disclosure requirements

As generative AI has become more common in scholarly writing and publishing, many journals, publishers, and editorial organizations have introduced or updated policies on its use. These policies indicate a shift away from relying solely on post-submission detection of AI-generated text. Instead, much of the reviewed editorial guidance emphasizes transparency, human accountability, and responsible disclosure. Comparative work on editorial policies suggests that many major journals and editorial organizations reject AI authorship, ask authors to disclose relevant AI use, and place responsibility for accuracy, originality, and integrity on human authors [28]. The same work also cautions that AI-detection tools have accuracy limitations and may introduce bias, making them unsuitable as the sole basis for editorial or misconduct-related decisions.

Despite partial convergence around disclosure and human accountability, current policies remain heterogeneous and only partly operationalized. Many reviewed policies indicate that substantive use of AI should be disclosed and that human authors remain accountable for the final work. Some reviewed policies also address specific high-risk contexts, such as the use of AI in peer review or in relation to AI-generated and AI-manipulated images [29–34]. Some publishers distinguish routine copyediting from more substantive AI assistance and ask authors to describe the purpose, relevance, or verification of their use of AI.

However, the reviewed policies differed considerably in the amount of process-level information they requested or required, particularly regarding model versions, prompts, input materials, output handling, manual corrections, and privacy safeguards [29–34].

This creates a gap between disclosure as a policy requirement and documentation as an open science practice. The reviewed policies often clarify when AI use should be disclosed, but they are less consistent in explaining how researchers should document the workflow behind that disclosure. A statement that an AI tool was used, that its output was checked, or that the authors remain responsible for the manuscript may satisfy a journal requirement, but it does not necessarily make the AI-assisted process interpretable. Readers, reviewers, and editors may still be unable to determine what the tool contributed, what information was entered, how outputs were selected or rejected, what was manually corrected, or how privacy-sensitive decisions were handled.

Empirical audit evidence suggests that this gap was already visible in the early phase of publisher policy development. In a cross-sectional audit of 162 scientific, technical, and medical publishers, only about one-third had publicly available policies on authors’ use of AI chatbots as of late 2023. Among publishers with policies, disclosure and human accountability were common themes, and AI authorship was not permitted; however, policies varied in their treatment of proofreading, image generation, non-methodological writing, formal research methods, and the specificity of required reporting [22]. These findings provide an empirical baseline for the present argument: the audited publisher policies recognized several AI-related risks, but varied in how far they translated these concerns into operationally usable documentation fields.

Some early publications illustrate that more process-oriented documentation is feasible. For example, AI-assisted evidence-mapping work has reported the AI-supported task, the input source, the human verification step, and the availability of prompts or workflow materials [35]. Such examples show that authors can move beyond a generic declaration without making documentation unmanageable. At the same time, they also reveal the need for more consistent standards regarding interaction records, versioning, prompt iterations, output selection, and manual corrections. Overall, the reviewed policy landscape and early examples of process-oriented reporting suggest a movement toward transparency, while also indicating fragmentation and the absence of a standardized framework for documenting AI-assisted scholarly workflows (see Table 2).

**Table 2:** How the proposed framework extends existing AI-use guidance

Guidance type	Typical focus	Common limitation	Extension provided by the proposed framework
Journal and publisher policies	Disclosure, authorship, accountability, confidentiality	Often limited process-level detail	Adds structured documentation fields for task, input, prompts, output handling, verification, and safeguards
Editorial and peer-review guidance	Confidentiality, reviewer responsibility, human oversight	Often unclear how AI-assisted review workflows should be recorded	Adds role-specific documentation for authors, reviewers, editors, and research teams
Institutional guidance	Responsible use, data protection, training, tool access	Often broad and not tailored to manuscript or publication workflows	Provides adaptable templates for project-level and manuscript-level documentation
Reporting guidance for AI-related studies	Reporting of AI systems used as research objects or interventions	Not always designed for AI use in writing, synthesis, review, or publication workflows	Extends documentation to scholarly workflow tasks beyond AI as the study object
Open-science materials	Transparency, reuse, provenance, accountability	Rarely specific to generative-AI-assisted workflows	Adds prompt logs, redaction logic, workflow records, declaration templates, and OSF-based documentation structures

Representative sources informing the comparison include empirical and comparative analyses of journal and publisher policies and editorial guidance [20–22, 28–34], as well as literature addressing AI use in peer review and scholarly workflows [13–16, 36, 37]. The table provides a conceptual synthesis and should not be interpreted as a systematic frequency analysis of policy requirements

### Disclosure is not documentation

The central argument of the present article is that disclosure is necessary but not sufficient. A disclosure statement tells readers that AI was used; documentation explains how AI entered the scholarly workflow, what role it played, how its outputs were evaluated, and how human responsibility was maintained (see Table 3). This distinction matters because the integrity implications of AI use depend on function and context. Minor language correction, translation support, literature synthesis, manuscript drafting, coding assistance, peer-review-related use, and AI-generated research materials do not raise the same transparency and accountability questions. Throughout the manuscript, “AI-use” is used for disclosure and documentation constructs, whereas “AI-assisted” refers to workflows, tasks, outputs, or procedures involving AI support.

**Table 3:** Operational distinction between AI-use disclosure and AI-use documentation

AI-use disclosure refers to a brief statement that generative AI was used, usually identifying the tool and the general purpose of use. It is often sufficient when AI use is limited to spelling correction, grammar checking, or minor language polishing and did not affect the scholarly substance of the work
AI-use documentation refers to a structured record of how AI entered the scholarly workflow, including the task, tool or model, input type, prompt or prompt category, output handling, human verification, privacy safeguards, and relevance for the final manuscript. It is warranted when AI use affects literature synthesis, coding, analysis, interpretation, drafting, research materials, clinical content, or peer-review-related work
Restricted or redacted documentation refers to documentation that is retained privately, summarized, or redacted because full public disclosure would expose confidential, identifiable, proprietary, clinically sensitive, or peer-review-related material

From an open science perspective, AI-use documentation should therefore be understood as part of a broader transparency agenda. Open science is concerned not only with access to research outputs but also with the transparency of research practices, decisions, and contributions. Discussions of generative AI writing as a cultural disruption highlight why AI-use disclosure is more than a technical requirement: academic writing also signals expertise, originality, and professional competence [12]. Undisclosed AI assistance may obscure how a text was produced and how much intellectual, rhetorical, or interpretive work was performed by human authors. At the same time, AI use should not be treated as inherently suspicious. As AI-assisted writing becomes increasingly normalized, transparent documentation can help distinguish legitimate support from problematic uses such as undisclosed rewriting, source obfuscation, or plagiarism [38].

Empirical evidence further supports the distinction between retrospective detection and prospective documentation. Studies on plagiarism, AI-assisted rewriting, and AI-generated scientific essays suggest that post hoc provenance judgments remain uncertain: AI-rewritten or plagiarized text may evade available detection tools, while human-authored scientific text may be misclassified as AI-generated [38, 39]. These findings indicate that publication integrity cannot depend primarily on retrospective detection. Prospective documentation provides a more reliable basis for transparency because it records the process by which AI-assisted work was produced, reviewed, modified, or excluded.

Field-specific evidence points in the same direction. In eating-disorder research, generative AI has been used for tasks such as proofreading and coding support, yet only about one-third of users reported disclosing such use [40]. This has led to recommendations for clearer AI-use statements and submission-system prompts that ask authors to specify how they used AI. The issue is particularly relevant for mental health research, where AI-assisted workflows may involve sensitive data, stigma, diagnostic interpretation, lived-experience perspectives, culturally sensitive content, or vulnerable populations. In such contexts, disclosure can signal that AI was used, but documentation is needed to explain the role AI played, the safeguards applied, and how its outputs were assessed before they become part of the scholarly record.

The same distinction is important in peer review and editorial workflows [13, 14, 16, 36]. Reviewers’ and editors’ use of AI differs from authors’ use because it may involve unpublished manuscripts, confidential information, intellectual property, and evaluative judgments that require disciplinary expertise. Peer-review-specific guidance, therefore, supports cautious, transparent, and human-supervised AI use rather than generic disclosure alone. Recent studies further illustrate why more detailed documentation is needed: prompt injection can turn manuscripts into sites of strategic manipulation of AI-assisted review processes, while AI-assisted reviewer identification may produce different reviewer pools depending on prompt design workflows [13, 14]. Documentation should therefore clarify the role, purpose, safeguards, confidentiality constraints, and human oversight of AI use in review-related contexts.

Examples from psychological and health research illustrate how process-oriented documentation can be implemented when AI becomes part of the research procedure itself. A protocol for AI-integrated communication training reported the platform, model version, prompt-management procedures, transcript handling, monitoring steps, privacy safeguards, and Open Science Framework (OSF) registration [41]. Similarly, a study evaluating LLMs on ethnically adapted versions of the Reading the Mind in the Eyes Test documented model selection, access mode, administration period, repeated test sessions, prompt instructions, raw data, and OSF materials [42]. AI-generated personalized stimuli in psychological experiments provide another example, because such work requires transparency about participant-related inputs, stimulus generation, output evaluation, and safeguards for sensitive material [43]. Together, these examples show that AI-use documentation can move beyond naming the tool by making the research workflow more inspectable.

By contrast, some AI-use statements remain transparent but brief. For example, AI-use statements in digital mental health research may identify the tool and general purpose of use without documenting the underlying workflow in detail [44]. This is not necessarily inconsistent with existing journal requirements, but it illustrates the difference between disclosure and documentation: a declaration can meet a policy requirement while still leaving readers unable to assess how AI-assisted work was actually performed. Recent editorial guidance has begun to move closer to documentation-oriented standards by recommending that authors and reviewers describe relevant AI systems, their limitations, version, date of use, and, where appropriate, established reporting guidelines such as CONSORT-AI or STARD-AI [37]. However, existing recommendations still do not provide reusable templates for documenting prompts, output handling, manual corrections, versioning, and privacy-sensitive workflow decisions.

Together, these examples show that disclosure and documentation serve different functions. Disclosure communicates the presence of AI use; documentation makes the workflow inspectable. For open science, the latter is essential because it supports traceability, accountability, critical appraisal, and responsible reuse. AI-use documentation should therefore capture, where relevant, the tool or model used, the purpose and context of use, the nature of the inputs, the handling and verification of outputs, privacy safeguards, and the relevance of AI-assisted work for the final manuscript.

### Aim of the present article

The aim of the present article is to address the gap between AI-use disclosure as a publication requirement and AI-use documentation as an open science practice. Although many journals and publishers now request or require authors to disclose relevant generative-AI use, these requirements often provide limited operational guidance on how the underlying AI-assisted workflow should be documented.

To address this gap, the article proposes a structured, open-science-oriented framework for documenting AI use in scholarly research and publication workflows. The framework is designed to move beyond generic AI-use declarations by supporting task- and role-specific documentation that is proportionate to the function of AI use and sensitive to privacy and confidentiality constraints.

The framework is particularly attentive to clinical psychology, psychotherapy, and related mental health fields, where AI-assisted workflows may involve sensitive data, vulnerable populations, confidential materials, diagnostic interpretations, or emotionally evocative content. More broadly, it seeks to clarify how researchers can document AI-assisted workflows in ways that support author accountability, editorial evaluation, peer-review integrity, reproducibility where feasible, and the critical appraisal of the role of AI in the production of scholarly work.

### Operational objectives, intended users, and scope of application

The operational aim of the framework is not to introduce a new mandatory reporting standard, but to provide a modular documentation aid that can be adapted by authors, reviewers, editors, journals, research teams, publishers, institutions, and funders. Its practical purpose is to help users decide when a brief AI-use declaration is sufficient, when extended workflow documentation is warranted, and when documentation should be redacted or retained privately because sensitive, confidential, proprietary, or peer-review-related material is involved.

The framework is therefore intended as a baseline structure from which more targeted disciplinary, institutional, or journal-specific adaptations can be derived. It does not replace existing policies, ethics requirements, or legal obligations. Instead, it translates broad transparency and accountability expectations into documentation fields that can be incorporated into manuscript preparation, submission systems, reviewer guidance, institutional training, or OSF-based project documentation.

## Methods

### Aim and scope of the framework

This article develops a conceptual and practical framework for documenting the use of generative AI in scholarly research, writing, and publication workflows. The framework is intended to support transparent, proportionate, role-specific, and privacy- and confidentiality-sensitive documentation. It focuses on information that is usually not captured by brief AI-use declarations, such as the role of AI in the workflow, the nature of the input, the handling of outputs, the process of human verification, and the relevance of AI assistance for the final manuscript or research procedure.

The framework is grounded in an open science perspective. AI-use documentation is understood here not merely as a response to journal requirements, but as a way of making research practices and scholarly contributions more inspectable. Because generative AI can influence not only wording but also literature searching, coding, analysis, interpretation, argument development, and publication-related workflows, documenting its use can help readers, reviewers, and editors evaluate how AI-assisted work contributed to the published output.

The framework does not aim to replace journal, publisher, institutional, or funder policies. Rather, it is intended as a practical documentation aid and adaptable baseline framework that researchers can tailor to their disciplinary context, target journal, institutional requirements, data-sensitivity level, and confidentiality constraints. Because documentation expectations will necessarily differ across disciplines and publication contexts, the framework should be understood as a modular structure rather than a universal checklist to be applied identically in all cases.

### Source and policy mapping

The framework was informed by exploratory source and policy mapping of current guidance on the use of AI in scholarly research and publishing. The mapping considered journal and publisher policies, editorial statements, research-integrity recommendations, reporting guidance, policy analyses, and institutional materials addressing generative AI and large language models in research, writing, peer review, and publication workflows. Its purpose was to identify recurring expectations, restrictions, and unresolved issues relevant to AI-use disclosure and documentation, rather than to produce a comprehensive or systematic review of all AI-related guidance or publication policies. This focus was motivated by prior evidence that publication policies on generative AI remain heterogeneous and vary in the level of operational detail they provide to authors [21, 22].

The mapping examined not only whether AI use should be disclosed, but also what kinds of information the reviewed policies and guidance documents asked authors to provide. Particular attention was given to whether guidance addressed the role of AI in the workflow, the type of input, human verification, confidentiality, the placement of AI-use statements, and the distinction between minor language assistance and more substantive AI involvement.

### AI-assisted exploratory evidence mapping

To support the development of the documentation framework, an AI-assisted exploratory evidence mapping was conducted using Elicit on April 24, 2026. Because the Elicit user interface did not display a specific software or model version, the tool name, subscription plan, date of use, review workflow, and exported report file were documented in the supplementary materials.

The guiding review question was: What information do current policies require or recommend that researchers disclose or document when using AI tools in scientific studies, manuscript preparation, data analysis, literature searches, peer review, or publication processes?

The Elicit-supported search was used to identify relevant source types, policy domains, and possible reporting requirements. Data extraction was conducted for *N* = 253 sources retained after the Elicit-supported screening stage. Due to processing limitations in the tool, the final AI-generated synthesis report included the *N* = 200 sources with the highest Elicit screening scores. Accordingly, the documentation domains proposed below should be interpreted as a synthesis of recurring expectations and practical documentation needs identified through an exploratory process, rather than as evidence of uniform policy consensus.

The extracted information was organized around recurring issues in AI-use policy and documentation, including disclosure expectations, authorship and accountability, methodological reporting, confidentiality, peer review, data-related uses, and restricted or prohibited applications. All substantive policy claims retained in the final manuscript were manually reviewed by the author and verified against the cited primary policy or guidance sources.

The AI-assisted mapping was therefore treated as an exploratory and supporting step rather than a fully systematic review. The mapping should not be interpreted as an exhaustive account of all AI policies. It may have missed relevant institutional policies, journal-specific webpages, non-English sources, or recently updated publisher guidance. Researchers using the framework should therefore continue to check the current policies of their target journals, publishers, institutions, and funders (see Table 4).

**Table 4:** Link between recurring source/policy expectations and documentation domains

**Documentation domain**	**Recurring expectation or concern identified in the mapping**	**Added value of the framework**
Tool/model identification	Reviewed policies often ask authors to identify AI tools used	Adds provider, model/version where available, access mode, and date or period of use
Purpose/task	Reviewed policies often request a general statement of AI use	Specifies the concrete workflow task, such as editing, synthesis, coding, analysis, drafting, or peer-review-related support
Input type and sensitivity	Confidentiality, privacy, and data-protection concerns recur across policy sources	Documents whether inputs involved public, confidential, proprietary, participant-related, clinical, or peer-review-related material
Prompt documentation	Prompting is rarely operationalized in publication policies	Allows exact prompts, redacted prompts, or prompt categories depending on risk and confidentiality
Output handling	Human responsibility and accountability are common policy expectations	Documents whether AI outputs were accepted, modified, rejected, or incorporated
Human verification	Accuracy, originality, and citation reliability are recurring concerns	Requires documentation of fact-checking, citation checks, expert review, or other verification steps
Privacy safeguards	Reviewed policies often warn against inappropriate data input but provide limited workflow detail	Adds redaction, anonymization, access control, data minimization, and private audit records
Manuscript relevance	Reviewed policies often distinguish minor editing from substantive AI involvement	Clarifies whether AI influenced arguments, methods, interpretation, conclusions, or presentation

The documentation domains summarize recurring expectations and concerns identified through the exploratory source and policy mapping. Representative sources include policy analyses and publisher guidance [20–22, 28–34], supplemented by literature on prompt-sensitive and process-oriented AI-assisted scholarly workflows [13, 14, 37, 41, 42]. The table is intended as a conceptual mapping rather than a quantitative analysis of policy frequencies

### Development of documentation domains

The documentation domains were developed by translating recurring policy and source-based expectations, together with open-science principles, into practical documentation elements. The aim was to create a framework that is detailed enough to support transparency, accountability, and editorial evaluation, but flexible enough to accommodate different levels of AI involvement, disciplinary contexts, and confidentiality requirements.

After the initial AI-assisted mapping step, selected recent articles [13–15, 22, 36, 45, 46] were manually reviewed to contextualize the framework with respect to emerging issues such as AI-assisted peer review, prompt-injection risks, editorial governance, mental-health research, and examples of process-oriented AI-use documentation. These sources were used to refine the argument and documentation domains rather than as part of a systematic review.

The development process followed three principles. First, documentation should be task-specific, meaning that it should describe what AI was used for rather than merely naming the tool. Second, documentation should be proportionate to the role of AI in the workflow: minor language support requires less detail than AI use in literature synthesis, coding, analysis, interpretation, stimulus generation, clinical material, or peer-review-related work. Third, documentation should be privacy-sensitive so that transparency does not require disclosing confidential, identifiable, proprietary, or clinically sensitive material.

Based on the exploratory mapping, the framework was organized around domains that capture the tool or model used, the purpose and context of use, the nature of the input, prompt or prompt-category information, output handling, human verification, privacy safeguards, and the relevance of AI assistance for the final manuscript. Prompt-related documentation was retained as a core domain because prompt design can materially affect AI-assisted scholarly outputs, as shown in experimental work on AI-assisted reviewer identification [14].

### Development of OSF materials

The framework was translated into accompanying Open Science Framework materials to help researchers document AI use in a structured, privacy-sensitive manner. The materials distinguish between minimal documentation for low-risk or limited AI use and extended documentation for more substantive, sensitive, or methodologically consequential AI-assisted workflows.

The material package includes guidance documents, prompt-log templates, checklists, AI-use declaration templates, fictional and redacted examples for clinical psychology and psychotherapy, source- and policy-tracking materials, editing and versioning workflow templates, and a reusable example prompt for adapting the package. These materials are intended as adaptable resources rather than fixed reporting standards.

Minimal documentation is intended for limited uses, such as spelling and grammar correction or minor language polishing. Extended documentation is intended when AI supports tasks that may affect the scholarly substance of the work, such as manuscript drafting, literature synthesis, data analysis, coding, interpretation, image or stimulus generation, clinical material, or peer-review-related work.

The templates were designed to support documentation without encouraging inappropriate disclosure of sensitive information. They therefore allow authors to use redacted prompt descriptions, prompt categories, summaries of input types, and privacy notes. The materials emphasize that confidential peer-review content, unpublished third-party manuscripts, identifiable personal information, clinical case material, and sensitive participant data should not be entered into public AI tools or reproduced in public prompt logs.

### Adaptation to clinical psychology and psychotherapy

The framework was adapted with particular attention to clinical psychology, psychotherapy, psychiatry, and related mental-health fields because AI-assisted workflows in these areas may involve patient-related information, diagnostic content, therapy notes, case vignettes, qualitative interview material, stigma-sensitive content, emotionally evocative stimuli, or vulnerable populations. This adaptation reflects the broader concern that AI use in clinically sensitive contexts requires traceability, privacy protection, human oversight, and safeguards against harmful, biased, or stigmatizing outputs [18, 47–50].

For this reason, the clinical adaptation of the framework includes documentation on data sensitivity, input restrictions, redaction procedures, human oversight, scope limitations, bias-related checks, and limits on public disclosure. In clinically sensitive contexts, documentation should not be equated with unrestricted publication of raw prompts or AI interactions. Instead, it should make the AI-assisted workflow sufficiently inspectable while protecting confidential and sensitive information.

### Use of generative AI in developing the materials

Generative AI and AI-assisted writing tools were used supportively during manuscript preparation and revision. ChatGPT Pro was used on 30 April 2026 for language correction, readability improvement, and argumentative stringency/coherence checks of author-generated manuscript text. Grammarly was used for spelling, grammar, punctuation, style, readability, and language correction. Elicit was used on 24 April 2026 for AI-assisted exploratory evidence and policy mapping, as described above.

All AI-assisted outputs were reviewed, edited where necessary, and approved by the author. AI-assisted suggestions were not treated as authoritative sources. All substantive policy claims retained in the final manuscript were manually reviewed by the author and verified against the cited primary policy or guidance sources. No personal clinical data, identifiable case material, confidential peer-review content, unpublished third-party manuscripts, or sensitive participant data were entered into public AI tools.

A reusable example prompt for creating and adapting the documentation materials is included in the OSF repository. The prompt specifies intended outputs, quality criteria, safety requirements, and the need for human review and verification. The author takes full responsibility for the content, interpretation, structure, and final wording of the manuscript and accompanying materials. Detailed project-specific documentation of these AI-assisted workflows, including task-specific use records, prompt documentation, output handling, human verification, and privacy safeguards, is provided in Additional file 1.

## Results

The framework development resulted in six main outputs: a specification of the documentation gap, a set of core documentation principles, a distinction between minimal and extended documentation, a package of accompanying OSF materials, clinical safeguards and redaction principles, and an open-access structure for reuse and adaptation.

### The documentation gap

The exploratory source and policy mapping suggested a persistent documentation gap in current AI-use reporting. Many reporting practices focus on whether AI was used, but provide less information about the role AI played in the scholarly workflow. A generic statement such as “AI was used during manuscript preparation” may therefore obscure important differences between minor language support and AI assistance that affects literature synthesis, coding, analysis, interpretation, peer-review-related tasks, research materials, or other substantive components of scholarly work.

The proposed framework addresses this gap by translating broad expectations for AI-use disclosure into practical documentation elements. These elements clarify, where relevant, the task performed, the tool or model used, the nature and sensitivity of the input, prompt, or prompt-category information, output handling, human verification, privacy safeguards, and the relevance of AI assistance to the final manuscript.

### Core principles of the framework

The framework is organized around seven core principles: task specificity, proportionality, human accountability, verification, provenance, privacy-sensitive transparency, and role specificity. Together, these principles translate broad AI-use transparency expectations into practical documentation requirements while keeping documentation feasible for routine scholarly use. Table 5 summarizes the practical implication of each principle.

**Table 5:** Core principles of the AI-use documentation framework and their practical documentation implications

Principle	Practical documentation implication
Task specificity	State what AI was used for, not only which tool was used
Proportionality	Match documentation depth to scholarly relevance and risk
Human accountability	Clarify that humans remain responsible for final content
Verification	Describe how outputs, claims, references, or code were checked
Provenance	Record tool/model, access mode, and date or period of use
Privacy-sensitive transparency	Use redaction or private records where full disclosure would be harmful
Role specificity	Distinguish author, reviewer, editor, publisher, and research-team uses because these roles involve different responsibilities, confidentiality constraints, and risks

These principles are operationalized in the minimal and extended documentation fields presented in Table 6

### Minimal and extended documentation fields

The framework distinguishes between minimal and extended documentation. Minimal documentation is intended for limited, low-risk AI use, such as spelling correction, grammar support, or minor language polishing. It should identify the tool, the general purpose of use, the affected stage of the manuscript or workflow, and the human verification process. It should also confirm that no confidential or sensitive material was entered into inappropriate AI systems.

Extended documentation is intended for AI use that may affect the scholarly substance, methodological process, evidentiary basis, or interpretive claims of the work. This includes, for example, AI-supported drafting, translation, literature synthesis, coding, analysis, interpretation, image or stimulus generation, clinical content, or publication-related decision-making. In such cases, documentation should provide more detail about the tool or model, access mode, date or period of use, prompting procedure, input type, output handling, verification, modifications, privacy safeguards, and the relevance of AI-assisted work for the final manuscript.

Table 6 summarizes the proposed documentation fields and illustrates how documentation depth can be adapted to the scholarly relevance, sensitivity, and risk level of AI-assisted work.

**Table 6:** Minimal and extended AI-use documentation fields for scholarly workflows

Documentation field	Minimal documentation	Extended documentation
Tool identification	Tool name and general category	Tool name, provider, model, version if available, access mode
Date or period of use	Approximate date or period	Exact date or documented period of use
Purpose of use	General purpose, e.g., language editing	Specific task, e.g., literature synthesis, coding, analysis, drafting
Input type	General statement that no sensitive data were used, if applicable	Description of input source, sensitivity level, and any redactions
Prompt documentation	Not required for minor language polishing	Exact prompt, redacted prompt, or prompt category
Output use	General statement that outputs were reviewed	Description of what was accepted, modified, rejected, or incorporated
Human verification	Confirmation of human review	Description of verification steps, fact-checking, citation checks, or expert review
Modifications	Not usually required	Description of substantive manual corrections or revisions
Privacy safeguards	Confirmation of no inappropriate data input	Redaction, anonymization, access control, or data minimization procedures
Manuscript relevance	General manuscript stage affected	Whether AI influenced arguments, conclusions, methods, interpretation, or presentation
Disclosure location	AI-use statement, acknowledgments, or methods	Methods, supplement, OSF record, declaration, or submission-system field

Minimal documentation is intended for limited, low-risk AI use, such as spelling, grammar, or minor language polishing. Extended documentation is intended for more substantive AI-assisted workflows, such as drafting, literature synthesis, coding, data analysis, interpretation, stimulus generation, or work involving sensitive data. The distinction is intended to make documentation proportionate to the scholarly relevance and potential risks of AI use

This tiered structure keeps documentation proportionate and is intended to reduce unnecessary burden. Minor spelling, grammar, and readability support may require only minimal documentation, whereas more detailed records are reserved for AI use that affects scholarly substance, methodology, interpretation, sensitive material, or publication-related decision-making.

### Overview of the eight OSF components

The framework is accompanied by eight OSF components designed to support practical implementation. These materials translate the documentation principles into reusable resources for authors, reviewers, editors, and research teams. They are intended to help users decide when a brief AI-use declaration is sufficient, when more detailed workflow documentation is needed, and when documentation should be redacted or kept partly private because sensitive information is involved. Table 7 provides an overview of the OSF materials and their intended purposes.

**Table 7:** Overview of the eight OSF components supporting implementation of the AI-use documentation framework

OSF document	Purpose
1. Documentation manual	Explains the rationale, principles, documentation fields, prompt-log logic, privacy-sensitive documentation, and disclosure/storage logic
2. Prompt-log templates	Provides minimum and detailed prompt-log templates, prompt-chain documentation, output-use and human-review templates, code/statistics templates, literature/claim checking, declaration preparation, and version logs
3. Checklists	Provides practical checklists for project setup, tool/model documentation, prompt-log completeness, data protection, human review, literature/source checking, code/statistics, AI-use declarations, OSF upload, revisions, and non-public materials
4. AI-use declaration templates	Provides adaptable wording for manuscripts, methods sections, supplements, OSF descriptions, cover letters, reviewer responses, and different levels of AI involvement
5. Clinical example documentations	Provides fictional and redacted worked examples for clinical psychology and psychotherapy, including low-, medium-, and high-risk AI-use scenarios
6. Sources and guidelines	Provides a curated source base, policy-orientation tables, target-journal policy tracking, source appraisal, update routines, and citation/deposit notes
7. Editing and versioning	Provides workflows for file naming, version control, change logs, editorial decisions, review cycles, redaction logs, release notes, OSF upload checks, and public/internal/redacted versions
8. Example prompt for creating materials	Provides a reusable meta-prompt for generating or adapting documents 01–07, including quality criteria, safety requirements, and transparency safeguards

The OSF components translate the proposed framework into reusable documentation materials for authors, reviewers, editors, and research teams. They include templates, checklists, worked examples, source-tracking tools, versioning workflows, and redaction guidance. The materials are intended as adaptable support resources and do not replace journal, publisher, institutional, funder, or ethics board requirements

Together, the OSF components support cross-disciplinary adaptation. A manuscript requiring only limited language polishing may require minimal documentation, whereas a study using AI for literature synthesis, coding, clinical material, image generation, or peer-review-related tasks may require an extended workflow template. The materials also distinguish between private documentation, supplementary documentation, and public documentation. Some records may be retained for auditability or editorial review, whereas others may be suitable for public sharing. In sensitive contexts, prompt logs may need to be redacted or summarized rather than published verbatim.

### Clinical safeguards and redaction principles

Clinical psychology, psychotherapy, psychiatry, and related mental-health fields require additional safeguards because AI-assisted workflows may involve patient-related information, diagnostic content, therapy-related material, qualitative interviews, case vignettes, stigma-related information, emotionally evocative stimuli, or vulnerable populations. In such contexts, documentation should not be equated with unrestricted public disclosure of raw prompts, outputs, or AI interactions.

The framework therefore includes clinical safeguards and redaction principles. These principles are intended to make AI-assisted workflows sufficiently inspectable while protecting sensitive information. Researchers should avoid entering identifiable personal data, confidential therapy material, unpublished third-party manuscripts, peer-review content, or sensitive participant information into public AI tools. Where AI use involves clinical or otherwise sensitive material, documentation should describe the type of information involved without exposing protected content. Prompt logs may therefore need to be redacted, summarized, or documented through prompt categories rather than exact prompts.

Clinical AI-use documentation should clarify the function of AI use, the sensitivity of the input material, the safeguards applied, and the role of human review. If AI is used to summarize qualitative interview material, generate clinical vignettes, assist with coding, or create emotionally evocative stimuli, documentation should explain how input data were protected, how outputs were evaluated, whether content was excluded or modified, and what human expertise was involved. Recent psychiatric AI-governance work similarly emphasizes the importance of transparency, accountability, evaluation, compliance, and harm prevention in clinically sensitive AI contexts [50].

The guiding principle is that AI-use documentation in clinical contexts should be transparent enough to support accountability but protective enough to avoid exposing patient- or participant-related, or confidential material.

### Open access to the materials

The documentation framework and accompanying materials are openly available through the Open Science Framework. Open access to the materials allows researchers, reviewers, editors, and institutions to adapt the templates to their disciplinary, ethical, and publication requirements.

The OSF repository includes the documentation manual, minimal and extended templates, AI-use declaration examples, checklists, clinical redaction guidance, worked examples, and a source and policy tracker. The materials are designed as reusable and updateable resources rather than fixed reporting standards. Because AI tools, publisher policies, and legal requirements are changing rapidly, the OSF materials should be treated as living resources rather than static standards. They can be updated when AI tools, publisher policies, institutional rules, legal requirements, or disciplinary expectations change. The fictional and redacted worked examples included in the OSF materials are intended to function as test cases for applying and adapting the framework without exposing real clinical, participant-related, or peer-review material.

Researchers using the framework should continue to consult the current policies of their target journals, publishers, institutions, funders, and ethics boards. The framework does not replace these requirements. Instead, it provides a structured way to document AI use more consistently and transparently, particularly when existing policies require disclosure but do not specify how the underlying AI-assisted workflow should be recorded.

The repository link, version information, and persistent identifier are reported in the data availability statement.

## Discussion

This article proposed a task-specific, proportionate, role-specific, and privacy-sensitive framework for documenting the use of generative AI in scholarly research and publication workflows. Building on the distinction developed in the Background and operationalized in the Results, the Discussion focuses on implications for research integrity, authors, reviewers, journals, institutions, and disciplines handling sensitive data.

### Main contribution

The main contribution of this article is the translation of broad AI-use transparency expectations into practical documentation domains and reusable OSF materials. The framework extends generic AI-use declarations by specifying what should be recorded about task, tool/model, input type, prompting, output handling, human verification, privacy safeguards, and manuscript relevance. Its contribution is therefore practical rather than prescriptive: it offers a modular structure that authors, reviewers, editors, journals, and institutions can adapt to different levels of AI involvement and sensitivity.

### Implications for research integrity

The framework has direct implications for research integrity because publication integrity cannot depend primarily on retrospective detection. AI-related threats extend beyond legitimate but poorly documented assistance. Case evidence of false authorship illustrates how generative AI can produce apparently scholarly articles that are fraudulently attributed to real researchers or used to inflate publication records [45]. Such cases point to the need for provenance mechanisms that connect AI-use reporting with author accountability, identity verification, manuscript provenance, and peer-review transparency.

At the same time, empirical work on AI-generated or AI-rewritten scientific text shows that post hoc provenance judgments remain uncertain. Studies on plagiarism, AI-assisted rewriting, and AI-generated scientific essays suggest that AI-rewritten or plagiarized text may evade detection tools, while human-authored scientific text may be misclassified as AI-generated [38, 39]. These findings should not be interpreted as a definitive assessment of all current detection systems, but they illustrate a key problem: retrospective detection is uncertain, context-dependent, and vulnerable to both false negatives and false positives. Prospective documentation can reduce this ambiguity by clarifying how AI tools were used, what outputs were incorporated, and how human authors verified the final manuscript.

### Implications for authors, reviewers, journals, and institutions

The proposed framework has different implications for authors, reviewers, journals, publishers, and institutions. For authors, it emphasizes responsibility and verification. Authors should not only declare AI use, but also document the role AI played in the workflow, the type of input provided, how outputs were handled, and how human verification was performed. This is especially important when AI tools are used for literature synthesis, coding, analysis, interpretation, citation support, or drafting of substantive manuscript sections. Authors remain responsible for the accuracy, originality, integrity, and ethical acceptability of all AI-assisted content.

For reviewers and editors, the framework highlights confidentiality and role-specific restrictions. Peer review is a particularly sensitive context because unpublished manuscripts contain confidential information and intellectual property, and because evaluative judgments require disciplinary expertise. Recent studies suggest that AI use in peer review should not be governed only by prohibition or generic disclosure. Prompt-injection risks show that AI-assisted review infrastructures may be vulnerable to strategic manipulation when the use of AI in evaluation processes is opaque [13]. Conversely, randomized evidence on AI-assisted reviewer identification suggests that explicit prompting may help create more diverse reviewer pools [14]. These examples indicate that the relevant governance question is not simply whether AI was used, but how, where, with what prompts or criteria, and under which human oversight. Documentation should clarify whether AI was used only to improve the language of reviewer feedback or also to summarize, evaluate, or interpret manuscript content. The reviewed publisher policies and guidance documents often caution against uploading submitted manuscripts, figures, tables, or peer-review reports into public generative AI systems because this may compromise confidentiality, proprietary rights, and data privacy [29–33].

For journals and publishers, the framework suggests that submission systems should move beyond binary checkboxes on AI use. More informative submission forms could ask authors to specify the purpose of AI use, the part of the workflow affected, the tool or model used, the verification process, and whether sensitive or confidential material was involved. Such structured fields would make AI-use declarations more comparable across manuscripts and reduce ambiguity for editors and reviewers. Survey evidence from biomedical editors-in-chief reinforces this need: many editors report limited direct use of AI chatbots in editing, policy uncertainty, training needs, resource constraints, and ethical concerns related to bias, transparency, accountability, and human judgment [15]. A tiered documentation approach may therefore be more feasible than a single maximal reporting requirement, with minimal records for low-risk uses and extended or restricted documentation for substantive, sensitive, or editorially consequential AI-assisted workflows.

For institutions, the framework highlights the need for AI literacy, training, and infrastructure. Researchers need guidance not only on whether AI use is permitted, but also on how to document it responsibly. Institutions can support this by providing approved tools, training on data protection and prompt documentation, templates for AI-use reporting, and guidance on enterprise or privacy-preserving AI systems. Scholarly communication guides and library resources can support this work, although concrete requirements should still be checked against current policies from journals, publishers, institutions, and funders [51].

### Relevance for disciplines handling sensitive data

The need for process-oriented AI-use documentation is especially pronounced in disciplines handling sensitive data, with clinical psychology and psychotherapy serving as important examples. Clinical psychology, psychotherapy, psychiatry, qualitative research, and related fields may involve patient-related information, diagnostic descriptions, therapy material, case vignettes, qualitative interviews, trauma-related content, stigma-sensitive material, emotionally evocative stimuli, or vulnerable populations. In such contexts, documentation must balance transparency with confidentiality and data protection.

The relevant question is therefore not only whether AI was used, but what function it served, what type of information was entered, how outputs were evaluated, what safeguards were applied, and how human responsibility was maintained. Documentation should capture input sensitivity, redaction procedures, scope limitations, bias or harm checks, escalation or referral safeguards where relevant, and the boundaries of public disclosure. This allows AI-assisted workflows to be inspectable without requiring the public release of confidential, identifiable, or clinically sensitive material.

### Implementation recommendations

The framework can be implemented through three documentation tiers. Minimal documentation is appropriate when AI use is limited to low-risk language support, such as spelling correction, grammar checking, or minor readability improvements. Extended documentation is needed when AI contributes to the scholarly substance of the work, for example, by supporting literature synthesis, coding, analysis, interpretation, manuscript drafting, citation-related work, image or stimulus generation, clinical material, or reviewer responses. Restricted or redacted documentation is appropriate when public disclosure of prompts, inputs, or outputs would expose confidential, identifiable, proprietary, peer-review-related, or clinically sensitive information.

Implementation should be gradual and context-sensitive. Journals could pilot structured AI-use fields in submission systems; institutions could integrate the templates into research-integrity training and data-protection guidance; research teams could use the OSF materials as internal project documentation; and reviewers or editors could use the checklist logic to assess whether an AI-use statement provides sufficient information. Recognition of the framework would therefore not depend on adoption by a single organization, but on iterative use, adaptation, and evaluation across scholarly communities.

Restricted or redacted documentation is particularly important for qualitative and reflexive research, where the methodological role of human interpretation may itself be central to validity. Recent qualitative-methods scholarship has argued that generative AI is methodologically incongruent with reflexive qualitative approaches such as reflexive thematic analysis because these approaches depend on subjective, positioned, and reflexive human meaning-making [46]. The present framework, therefore, does not assume that all AI use is appropriate in all methods. Rather, documentation should make visible when AI was not used for methodological reasons, when its use was limited to non-analytic support, or when an AI-assisted step requires explicit justification because it may affect interpretation, reflexivity, or epistemic responsibility.

To support implementation, journals and institutions could provide structured reporting fields rather than relying solely on free-text AI-use declarations. Such fields should help authors clarify the purpose of AI use, the type of input provided, whether sensitive or confidential material was involved, how outputs were checked, and whether AI assistance affected substantive arguments, methods, analyses, interpretations, or conclusions.

The accompanying OSF materials are intended to support this process through adaptable templates, prompt-log structures, AI-use declaration examples, clinical redaction guidance, author and reviewer checklists, and policy-tracking resources. They should be understood as adaptable resources rather than fixed reporting standards, given that AI tools and publisher policies are changing rapidly.

## Limitations

This article has several limitations. First, the framework is conceptual and practice-oriented. It was developed to support transparent AI-use documentation, but it has not yet been empirically evaluated for usability, adoption, burden, or impact on editorial decision-making. A further limitation is that the framework was developed by a single author rather than by a formal working group, consensus panel, editorial board, or professional society. This allowed rapid development of a practical open-science resource in response to a fast-moving problem, but it also limits the breadth of disciplinary, editorial, institutional, and stakeholder perspectives represented in the framework. Future work should therefore evaluate, revise, and extend the framework through multi-stakeholder consultation, pilot implementation, and discipline-specific adaptation. Future studies should examine whether researchers, reviewers, and editors find the proposed templates feasible, whether they improve transparency, and whether they can be integrated into journal submission systems without creating excessive administrative burden. Particular attention should be given to whether the tiered distinction between minimal, extended, and restricted documentation reduces unnecessary workload compared with one-size-fits-all reporting requirements.

Second, the source and policy mapping was exploratory rather than systematic. Although it was useful for identifying recurring policy domains and possible documentation expectations, it should not be interpreted as an exhaustive review of all AI-related journal, publisher, institutional, funder, ethics-board, or legal requirements. In addition, selected recent sources were added manually to contextualize emerging issues such as prompt injection, AI-assisted peer review, false authorship, editorial governance, and reflexive qualitative research. This manual contextualization strengthened the framework’s relevance but also reflects the author’s judgment rather than systematic coverage of the literature. Researchers using the framework should therefore continue to consult the current policies of their target journals, publishers, institutions, ethics boards, and funders.

Third, the evidence mapping was AI-assisted. Although all substantive policy claims retained in the final manuscript were manually reviewed by the author and verified against the cited primary policy or guidance sources, the use of AI-assisted search and synthesis tools may have influenced the visibility, prioritization, and organization of sources.

Fourth, the framework cannot resolve all problems of AI reproducibility. Generative AI systems are dynamic, and outputs may vary across model versions, interfaces, system prompts, settings, dates, and access modes. In some cases, researchers may not have access to full model-version information, temperature settings, system-level instructions, or provider-side changes. Documentation can make these constraints visible, but it cannot guarantee exact reproducibility of AI-generated outputs. For this reason, the framework should not be treated as a fixed standard. Its stable element is the documentation logic—task, input, output handling, verification, sensitivity, and relevance—rather than any specific tool, model, or policy snapshot. This makes the framework adaptable even when particular AI systems, publisher policies, and legal requirements change.

Fifth, there is a tension between transparency and confidentiality. Full public disclosure of prompts, inputs, or outputs may be inappropriate when AI use involves sensitive clinical material, personal data, unpublished manuscripts, proprietary information, peer-review content, or vulnerable populations. The framework therefore recommends privacy-sensitive documentation, including redacted prompts, prompt categories, summarized input descriptions, and private audit records where necessary. However, further work is needed to determine which level of documentation is appropriate in different legal, clinical, and disciplinary contexts.

Sixth, the framework focuses primarily on research, writing, and publication workflows. It does not provide a complete governance framework for clinical AI deployment, regulatory approval, patient-facing systems, or automated decision-making. Although the manuscript discusses clinical psychology and psychotherapy as sensitive fields, its primary aim is to improve documentation of AI use in scholarly work rather than to regulate clinical AI tools themselves.

Finally, the framework is not intended to stigmatize AI use or to imply that all AI assistance is problematic. On the contrary, the framework assumes that AI tools can be useful and legitimate when used responsibly. Its purpose is to make such use transparent, proportionate, verifiable, and accountable.

## Conclusion

Generative AI is becoming embedded in scholarly research, writing, and publishing, ranging from minor language editing to literature synthesis, coding, data analysis, manuscript drafting, peer-review-related work, and clinically sensitive research workflows. Generic AI-use declarations are therefore often insufficient, especially when AI use affects scholarly substance, methodology, interpretation, peer-review-related work, or sensitive research materials. Knowing that AI was used does not clarify what task it supported, what information was entered, how outputs were handled, whether claims and references were verified, whether sensitive material was protected, or how human responsibility was maintained.

The framework proposed in this article distinguishes disclosure from documentation. Disclosure communicates the presence of AI use; documentation makes the AI-assisted workflow traceable, inspectable, and accountable. By proposing task-specific, proportionate, role-specific, and privacy-sensitive documentation practices, the framework aims to support responsible AI use within open science while protecting confidential, patient-related, peer-review-related, and methodologically sensitive information. It does not replace journal or publisher policies, but it can help researchers, reviewers, editors, journals, and institutions document AI-assisted workflows more consistently, transparently, and responsibly.

## Supplementary Information

Additional file 1. Supplementary AI-use documentation. Project-specific documentation of the generative AI and AI-assisted tools used during manuscript preparation and framework development, including tool and access information, task-specific use records, prompt documentation, output handling, human verification, data-protection safeguards, and the target-journal policy check.

Additional file 2.

## Data Availability

No datasets were generated or analysed during the current study.

## Abbreviations

AI: Artificial intelligence
LLMs: Large language models
OSF: Open Science Framework
