# Supplementary material for: Disclosure is not documentation: an open science framework for documenting generative AI use in scholarly research and publication workflows
Source: Res Integr Peer Rev. 2026 Aug 15;11:47. doi: 10.1186/s41073-026-00245-8 (PMC13483690; doi:10.1186/s41073-026-00245-8)
Supplement: Supplementary file 1 [file 41073_2026_245_MOESM2_ESM.pdf]

Conduct a mini scoping review on current policies concerning the use of artificial intelligence, especially generative AI, large language models, and tools such as ChatGPT, in scientific research and academic publishing. Objective: Identify and synthesize current requirements from policies, guidelines, editorial policies, publisher policies, reporting guidelines, research integrity guidelines, and recommendations from scientific organizations regarding the disclosure, documentation, and limitation of AI use in research and publication. Research question: What information do current policies require or recommend that researchers disclose when AI tools are used in scientific studies, manuscripts, data analysis, literature searches, peer review, or publication processes? Please consider, in particular, policies and guidelines from: - scientific publishers and journals - publication ethics organizations - editorial organizations - professional societies - research funders - universities or research institutions - reporting or transparency guidelines - systematic reviews or policy analyses on AI use in research and publishing Time frame: Prioritize sources from 2023 to 2026. Include older sources only if they are frequently cited or foundational for later policies. Search terms / concepts: "generative AI" OR "artificial intelligence" OR "large language model" OR "LLM" OR "ChatGPT" OR "AI-assisted writing" OR "AI use disclosure" OR "AI policy" OR "publication ethics" OR "scientific publishing" OR "research integrity" OR "authorship" OR "peer review" OR "transparency statement" OR "methods disclosure" OR "AI-generated content" OR "LLM in research" OR "AI tools in manuscript preparation". Inclusion criteria: - Sources that contain concrete requirements, recommendations, or rules regarding the use of AI in scientific studies or academic publications. - Policies, guidelines, editorial statements, position papers, research integrity statements, reviews, or empirical analyses of policies. - Sources related to scientific writing, study design, data analysis, literature searching, image or text generation, code generation, peer review, authorship, or ethical responsibility. Exclusion criteria: - Purely technical papers on AI without reference to research practice, publishing, or policy. - Opinion pieces that do not contain concrete policy or transparency requirements. - Studies on AI applications in medicine, psychology, or other fields if they do not address disclosure, documentation, or governance of AI use in scientific studies or publications. For each relevant source, extract: 1. Full reference, including year, authors or organization, title, source, DOI or URL. 2. Type of source: policy, editorial guideline, ethics guideline, review, position paper, empirical policy analysis, etc. 3. Scope: manuscript preparation, study design, data collection, data analysis, literature search, image generation, code, peer review, publication, research funding, etc. 4. Type of AI use addressed: gener-

restrict or ban AI use in peer review and image generation—but leave the threshold between permissible language assistance and reportable scientific assistance poorly defined, and provide little guidance on AI use in data analysis, qualitative coding, or synthetic data generation.

Abstract

Current policies across publishers, professional societies, ethics organizations, and research institutions converge on a small set of near-universal requirements for AI disclosure in scientific research and publishing. The prohibition on listing AI tools as authors is essentially unanimous—grounded in the inability of AI to bear moral or legal accountability [1] —and the obligation to disclose AI use when it substantively affects manuscript content or research processes is standard across virtually all major publisher guidelines, WAME recommendations, [2] and ICMJE-aligned policies. The most commonly required disclosure elements are the AI tool name, purpose of use, and a statement of human oversight and responsibility, [3] with tool version, date of use, and specific prompts increasingly required for analytical or content-generating uses. [4] Peer review represents the most restricted domain, with approximately 59–91% of top medical journals prohibiting or severely limiting AI use by reviewers, primarily on confidentiality grounds. [5] AI-generated images face near-universal prohibition or strict restriction in major publishers, [6] while language editing assistance is widely permitted with minimal or no disclosure required. [7]

Despite this normative consensus, substantial implementation gaps remain. Empirical analyses reveal that actual disclosure rates are extremely low—only 4.2% of applied linguistics articles published in 2024–2025 included any AI-related disclosure [8] —and that fewer than half of journals with guidelines specify the required content of disclosures. [3] The distinction between language assistance and substantive scientific assistance, which is policy-critical, is drawn inconsistently or not at all in most journal policies. Methodological documentation of AI use in data analysis, qualitative coding, literature screening, and systematic reviews—for which specialized reporting guidelines now exist (GAMER, [9] TRIPOD-LLM, [10] CHART, [11] REFINE, [12] PROCESS 2025 [13])—is largely absent from standard publisher templates, and almost no journal endorses these domain-specific standards. [14] Enforcement relies entirely on author self-disclosure in the absence of reliable AI-detection tools, [15] and AI use in synthetic data generation, qualitative analysis, and data collection remains minimally regulated by current policies. [16]

Flow Diagram

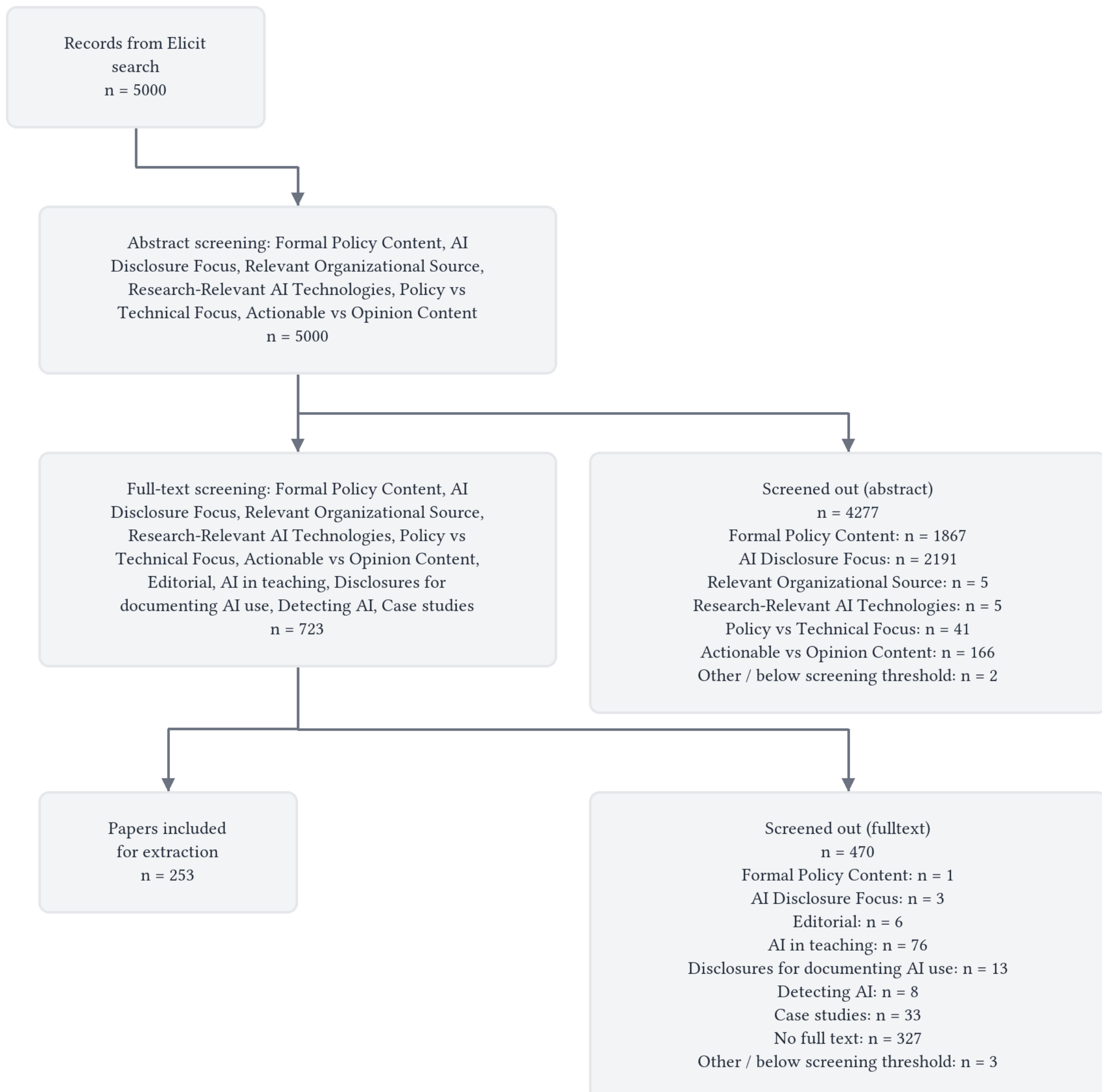

Paper search

We performed a semantic search across over 138 million academic papers from the Elicit search engine, which includes all of Semantic Scholar and OpenAlex.

We ran this query: ”Conduct a mini scoping review on current policies concerning the use of artificial intelligence, especially generative AI, large language models, and tools such as ChatGPT, in scientific research and academic publishing.

Objective: Identify and synthesize current requirements from policies, guidelines, editorial policies, publisher policies, reporting guidelines, research integrity guidelines, and recommendations from scientific organizations regarding the disclosure, documentation, and limitation of AI use in research and publication.

Research question: What information do current policies require or recommend that researchers disclose when AI tools are used in scientific studies, manuscripts, data analysis, literature searches, peer review, or publication processes?

Please consider, in particular, policies and guidelines from:

- scientific publishers and journals
- publication ethics organizations
- editorial organizations
- professional societies
- research funders
- universities or research institutions
- reporting or transparency guidelines
- systematic reviews or policy analyses on AI use in research and publishing

Time frame: Prioritize sources from 2023 to 2026. Include older sources only if they are frequently cited or foundational for later policies.

Search terms / concepts: “generative AI” OR “artificial intelligence” OR “large language model” OR “LLM” OR “ChatGPT” OR “AI-assisted writing” OR “AI use disclosure” OR “AI policy” OR “publication ethics” OR “scientific publishing” OR “research integrity” OR “authorship” OR “peer review” OR “transparency statement” OR “methods disclosure” OR “AI-generated content” OR “LLM in research” OR “AI tools in manuscript preparation”.

Inclusion criteria:

- Sources that contain concrete requirements, recommendations, or rules regarding the use of AI in scientific studies or academic publications.
- Policies, guidelines, editorial statements, position papers, research integrity statements, reviews, or empirical analyses of policies.
- Sources related to scientific writing, study design, data analysis, literature searching, image or text generation, code generation, peer review, authorship, or ethical responsibility.

Exclusion criteria:

- Purely technical papers on AI without reference to research practice, publishing, or policy.
- Opinion pieces that do not contain concrete policy or transparency requirements.
- Studies on AI applications in medicine, psychology, or other fields if they do not address disclosure, documentation, or governance of AI use in scientific studies or publications.

For each relevant source, extract:

1. Full reference, including year, authors or organization, title, source, DOI or URL.
2. Type of source: policy, editorial guideline, ethics guideline, review, position paper, empirical policy analysis, etc.

3. Scope: manuscript preparation, study design, data collection, data analysis, literature search, image generation, code, peer review, publication, research funding, etc.
4. Type of AI use addressed: generative AI, LLMs, chatbots, translation tools, writing assistance, analysis tools, image generators, coding tools, search systems, etc.
5. Information required or recommended for disclosure, for example:
    - name of the AI tool
    - version or model
    - provider
    - date or period of use
    - purpose of use
    - specific research or writing steps in which AI was used
    - prompts or example prompts used
    - extent of AI assistance
    - human review, responsibility, and oversight
    - whether AI-generated content was modified
    - whether AI was involved in data analysis, coding, or interpretation
    - data protection, copyright, or confidentiality issues
    - prohibited or restricted uses
    - authorship requirements or exclusion of AI as an author
    - requirements for peer reviewers
6. Whether the policy excludes AI from authorship.
7. Whether explicit AI disclosure is required in the manuscript, methods section, acknowledgements, a separate statement, or directly to the journal.
8. Whether the policy distinguishes between language assistance and substantive scientific assistance.
9. Whether the policy provides example wording for AI disclosure statements.
10. Relevant short direct quotations, no more than 1–2 sentences per source.
11. Practical relevance for researchers.

Then provide a synthesis with the following sections:

A. Brief overview: Which policy requirements occur most frequently?

B. Summary table: Columns:

- Source / organization
- Year
- Type of policy
- AI use addressed
- Required information
- Location of disclosure
- Authorship rule
- Special restrictions
- Relevance for scientific studies and publications

C. Thematic categories: Group the requirements into categories such as:

1. Transparency and disclosure
2. Authorship and responsibility
3. Methodological documentation

4. Data analysis and interpretation
5. Literature searching and systematic reviews
6. Data protection, confidentiality, and copyright
7. Peer review
8. Image, table, code, and text generation
9. Prohibited or restricted uses

D. Minimal reporting template: Derive a concise checklist of the minimum information researchers should document and report when using AI in scientific studies or publications.

E. Research gaps: Identify which aspects are inconsistent, unclear, or insufficiently regulated across current policies.

F. Output format: Respond in English. Use precise and concise language. Support all claims with citations. Clearly distinguish between statements directly derived from policies and interpretive synthesis.

Prioritize sources that contain primary policies or systematically compare policies. If no primary policy is available for a point, use reviews only as secondary evidence and mark them accordingly."

The search returned 5,000 total results from Elicit.

We retrieved 5000 papers most relevant to the query for screening.

Screening

Abstract screening

We screened in sources based on their abstracts that met these criteria:

- **Formal Policy Content**: Does the document contain formal policies, guidelines, or requirements with concrete, actionable content regarding AI use in scientific research or academic publishing (rather than general discussions or opinions)?
- **AI Disclosure Focus**: Does the document address AI disclosure, documentation, transparency requirements, reporting standards, or governance frameworks for AI use in research processes or publication workflows?
- **Relevant Organizational Source**: Is the document from a relevant organizational source (scientific publishers, journals, editorial organizations, professional societies, research funders, universities, research institutions, or publication ethics organizations)?
- **Research-Relevant AI Technologies**: Does the document address AI technologies relevant to research and publishing (such as generative AI, large language models, AI writing assistants, AI analysis tools, or other AI technologies applicable to scientific research and academic publishing processes)?
- **Policy vs Technical Focus**: Does the document address research practice, ethics, or disclosure requirements (rather than focusing solely on AI algorithm development, performance evaluation, or technical specifications without policy implications)?
- **Actionable vs Opinion Content**: Does the document contain specific, actionable policy recommendations or requirements (rather than being purely editorial opinions, commentaries, or perspective pieces without concrete policy recommendations)?

Papers that failed any strict criterion were automatically excluded. For the remaining papers, we considered all screening questions together and made a holistic judgement about whether to screen in each paper.

723 papers passed abstract screening and moved to full-text screening.

At abstract screening, the number of papers excluded for each primary reason was:

- **Formal Policy Content**: n = 1867
- **AI Disclosure Focus**: n = 2191
- **Relevant Organizational Source**: n = 5
- **Research-Relevant AI Technologies**: n = 5
- **Policy vs Technical Focus**: n = 41
- **Actionable vs Opinion Content**: n = 166
- **Other / below screening threshold**: n = 2

Full-text screening

We then screened papers based on their full text using these additional criteria:

- **Formal Policy Content**: Does the document contain formal policies, guidelines, or requirements with concrete, actionable content regarding AI use in scientific research or academic publishing (rather than general discussions or opinions)?
- **AI Disclosure Focus**: Does the document address AI disclosure, documentation, transparency requirements, reporting standards, or governance frameworks for AI use in research processes or publication workflows?
- **Relevant Organizational Source**: Is the document from a relevant organizational source (scientific publishers, journals, editorial organizations, professional societies, research funders, universities, research institutions, or publication ethics organizations)?
- **Research-Relevant AI Technologies**: Does the document address AI technologies relevant to research and publishing (such as generative AI, large language models, AI writing assistants, AI analysis tools, or other AI technologies applicable to scientific research and academic publishing processes)?
- **Policy vs Technical Focus**: Does the document address research practice, ethics, or disclosure requirements (rather than focusing solely on AI algorithm development, performance evaluation, or technical specifications without policy implications)?
- **Actionable vs Opinion Content**: Does the document contain specific, actionable policy recommendations or requirements (rather than being purely editorial opinions, commentaries, or perspective pieces without concrete policy recommendations)?
- **Editorial**: If the paper is an editorial, exclude it unless it provides guidelines for a journal.
- **AI in teaching**: The manuscript should not serve as a guide for application of AI in teaching at schools or universities, but should explicitly focus on scientific studies.
- **Disclosures for documenting AI use**: Specific disclosures for documenting AI use must be reported.
- **Detecting AI**: It should not be about detecting AI-generated research.
- **Case studies**: Case studies should be excluded.

Papers that failed any strict criterion were automatically excluded. For the remaining papers, we considered all screening questions together and made a holistic judgement about whether to include each paper in the final analysis.

253 papers passed full-text screening and moved to data extraction.

At full-text screening, the number of papers excluded for each primary reason was:

- **Formal Policy Content**: n = 1
- **AI Disclosure Focus**: n = 3
- **Editorial**: n = 6
- **AI in teaching**: n = 76
- **Disclosures for documenting AI use**: n = 13
- **Detecting AI**: n = 8

- **Case studies**: n = 33
- **No full text**: n = 327
- **Other / below screening threshold**: n = 3

Data extraction

We asked a large language model to extract each data column below from each paper. We gave the model the extraction instructions shown below for each column.

- **Source Details**:

  Extract full bibliographic information and source credibility details, including:

- Full citation (authors/organization, year, title, journal/source, DOI/URL)
- Type of source (journal policy, publisher guideline, editorial statement, position paper, ethics guideline, systematic review, etc.)
- Organization type and authority level (major publisher, professional society, funding agency, university, ethics committee, etc.)
- Geographic scope or jurisdiction
- Date of policy implementation or last update

- **AI Use Scope**:

  Extract what types of AI use and research activities are specifically addressed by this policy, including:

- Types of AI tools covered (generative AI, LLMs, ChatGPT, writing assistants, analysis tools, image generators, translation tools, etc.)
- Research/publishing stages covered (study design, data collection, data analysis, literature searches, manuscript writing, peer review, image creation, code generation, etc.)
- Distinction between different levels of AI assistance (grammar checking vs. content generation vs. substantive analysis)
- Whether policy applies to authors, reviewers, editors, or all parties

- **Required Disclosures**:

  Extract specific information that must or should be disclosed when AI tools are used in research or publishing, including:

- AI tool name/brand and version
- Provider/developer information
- Date and duration of use
- Specific purpose and extent of AI assistance
- Research steps where AI was employed
- Prompts or example prompts used
- Whether AI output was modified by humans
- Level of human oversight and review
- Any data protection or confidentiality considerations
- Distinguish between mandatory vs. recommended disclosures

- **Disclosure Location**:

  Extract where AI use disclosures must be placed within manuscripts or submissions, including:

- Specific manuscript sections (methods, acknowledgments, author statement, etc.)
- Whether disclosure appears in published article or only submitted to journal
- Format requirements (separate statement, integrated text, etc.)
- Whether different types of AI use require disclosure in different locations
- Any requirements for disclosure during peer review process

- **Authorship Rules**:

  Extract explicit policies regarding AI and authorship, including:

- Whether AI tools can be listed as authors or co-authors
- Requirements for human accountability and responsibility
- How AI assistance affects authorship criteria
- Whether AI-assisted work requires different authorship disclosures
- Citation requirements for AI tools (if any)
- Responsibility allocation for AI-generated content accuracy

- **Restrictions & Prohibitions**:

  Extract specific limitations, restrictions, or prohibited uses of AI in research and publishing contexts, including:

- Banned AI applications (e.g., image manipulation, data fabrication)
- Contexts where AI use is not permitted (peer review, ethics review, etc.)
- Restrictions on sharing confidential/proprietary information with AI
- Requirements for IRB approval when AI is used in research
- Limitations on AI use for substantive scientific tasks vs. language assistance
- Consequences for undisclosed or inappropriate AI use

- **Template Language**:

  Extract any example disclosure statements, templates, or specific wording provided by the policy for authors to use when disclosing AI use, including:

- Exact template text or example statements
- Variable elements that authors must customize
- Different templates for different types of AI use
- Language for different manuscript sections
- Whether templates are mandatory or suggested
- Instructions for modifying standard templates

- **Policy Implementation**:

  Extract information about how these AI policies are implemented, monitored, and enforced, including:

- Detection methods or tools mentioned
- Editorial review processes for AI disclosure
- Requirements for reviewers regarding AI use
- Training or guidance provided to stakeholders
- Appeals or clarification processes
- Timeline for policy implementation
- References to updating policies as AI evolves

Report

Due to the limitations of the AI model, we are only able to process 200 sources while writing a report. This report was written using the 200 sources that had the highest screening scores out of the 253 sources that we screened in and extracted data from.

Results

Characteristics of Included Sources

The corpus consists of 200 sources published primarily between 2023 and 2026, encompassing a wide range of source types: editorial statements and journal policies, position papers and ethics guidelines from professional organizations, empirical cross-sectional analyses of journal or publisher policies, reporting guideline development papers, systematic and scoping reviews, and university or institutional guidance documents. The sources originate from across biomedicine, nursing, surgery, dentistry, radiology, public health, social science, computer science, ecology, and higher education. Major issuing organizations include the World Association of Medical Editors (WAME), the International Committee of Medical Journal Editors (ICMJE), the Committee on Publication Ethics (COPE), Elsevier, Springer Nature, Nature, Wiley, Taylor & Francis, SAGE, JMIR Publications, and numerous individual journals. Several sources are themselves systematic or scoping analyses of policies rather than primary policies.

Study	Full text retrieved?	Source type	Organization / outlet	Scope
James H. Lubowitz, 2023 [17]	Yes	Editorial guideline	Biomedical journal	Manuscript writing; peer review prohibition
Mohammad Hosseini et al., 2023 [18]	Yes	Peer-reviewed position paper	Research Ethics (SAGE / NIH-affiliated)	Manuscript writing; disclosure framework
Tiffany I. Leung et al., 2023 [19]	Yes	Editorial statement	JMIR Publications	Authorship; peer review; manuscript preparation
Justin Fontenot, 2025 [15]	Yes	Position paper	Nursing editor community	Manuscript writing; data analysis; research workflows
WAME, 2023 (CHATBOTS) [20]	Yes	Professional society guideline	World Association of Medical Editors	All publication stages; authorship; peer review
T. Tulandi, 2023 [21]	Yes	Journal policy	Elsevier / JOGC	Manuscript writing; authorship
P. Krausman, 2023 [7]	Yes	Editorial statement	Journal of Wildlife Management (Wiley)	Writing; analysis; submission
G. Kaebnick et al., 2023 [1]	Yes	Editorial statement	Bioethics/humanities editors	All publication stages
Raymond K. Wong, 2023 [22]	Yes	Editorial statement	JECT journal	Manuscript writing; peer review

Study	Full text retrieved?	Source type	Organization / outlet	Scope
Troy D. Sadler et al., 2024 [23]	Yes	Editorial statement	Journal of Research in Science Teaching	Manuscript writing; data analysis
G. Kaebnick et al., 2023a [24]	Yes	Editorial statement	Bioethics/humanities editors	All publication stages
A. Tang et al., 2023 [25]	Yes	Research paper	Academic researchers	Manuscript writing; peer review
J. González-Rivera, 2026 [26]	Yes	Scoping review with taxonomy	Peer-reviewed journal	Policies 2023–2025; AI-Use-12 framework
G. Cacciamani et al., 2023 [27]	Yes	Guideline development protocol	CANGARU initiative / EQUATOR	All academic stages
Conner Ganjavi et al., 2024 [3]	Yes	Bibliometric / cross-sectional study	BMJ	Publisher and journal GAI policies
Mohammad Hosseini & S. Horbach, 2023 [28]	Yes	Position paper	Research Integrity and Peer Review	Peer review; editorial use of LLMs
David B. Resnik & Mohammad Hosseini, 2025 [29]	Yes	Position paper	NIH / Springer Nature	Mandatory vs. optional disclosure framework
M. Bhargava et al., 2024 [30]	Yes	Editorial	Academic journal (India)	Manuscript; peer review; ICMR context
Bernd Hamm et al., 2024 [31]	Yes	Joint editorial statement	ESR Journals / Radiology Editors' Forum	Medical imaging journals
Sun Huh, 2026 [32]	Yes	Narrative review	Korean editor's perspective	Editors' perceptions; Korean medical journals
Seong Ho Park, 2023 [33]	Yes	Journal policy	Korean Journal of Radiology	Manuscript; peer review
G. Kaebnick et al., 2023b [34]	Yes	Editorial statement	Bioethics/humanities editors	All publication stages
G. Kaebnick et al., 2023c [35]	Yes	Editorial statement	Bioethics/humanities editors (multi-journal)	All publication stages
Ahmed Alduais et al., 2025 [36]	Yes	Policy analysis	Qatar University	Higher education research; 74 policy documents
Gerald Gartlehner et al., 2025 [37]	Yes	Position statement	Cochrane Rapid Reviews Methods Group	Rapid reviews; evidence synthesis

Study	Full text retrieved?	Source type	Organization / outlet	Scope
Chris Zielinski et al., 2023 [2]	Yes	Professional society guideline	WAME (revised May 2023)	All scholarly publication stages
Cortland Linder et al., 2025 [4]	Yes	Consensus guidance	GAIT 2024 Collaborative Group	Scientific writing; CRediT mapping
P. Hacker et al., 2023 [38]	Yes	Legal/regulatory position paper	Academic legal scholars	EU AI Act; regulatory frameworks
Amrita Ganguly et al., 2025 [39]	Yes	Empirical thematic analysis	US R1 universities (n=30)	Researcher guidance from HEIs
Kyle M. L. Jones, 2025 [40]	Yes	Framework paper	Academic researchers	Qualitative research AI disclosure
Jan Odom-Forren, 2023 [41]	Yes	Editorial statement	JoPAN / Elsevier	Manuscript; authorship
Tran Huu Tuyen, 2026 [42]	Yes	Policy-and-practice mapping	Peer-reviewed journal	Vietnamese scholarly publishing
S. Leopold et al., 2023 [43]	Yes	Editorial policy statement	Orthopedic surgery journals	Manuscript preparation
Xufei Luo et al., 2025 [9]	Yes	Reporting guideline (GAMER)	Lanzhou University / WHO CC	Medical research; 9-item checklist
A. De Cassai et al., 2026 [44]	Yes	Delphi consensus	Anaesthesia/pain medicine editors-in-chief (n=53)	Authorship; peer review; editorial workflows
F. Eggmann et al., 2025 [45]	Yes	Cross-sectional review / framework	Academic researchers	Dental peer review; top-50 dental journals
Benjamin Luke Moorhouse et al., 2026 [8]	Yes	Systematic content analysis	Applied linguistics research community	GenAI disclosure conventions in practice
Overview of the Publication Policies, 2023 [46]	Yes	Policy review	Major publishers (EU/USA)	AI policy overview
Ndubuisi Friday Ugwu et al., 2024 [47]	Yes	Rapid review	Higher Learning Research Communications	Research writing ethics
Malik Sallam et al., 2023 [48]	Yes	Checklist development (METRICS)	Interactive Journal of Medical Research	Healthcare AI study reporting
Brett N. Hryciw et al., 2023 [49]	Yes	Position paper	Frontiers in AI	Scientific writing in medicine
Divesh Sardana et al., 2023 [50]	Yes	Position paper	Journal of the American Dental Association	Dental / academic publishing

Study	Full text retrieved?	Source type	Organization / outlet	Scope
J. Zhong et al., 2024 [51]	Yes	Meta-research study	Insights into Imaging / ESR	Radiological journals LLM policies (n=189)
Seong Ho Park, 2026 [52]	Yes	Editorial	Korean Journal of Radiology	Policy landscape overview
F. Kolbinger et al., 2024 [53]	Yes	Systematic review / meta-analysis	Nature Portfolio (Communications Medicine)	Medical AI reporting guidelines (n=26)
Mahjabin Nahar et al., 2024 [54]	Yes	Empirical policy landscape analysis	Academic researchers	CS conference GenAI policies (n=64)
M. Stanbrook et al., 2023 [55]	Yes	Journal policy	CMAJ	Manuscript; peer review
Shinae Yu & H. Hwang, 2025 [56]	Yes	Narrative review	Academic journal	Medical writing ethics
Caroline Gatrell et al., 2024 [57]	Yes	Editorial statement	Journal of Management Studies	Management research; peer review
Chris Zielinski et al., 2023a [58]	Yes	Position paper	WAME (Jan 2023 original)	All scholarly publication stages
Jamie R. Robinson et al., 2025 [59]	Yes	White paper / narrative review	Academic Surgical Congress	Academic surgery; manuscripts; grants
Zhicheng Lin, 2024 [60]	Yes	Position paper / policy proposal	Academic researcher	Scholarly publishing policy framework
Mohamed L. Seghier, 2023 [61]	Yes	Editorial statement	IMA / Wiley	Manuscript readability improvement
David B. Resnik & Mohammad Hosseini, 2024 [16]	Yes	Position paper	Science and Engineering Ethics (Springer)	Research ethics; new guidance
Suhaib Abdurahman et al., 2025 [62]	Yes	Methodological primer	Academic researchers (DARPA-funded)	Social-science LLM research
Yilin Ning et al., 2023 [63]	Yes	Scoping review + checklist (TREGAI)	Multi-institutional academic team	Healthcare AI ethics
Joseph Crawford et al., 2026 [64]	Yes	Editorial statement / journal policy	Journal of University Teaching and Learning Practice	Authorship; scholarly accountability
Xufei Luo et al., 2025a [65]	Yes	Protocol article (GAMER)	Lanzhou University / WHO CC	Medical research reporting guidelines
Announcement on Use of AI, 2025 [66]	Yes	Journal policy	Food Safety journal	Manuscript; peer review

Study	Full text retrieved?	Source type	Organization / outlet	Scope
G. Muga, 2023 [67]	Yes	Editorial statement	EPL (Europhysics Letters)	Physics journal manuscript writing
Liana Penabad-Camacho et al., 2024 [68]	Yes	Guideline / position paper	Revista Electrónica Educare (UNA, Costa Rica)	All publication roles
Susanne C. Brink, 2024 [69]	Yes	Editorial statement	Trends in Plant Science (Elsevier/Cell Press)	Manuscript writing
Robin Lacassin et al., 2025 [70]	Yes	Editorial statement	Tektonika journal	Academic research AI use
Sonja Bjelobaba et al., 2024 [71]	Yes	Systematic analysis	Academic research team	Ethical challenges across research phases
Jake Linardon et al., 2025 [72]	Yes	Survey + guidelines	International Journal of Eating Disorders	Eating disorder research AI use
M. Fareed et al., 2025 [73]	Yes	Systematic review	Frontiers in Digital Health	LLM ethics in healthcare (n=27)
Saif Aldeen et al., 2025 [74]	Yes	Systematic review	Academic researchers	LLM reporting guidelines in healthcare (n=18)
Abdenbi El Harrath, 2025 [75]	Yes	Empirical thematic/content analysis	Academic researcher	Top 50 applied linguistics journals
F. Alnaimat et al., 2024 [76]	Yes	Systematic review	Academic researchers	Reporting standards evolution
B. Doskaliuk et al., 2025 [77]	Yes	Review	Academic researchers	AI in peer review
Jack Gallifant et al., 2025 [10]	Yes	Reporting guideline (TRIPOD-LLM)	Nature Medicine / EQUATOR	LLM healthcare research
Shuhui Yin et al., 2025 [78]	Yes	Cross-sectional study	BMC Medicine	Medical journal GAI guidelines (n=340)
João Frutuoso, 2025 [79]	Yes	Technical report	Individual academic	LLM-assisted clinical trials/prediction models
K. Kirby et al., 2026 [14]	Yes	Cross-sectional analysis	Frontiers in Neurology	Top-100 neurology journals
Xu Le, 2024 [80]	Yes	Systematic review (PRISMA)	Academic researcher	Higher education assessment
Matthieu Guémann & C. Médrinal, 2025 [81]	Yes	Journal policy	European Rehabilitation Journal	Generative AI policies

Study	Full text retrieved?	Source type	Organization / outlet	Scope
Meruyert Aristombayeva et al., 2025 [82]	Yes	Comparative policy review	University researchers (US/EU/Central Asia)	HEI GenAI policies
Bright Huo et al., 2025 [11]	Yes	Reporting guideline (CHART)	Multi-journal joint publication (Lancet, NEJM-AI, etc.)	Chatbot health advice studies
Rawan Ghnemat & Amro Saleh, 2026 [83]	Yes	Systematic review	Academic researchers	Clinical AI / LLMs in healthcare (n=90)
M. Krasnoselskyi et al., 2025 [84]	Yes	Research article	Ukrainian academic journal	Plagiarism and AI in medical research
Chris Zielinski et al., 2023b [85]	Yes	Position paper	WAME (revised)	All scholarly publication stages
A. Khoruzhaya et al., 2026 [86]	Yes	Reporting checklist (MEDAI-LLM-SUMM)	Frontiers in Digital Health	Medical text summarization studies
L. Khoruzha, 2025 [87]	Yes	Research article	Borys Grinchenko Kyiv Metropolitan University	Scientific ethics; Ukraine context
A. BaHammam, 2025 [88]	Yes	Editorial / position paper	Academic researcher (Saudi Arabia)	Tiered disclosure framework
Caradee Y. Wright et al., 2024 [89]	Yes	Guidance document	African Journal Partnership Program	African scholarly publishing
I Used AI in the Drafting…, 2025 [90]	Yes	Editorial statement	SAGE journal	Citation and disclosure guidance
The Editorial Team Frontiers in Preventive Medicine, 2025 [91]	Yes	Journal policy	Frontiers in Preventive Medicine	Manuscript and peer review
S. Park et al., 2025 [92]	Yes	Reporting guideline (MI-CLEAR-LLM 2025)	Academic consortium	LLM accuracy in healthcare
Lilia Raitskaya & Elena Tikhonova, 2023 [93]	Yes	Editorial statement	Journal of Language and Education	Academic integrity; ChatGPT
Yunjo An et al., 2025 [94]	Yes	Mixed-methods empirical study	Springer Nature	Top-50 US university GenAI guidelines
Bright Huo et al., 2025a [95]	Yes	Reporting guideline (CHART)	BMC Medicine / Springer Nature	Chatbot health advice studies

Study	Full text retrieved?	Source type	Organization / outlet	Scope
Joep Cornelissen et al., 2024 [96]	Yes	Editorial essay + policy	Organization Theory (SAGE)	Organization theory theorizing
Ghee Young Noh, 2025 [97]	Yes	Editorial	Health & New Media Research	Health and digital media research
Cameron O'Brien et al., 2025 [98]	Yes	Cross-sectional study	Springer Nature	Family medicine journals (n=40)
Clayton Peterson & Marie-Catherine Deschênes, 2025 [99]	Yes	Institutional position paper	Université du Québec à Trois-Rivières	University AI governance
Kari D. Weaver, 2024 [100]	Yes	Position paper	Academic researcher	AID Framework introduction
Cristian Zahn-Muñoz et al., 2026 [101]	Yes	Quantitative documentary analysis	University of Los Lagos, Chile	Latin American Scopus journals (n=1,119)
Liana Penabad-Camacho et al., 2024a [102]	Yes	Guideline / position paper (Spanish)	Revista Electrónica Educare (UNA)	All publication roles
Oluwaseun Farotimi et al., 2025 [103]	Yes	Journal policy/guidance	Research Synthesis Methods	Systematic reviews; GenAI evaluation manuscripts
G. Kaebnick et al., 2023d [104]	Yes	Editorial statement	Bioethics/humanities editors	All publication stages
J. Young & Jemimah L. Young, 2025 [105]	Yes	Editorial statement	Journal of Urban Mathematics Education	Urban mathematics education publishing
G. Kaebnick et al., 2023e [106]	Yes	Editorial statement	Bioethics/humanities editors	All publication stages
Bright Huo, 2025 [107]	Yes	Reporting guideline (CHART)	Annals of Family Medicine	Chatbot health advice studies
Juan-Daniel Machin-Mastromatteo, 2026 [108]	Yes	Scholarly guidance / recommendations	Revista Estudios de la Información	Editorial policy templates
Rishi Bommasani et al., 2023 [109]	Yes	Research report / index	Stanford CRFM / HAI	Foundation model transparency (n=10 developers)
G. Smith et al., 2024 [110]	Yes	Position paper	Academic research team	LLM use in science (10 rules)
Jessica L. Parker et al., 2023 [111]	Yes	Methodological guidelines	The Qualitative Report	Qualitative interview protocol development
K. Abbasi, 2023 [112]	Yes	Editorial statement	BMJ (aligns with COPE)	Authorship; manuscript AI use

Study	Full text retrieved?	Source type	Organization / outlet	Scope
Mr. Thelwall, 2026 [113]	Yes	Position paper	Academic researcher	Research evaluation; Leiden Manifesto extension
A. Hufton, 2023 [114]	Yes	Editorial statement	Patterns (Elsevier/Cell Press)	Authorship; February 2023 policy
W. Blau et al., 2024 [115]	Yes	Position paper	National Academies / Annenberg	Scientific integrity in generative AI age
R. Agha et al., 2025 [13]	Yes	Reporting guideline update (PROCESS 2025)	EQUATOR Network	Surgical case series AI domain
A. Marušić, 2023 [116]	Yes	Journal policy	Journal of Global Health	WAME-aligned manuscript policy
L. Madeyski et al., 2025 [117]	Yes	Research recommendations	Academic researchers	LLM screening in systematic reviews
L. Lingard et al., 2025 [118]	Yes	Descriptive qualitative study	Academic researchers	HPE editor expectations of AI disclosure (n=18)
B. Miao et al., 2024 [119]	Yes	Reporting checklist update (MI-CLAIM-GEN)	Academic researchers (NIH-supported)	Clinical generative AI research
Tim-Dorian Knöchel et al., 2025 [120]	Yes	Delphi consensus / position paper	International multidisciplinary panel (n=16)	Core principles for responsible GenAI in research
Caio Nery, 2025 [121]	Yes	Editorial statement	Journal of the Foot and Ankle (ABTPé)	Manuscript preparation disclosure
Jan Philip Wahle et al., 2023 [122]	Yes	Position paper / proposal	Academic researchers (Lower Saxony-funded)	AI Usage Cards
S. Beshyah et al., 2026 [123]	Yes	Editorial statement	Ibnosina Journal of Medicine and Biomedical Sciences	Responsible GenAI use
H. Ibrahim et al., 2021 [124]	Yes	Review	EQUATOR Network	AI reporting guidelines in healthcare
Why Nature will not allow..., 2023 [6]	Yes	Publisher policy	Nature	Generative AI in images and video
T. Anthuvan et al., 2025 [125]	Yes	Framework paper	Academic researchers	Workplace BYOAI governance
S. Park et al., 2024 [126]	Yes	Reporting guideline (MI-CLEAR-LLM)	Korean Journal of Radiology	LLM accuracy evaluation in healthcare

Study	Full text retrieved?	Source type	Organization / outlet	Scope
S. Park & C. H. Suh, 2024 [127]	Yes	Reporting guidelines review	Korean Journal of Radiology	AI reporting guidelines 2024 updates
V. Vasconcellos, 2023 [128]	Yes	Editorial / commentary	Oftalmologia (Brazil)	AI co-authorship; Brazilian academic context
David Reeping et al., 2025 [129]	Yes	Position paper	Academic researchers	LLM use in qualitative research
M. Akgün, 2025 [130]	Yes	Position paper	Academic researcher	Authorship identity; Ship of Theseus metaphor
Jack Gallifant et al., 2024 [131]	Yes	Reporting guideline (TRIPOD-LLM)	EQUATOR Network	LLM biomedical research
D. Spennemann, 2023 [132]	Yes	Protocol paper	Academic researcher	Managing AI-generated ephemera / data archiving
Natalie A. Carlson & Vanessa C. Burbano, 2025 [133]	Yes	Research methods / position paper	Academic researchers	LLM data annotation in management research
Transparencia y responsabilidad..., 2025 [134]	Yes	Editorial policy	RELIME journal	Continuous AI policy update
José Carlos Cámara Molina, 2024 [135]	Yes	Editorial statement	STM Integrity Hub analysis	AI in academic research; 32 publishers
I. Mese et al., 2026 [12]	Yes	Reporting guideline (REFINE)	International consortium (57 contributors)	Medical foundation model / LLM studies
Batuhan Selvi & Rabia Sena Akbaba Eser, 2025 [136]	Yes	Systematic analysis	Academic researchers	Digital tools for academic writing (n=46)
Janaína Gularte Cardoso & I. Gammarano, 2025 [137]	Yes	Ethics guideline	Universidade Federal de Santa Catarina	GenAI in Brazilian academic context
Christopher Hollister et al., 2024 [138]	Yes	Journal policy	Communications in Information Literacy	Information literacy journal GenAI policy
Loretta Gasparini et al., 2024 [139]	Yes	Campus survey + recommendations	Australian paediatric campus	LLM governance in hospital/research/teaching

Study	Full text retrieved?	Source type	Organization / outlet	Scope
Ali Boutera, 2025 [140]	Yes	Research paper	Academic researcher	AI challenges in scientific writing; APA policy
Tracy B. E. Omorogiuwa et al., 2025 [141]	Yes	Ethics letter	African Independent Ethics Committee	GenAI ethical use; Africa-centered guidance
Chatbots, IA generativa..., 2024 [142]	Yes	Professional society guideline (Portuguese)	WAME (Brazilian translation)	All scholarly publication stages
Dr. Sixbert Sangwa & Placide Mutabazi, 2025 [143]	Yes	Scoping meta-synthesis	Academic researchers	ODL institutions; Global South (n=50 policies)
Václav Vondrovský, 2024 [144]	Yes	Editorial statement	Archeologické rozhledy	Archaeology journal AI policy
R. Fleurence et al., 2024 [145]	Yes	Reporting guideline (ELEVATE-GenAI)	ISPOR Working Group	HEOR with LLMs
G. Kaebnick et al., 2023f [146]	Yes	Editorial statement	Bioethics/humanities editors	All publication stages
Junic Kim & Hae-Woong Shin, 2026 [147]	Yes	Research paper	Academic researchers	LLM governance for literature review systems
Mike Perkins & Jasper Roe, 2023 [148]	Yes	Thematic analysis (F1000Research)	Academic researchers	Publisher guidelines on AI usage (n=107)
D. H. Solomon et al., 2023 [149]	Yes	Editorial statement	American College of Rheumatology journals	Authorship; AI in medical publishing
A. Aghemo et al., 2023 [150]	Yes	Editorial statement / debate	Liver International (Wiley)	AI in scientific manuscripts
Bastian Burger et al., 2023 [151]	Yes	Editorial guideline	Academic publication (management)	AI in research; systematic literature reviews
Karl A. Nath et al., 2024 [152]	Yes	Editorial statement	Mayo Clinic Proceedings	AI in peer review; medical publishing
Ki-Hyun Jeon, 2026 [153]	Yes	Narrative review	Academic journal	LLM limitations in medical writing
S. Mangold & Margie A. Ream, 2024 [154]	Yes	Position paper	AAMC context	AI in graduate medical education applications
Joe F. Hair & Misty A. Sabol, 2025 [155]	Yes	Journal article / guidance	Academic journal	AI in competitive intelligence research
Carolann Murphy & Florian P Thomas, 2023 [156]	Yes	Editorial statement	Journal of Spinal Cord Medicine (Taylor & Francis)	Journal policy; authorship

Study	Full text retrieved?	Source type	Organization / outlet	Scope
Umar Sodangi & A. Isma'il, 2025 [157]	Yes	Narrative review	Academic journal	GenAI in academic writing
J. Crawford et al., 2023 [158]	Yes	Editorial statement	Journal of University Teaching & Learning Practice	AI in higher education research
Ahmed Alduais et al., 2025a [159]	Yes	Position paper / protocol	Qatar University (QU-IRB approved)	ETHICAL protocol for GenAI in research
Joe Hair & Misty A. Sabol, 2024 [160]	Yes	Journal article / guidance	Academic journal	AI in CI research; GPT model guidance
Charles Alba et al., 2025 [161]	Yes	Empirical policy analysis	Washington University in St. Louis	US university AI policies (top 50)
Zhi-qiang Li et al., 2024 [5]	Yes	Cross-sectional study	Academic researchers (China-funded)	AI in peer review; top-100 medical journals
P. Gurnal & L. Rana, 2025 [162]	Yes	Narrative review + SWOT	Academic researchers	AI and publishing ethics; healthcare context
Adam Cheng et al., 2025 [163]	Yes	Position paper	Springer Nature	AI-assisted academic writing in simulation
A. Moreno & D. Bitterman, 2024 [164]	Yes	Position paper	Academic researchers	Clinical-grade LLM evaluation
Seedwell T. M. Sithole & Syed Fahad Ashraf, 2025 [165]	Yes	Bibliometric + thematic analysis	Academic researchers	Accounting journals AI guidelines (ABDC, n=91)
Bright Huo, 2024 [166]	Yes	Protocol for CHART development	McMaster University (HiREB approved)	Chatbot health advice studies protocol
PM Ntsobi, 2024 [167]	Yes	Systematic mapping review	Academic researcher	ChatGPT in academic writing (n=22 countries)
Yun Dai et al., 2024 [168]	Yes	Scoping review (PRISMA-ScR)	Taylor & Francis	Asian university GenAI policies (n=30)
Ahmad Almufarreh et al., 2025 [169]	Yes	Mixed-methods empirical study	Frontiers in AI (UNICAF ethics)	LLM ethics in academia
Ahmed M. Hanafi et al., 2025 [170]	Yes	Comprehensive narrative review	Academic researchers	GenAI in academic research lifecycle

Study	Full text retrieved?	Source type	Organization / outlet	Scope
Chaka Chaka et al., 2024 [171]	Yes	Environmental review / content analysis	South African academic researchers	SA HEI GenAI policies (n=26)
F. Pesapane et al., 2025 [172]	Yes	Position paper	Academic researchers	LLMs and responsibility in radiology
Bert Seghers & Oldřich Tůma, 2024 [173]	Yes	Position paper	ALLEA / European Federation of Academies	Research integrity norms and AI
A. Goto & K. Katanoda, 2023 [174]	Yes	Editorial statement	Journal of Epidemiology	ChatGPT and authorship
Juan-Daniel Machin-Mastromatteo, 2023 [175]	Yes	Systematic analysis	Revista Estudios de la Información	10 editorial AI policies
R. Fleurence et al., 2024a [176]	Yes	Narrative review / position paper	ISPOR Working Group	Generative AI for HTA
Won Ik Cho et al., 2023 [177]	Yes	Position paper / proposal	Academic researchers	PaperCard framework
Devinder Kaur, 2026 [178]	Yes	Position paper	Individual researcher	LLM impact on research writing
M. Najm & Moustafa Mohamad Najm, 2024 [179]	Yes	Policy proposal	Yemen Journal of Medicine	Medical scholarly publication ethics
X. Luo et al., 2024 [180]	Yes	Protocol article (CHEER)	Lanzhou University / WHO CC	Chatbots in medical research
Alexander Goldberg et al., 2024 [181]	Yes	Empirical research article	Academic researchers / NeurIPS	LLM checklist assistant for conference papers
Benjamin Adams et al., 2024 [182]	Yes	Editorial statement	Journal of Spatial Information Science	Policy banning AI writing except minor grammar
Bright Huo et al., 2025b [183]	Yes	Overview / narrative review	Springer Nature	Reporting guidelines for GAI studies overview
Zhicheng Lin, 2024a [184]	Yes	Position paper	Academic researcher (China-funded)	Practical strategies for ethical AI in research
S. Ahn, 2024 [185]	Yes	Survey study	Academic researcher (NRF Korea)	Korean medical journals GAI guidelines (n=100)
Tim Reason et al., 2025 [186]	Yes	Primer / position paper	Multi-institutional team (BMS-funded)	GenAI in HEOR; primer

Study	Full text retrieved?	Source type	Organization / outlet	Scope
A. Bond et al., 2024 [187]	Yes	Research article / case study	Taylor & Francis	AI use in EIA literature review
Tatiana Chakravorti et al., 2025 [188]	Yes	Mixed-methods survey	Academic researchers (US universities)	Social scientists on AI in research (n=284+15)
Nguyen Duy Phu et al., 2025 [189]	Yes	Legal-policy analysis	Academic researchers	GenAI authorship governance; Vietnam
Basil Ribeiro, 2023 [190]	Yes	Editorial / commentary	Academic commentator (Portuguese)	ChatGPT in scientific publications; Brazil
P. Manyengo, 2024 [191]	Yes	Systematic review (PRISMA)	Academic researcher	AI in HLIs in Tanzania (n=20)
Natalie Cooper et al., 2024 [192]	Yes	Editorial statement	Methods in Ecology and Evolution	Coding with LLMs in ecology research
M. Daoudi, 2025 [193]	Yes	Mixed-methods empirical research	Springer Nature (Mohammed V University)	AI ethics in research and publishing
Paul Andrew Blythe et al., 2025 [194]	Yes	Editorial commentary	Academic journal	GenAI in consumer behavior research
T. Hamoda et al., 2025 [195]	Yes	Position paper	Global Andrology Forum Expert Group	AI in scientific writing; andrology
AI-powered literature search…, 2024 [196]	Yes	Position paper	Academic physicians	AI tools in literature search
Nate Breznau & Hung Hoang Viet Nguyen, 2025 [197]	Yes	Primer / review	F1000Research (DFG-funded)	Introduction to GenAI for academics
Neil M. Carleton & S. Thakkar, 2020 [198]	Yes	Tutorial / perspective	Gastroenterology (AGA / Elsevier)	How to interpret AI studies in gastroenterology
Z. Zrubka et al., 2025 [199]	Yes	Systematic review	Academic researchers (Hungary)	Medical AI reporting guidelines (n=24)
J. W. Allen et al., 2024 [200]	Yes	Position paper	Academic researchers (Wellcome/Novo Nordisk)	LLMs for informed consent in clinical research

The breadth of the corpus reflects both the rapid proliferation of AI-in-publishing guidance since late 2022 and the diversity of disciplines in which such policies now appear. Of the 200 sources, the majority address the manuscript-writing and authorship stages; a smaller but substantial subset address peer review, data analysis, study design, and systematic reviews. Several sources (notably González-Rivera 2026, Ganjavi et al. 2024, Zhong et al. 2024, Yin et

al. 2025, Kirby et al. 2026, Ahn 2024, and Zahn-Muñoz et al. 2026) are themselves empirical analyses of how many journals or institutions have adopted policies and what those policies contain, providing a second-order view of the policy landscape. Reporting guidelines (CHART, GAMER, GAIT, TRIPOD-LLM, REFINE, MEDAI-LLM-SUMM, ELEVATE-GenAI, METRICS, MI-CLEAR-LLM, PROCESS 2025, CONSORT-AI extensions) form a distinct and methodologically rigorous subcategory. Sources from the Global South (Africa, Latin America, Vietnam, Central Asia) are present but demonstrate more limited policy development relative to North American and European institutions.

Thematic Analysis

1. Transparency and Disclosure: The Core Consensus

The single most consistent requirement across all source types is the obligation for authors to disclose when and how AI tools were used. This consensus spans individual journal policies, professional society guidelines, empirical policy analyses, and ethics frameworks without meaningful exception. WAME's revised 2023 recommendations state that "the extent and type of use of chatbots in journal publications should be indicated," [2] and COPE's position, referenced across dozens of sources, holds that AI cannot be an author and that its use must be transparently disclosed. [20] Ganjavi et al. found that among the top 100 journals with guidelines, 99% prohibited listing GAI as an author and essentially all required some form of disclosure. [3] Yin et al.'s cross-sectional study of 340 medical journals found that a higher proportion of top-SJR-ranked journals (95.0%) provided author guidelines compared to non-top-ranked journals (86.7%), with both groups sharing a median of 5 recommendations on a 7-point scale for author guidelines. [78]

The near-universal expectation is, however, considerably weakened by variability in what must actually be disclosed. Ganjavi et al. documented that while 100% of publishers and 99% of journals with guidelines prohibited AI authorship, only 75% of publishers and 43% of journals included specific disclosure criteria. [3] Zhong et al.'s meta-research on 189 radiological journals found that only 43.9% had any LLM policy, and among the policy elements, "potential influence of LLMs" was mentioned by only 4.2% and "section that used LLMs" by 1.6%. [51] Kirby et al.'s analysis of 100 neurology journals found that 97% included an AI-related policy, nearly all prohibited AI authorship (97%) and required disclosure (96%), but endorsement of AI-specific reporting guidelines (e.g., CONSORT-AI, SPIRIT-AI) was found in only 1 journal. [14]

The information most frequently requested when disclosure is required can be organized into a hierarchy. The most consistently required elements are: (1) a statement that AI was used at all; (2) the AI tool name; and (3) the purpose of use. Less commonly specified but increasingly recommended are: tool version, date of use, specific prompts, specific manuscript sections affected, and whether outputs were modified by humans. [26] WAME's recommendations explicitly require "the full prompt used to generate research results, the time and date of query, and the AI tool used and its version" when AI is used for analytical work. [2] The GAMER checklist (Luo et al. 2025) codified nine items: general declaration, GAI tool specifications, prompting techniques, tool's role in the study, declaration of any new GAI model developed, AI-assisted sections in the manuscript, content verification, data privacy, and impact on conclusions. [9] The GAIT 2024 guidance requires the model and version, date of last use, mapping to CRediT roles, specific prompts in a supplement, and an author responsibility statement. [4]

The location of disclosure varies substantially across sources. Common placements include the Methods section (especially when AI influenced data analysis or study conduct), the Acknowledgments section (for writing assistance), a dedicated "AI Use Disclosure" or "Declaration of AI and AI-assisted technologies" section placed before the References, and the submission cover letter. [20, 21, 29] Elsevier's standardized template, widely reproduced across

the corpus, uses the phrasing: "During the preparation of this work the author(s) used [NAME TOOL / SERVICE] in order to [REASON]. After using this tool/service, the author(s) reviewed and edited the content as needed and take(s) full responsibility for the content of the publication." [21] This template or close variants appear in multiple journals' policies including Matthieu Guémann & Médrinal (2025), [81] S. Beshyah et al. (2026), [123] and Sithole & Ashraf (2025). [165] WAME additionally requires disclosure in both the Abstract and the Methods section when AI is used for analysis or code generation, a requirement that adds an indexing dimension beyond mere manuscript transparency. [2]

2. Authorship and Accountability

The prohibition on listing AI tools as authors is the most unambiguous and near-universal position across the entire corpus. WAME, COPE, ICMJE, Nature, Science, and essentially every publisher, journal, and professional society in this review agree that AI tools cannot be named as authors because they cannot satisfy authorship criteria: they cannot take moral or legal responsibility, cannot give final approval, cannot be accountable for the work, and do not have legal standing to sign copyright agreements. [1, 2, 20] This consensus was already evident in early 2023 and has not changed as of the most recent sources in the corpus. [52, 56]

The philosophical grounding for this prohibition is articulated most explicitly by Kaebnick et al. (2023), who argue that "the aspect of accountability that generative AI genuinely lacks is moral responsibility" and that "only persons can be morally responsible." [1] This reasoning connects the authorship prohibition directly to broader frameworks of research integrity: if authors must be accountable for their work, and AI cannot be accountable, then no amount of substantive contribution by AI can confer authorship. [16]

Human accountability for AI-assisted content is consequently emphasized strongly. Authors are described as bearing full responsibility for verifying, correcting, and approving any AI-generated material, including ensuring absence of plagiarism, factual accuracy of citations, and avoidance of fabricated references. [2, 56] Resnik & Hosseini (2025) provide the most analytically precise formulation: disclosure should be mandatory when AI use is "intentional and substantial," defined as making decisions that directly affect research results, generating or synthesizing content, or analyzing content—but optional when AI is used only for grammar/organization/reference checking. [29] This three-level distinction (mandatory, optional, unnecessary) is among the most practically actionable frameworks in the corpus.

A secondary authorship-related issue concerns how AI contributions should be credited if not as authorship. Several sources suggest treating AI tools like software and citing them as such in references, using APA or similar citation formats. [18, 68] The GAIT guidance maps AI contributions to CRediT contributor roles as an alternative to authorship attribution, [4] while Akgün (2025) proposes "expanded contributor declarations" that include AI-specific task descriptions such as prompt design and model oversight. [130]

3. Methodological Documentation

A subset of policies goes beyond simple acknowledgment to require methodological-grade documentation when AI is employed in substantive scientific tasks. The distinction between language assistance and substantive scientific assistance is recognized by a majority of sources as policy-relevant, though the dividing line is drawn differently across policies.

The clearest exemption from disclosure applies to basic grammar, spelling, and reference checking. Wiley's policy, cited in multiple sources, explicitly states that "tools that are used to improve spelling, grammar, and general editing are not included in the scope of these guidelines." [7] Elsevier, Nature, and most other major publishers maintain sim-

ilar carve-outs for basic writing assistance. [6, 69] However, as Fontenot (2025) notes, "the line becomes obfuscated as AI tools increasingly assist with rewriting, paraphrasing, and generating new content powered by AI integration." [15]

When AI is used for data analysis, hypothesis generation, code writing, literature synthesis, or figure creation, the requirement for disclosure shifts from acknowledgment to methodological reporting. WAME mandates that when AI performs analytical work or generates tables, figures, or code, the full prompt, date, and tool version be reported in both the Abstract and Methods. [2] The Cochrane Rapid Reviews Methods Group requires that AI use be specified in the protocol, with model version and prompts documented in a dedicated "AI Use Disclosure" section. [37] The GAMER checklist additionally requires description of whether outputs were verified and what the impact of AI was on conclusions. [9]

Spennemann (2023) addresses a documentation challenge that most policies overlook: the need to archive the raw conversation data from AI interactions, including all regenerations and timestamps, as verifiable research data. [132] Similarly, Abdurahman et al. (2025) argue that for LLMs used in social-science data annotation, reproducibility requires sharing exact prompts, model versions, parameter settings, preprocessing scripts, and a gold-standard human-coded validation subset. [62]

4. Data Analysis and Interpretation

The use of AI for data analysis, statistical modeling, and interpretation of results is addressed inconsistently across policies. Most guidance treats these uses as high-risk and either prohibits them or requires the most detailed disclosure tier. De Cassai et al.'s Delphi consensus among anaesthesia editors explicitly prohibits LLMs from generating "data, references, conclusions, or entire manuscripts." [44] Zahn-Muñoz et al. found that among Latin American journals, autonomous research design, data fabrication, and generating original content were prohibited uses in most policies. [101]

Where data analysis using AI is permitted, the requirement for methodological transparency is most stringent. The MI-CLAIM-GEN checklist (Miao et al. 2024) requires disclosure of all base models, embedding models, and retrieval models; verbatim prompts; output handling; data splits; training-data leakage controls; and reproducibility artifacts. [119] Resnik & Hosseini (2025) specify that when AI collects or analyzes data, authors must disclose "databases searched, search terms, and relevance rules" and analogous procedural details for other AI analyses. [29] The Cochrane Methods Group warns that generative LLMs "can produce incorrect responses, fabricate data or references, perpetuate biases, and spread misinformation" and recommends against fully automating any step of a review. [37]

An important but underregulated area is the use of AI-generated synthetic data. Resnik & Hosseini (2024) propose that researchers using synthetic data must "indicate which parts of the data are synthetic, clearly label the synthetic data, describe how the data were generated, and explain how and why the data were used." [16] This requirement does not appear in most current journal or publisher policies.

5. Literature Searching and Systematic Reviews

The use of AI tools for literature searching occupies an ambiguous position in current policies. A dedicated position paper on AI-powered literature search (2024) identifies four concerns: recommendations of articles from predatory journals, inaccessible citations, lack of transparency in article selection, and AI hallucinations that produce inaccurate summaries. [196] The Cochrane Rapid Reviews Methods Group, while endorsing AI as a quality-assurance tool in single-reviewer workflows, insists that "a human reviewer must remain responsible for verifying all AI outputs and making final decisions." [37]

Farotimi et al. (2025) provide the most detailed guidance for systematic review manuscripts that themselves evaluate GenAI for screening or data extraction, requiring documentation of random seed values, complete confusion matrices, and analysis code. [103] Madeyski et al. (2025) propose that systematic review LLM evaluations should report Lost Evidence/Recall alongside the WMCC metric, report full confusion matrices, and disclose null-output rates. [117]

For literature searches conducted using AI as a tool (rather than evaluated), most policies require little more than acknowledgment that AI tools were used. Ganguly et al. found that US R1 universities advise researchers to include the specific language model and dates of use when AI assists literature work. [39] The WAME guidance requires that when AI tools are used for literature-related tasks, prompts, tool name, and dates be disclosed. [2]

6. Data Protection, Confidentiality, and Copyright

Confidentiality in peer review and data protection in research are among the most consistently addressed special-restriction categories. The prohibition on uploading unpublished manuscripts or proprietary data to AI tools is ubiquitous: WAME explicitly states that "supplying an author's manuscript to a chatbot breaches confidentiality of the submitted manuscript," [20] NIH prohibits use of AI in grant peer review, [39] and Zhi-qiang Li et al. found that 91% of top medical journals with guidance on peer review prohibited uploading manuscript content to AI systems. [5]

The risk of data retention by AI tools is specifically flagged by WAME: "chatbots retain the information supplied to them, including content and prompts, and may use this information in future responses," [2] with the result that confidential manuscript content "could potentially appear in future responses, further increasing the risk of inadvertent plagiarism." [20] This retention risk applies equally to patient data, unpublished research, and commercially sensitive materials. [16, 56]

For research involving human subjects data, several sources identify AI use as requiring attention within existing ethics frameworks. Fontenot (2025) notes that use of AI for qualitative analysis "calls into question various privacy concerns for participants' intimate data" [15] and that IRB or ethics committee approval may be required. Reeping et al. (2025) recommend that researchers be transparent with their IRB and with participants about LLM use when analyzing qualitative data. [129] The REFINE guideline requires disclosure of policies on data storage, retention, and whether data will be reused for model improvement, along with safeguards for protected health information. [12]

Copyright and intellectual property concerns arise in multiple contexts: when AI is trained on copyrighted texts without authorization, when AI-generated outputs may reproduce copyrighted material, and when AI is used to mimic another author's style. Kaebnick et al. note that using AI to mimic "the substance and style of another scholar" raises plagiarism questions "even if no specific passages could be traced to the other scholar." [1] Nature's policy prohibits generative-AI-created or -augmented images precisely because "existing generative AI tools do not provide access to their sources so that such verification can happen." [6]

7. Peer Review

Peer review represents the most restrictive area in current AI governance, with more policies prohibiting or severely limiting AI use in this context than in any other. The rationale is twofold: confidentiality of unpublished manuscripts and concerns about reviewer accountability. Hosseini & Horbach (2023) provide the most extensive analysis, identifying concerns about "the fundamental opacity of LLMs' training data, inner workings, data handling, and development processes" and the risk of amplifying existing biases in epistemic communities. [28]

Li et al.'s cross-sectional study of top-100 medical journals found that 59% of journals with any AI guidance explicitly

banned AI use in peer review, and 91% prohibited uploading manuscript-related content to AI systems. [5] Eggmann et al.'s review of top-50 dental journals revealed that many permit restricted LLM use under specific conditions while others prohibit it or lack explicit policies. [45] Caroline Gatrell et al.'s Journal of Management Studies policy explicitly prohibits "AI's use in peer-reviewers' evaluations of submissions, and regarding editors' handling of manuscripts." [57]

Where AI use by reviewers is permitted, it is typically restricted to light assistance (grammar improvement of the reviewer's own report) with mandatory disclosure. WAME's recommendation that "Editors and peer reviewers should specify, to authors and each other, any use of chatbots in the evaluation of the manuscript and generation of reviews and correspondence" [2] establishes a disclosure obligation even when use is limited. The concern about training data contamination is particularly acute for peer review: as Bhargava et al. note, "by uploading a submitted document into a generative AI tool, there is a real danger of breach in data privacy rights wherever there is personal identifiable information." [30]

B. Doskaliuk et al. enumerate specific tasks for which AI must not be used in peer review: making final judgments on content, evaluating novelty and importance, generating a complete review report, interpreting complex data or statistical analysis, judging ethical standards, and handling confidential content in public AI tools. [77]

8. Image, Table, Code, and Text Generation

AI-generated visual content faces the strictest restrictions. Nature's policy prohibits photographs, videos, and illustrations "created wholly or partly using generative AI" in submitted material, except in pieces specifically about AI. [6] Elsevier and Springer Nature similarly restrict or prohibit AI-generated images. [47] WAME recommends that "all prompts used to generate new text, or to convert text or text prompts into tables or illustrations, should be specified." [20] The González-Rivera scoping review identifies image and figure manipulation as "generally restricted or prohibited unless part of described/reproducible methods." [26]

Code generation by AI occupies a more permissive but still disclosure-required zone. Cooper et al.'s Methods in Ecology and Evolution policy specifies that "portions of code generated by LLMs must be annotated with comments stating that they were generated in this way," [192] and the GAIT guidance maps code generation to the "Formal analysis" or "Software" CRediT role in the GAIT statement. [4]

For text generation, the range of permissiveness is wide. Benjamin Adams et al.'s Journal of Spatial Information Science explicitly bans AI-assisted text generation "except for minor improvement of grammar," [182] citing Nature and Science as precedents. By contrast, most Elsevier, Springer Nature, and Wiley journals permit AI-assisted writing with disclosure, restricting use to "improving readability and language, not to replace key researcher tasks such as interpreting data or drawing scientific conclusions." [21] The RULE-AP consensus among anaesthesia and pain editors states that "LLMs must not generate data, references, conclusions, or entire manuscripts" [44] while permitting language editing.

9. Prohibited and Restricted Uses

Beyond the specific prohibitions covered in other themes, three cross-cutting restrictions appear with high frequency. First, AI cannot be listed as an author under any circumstances, as documented throughout this review. Second, AI must not be used to fabricate, falsify, or manipulate research data; generating synthetic results and presenting them as real, inventing references, or manipulating scientific images are explicitly prohibited by multiple sources and framed as research misconduct. [20, 44, 101] Third, uploading unpublished manuscripts, proprietary data, or personal health information to external AI tools is prohibited or severely restricted, particularly during peer review. [2, 5]

Additional restrictions vary by context. Several policies note that detection tools should not be relied upon as sole enforcement mechanisms: Fontenot (2025) observes that "AI-detecting software continues to produce inaccurate results, even with 100% human-written content," [15] and Selvi & Akbaba Eser (2025) note that Turnitin planned to discontinue its AI detection features. [136] This limitation is significant because it shifts the burden of compliance entirely to author self-disclosure and editorial judgment, making the normative framework largely dependent on good faith.

Summary Table

Source / organization	Year	Type of policy	AI use addressed	Required information	Location of disclosure	Authorship rule	Special restrictions	Relevance for scientific studies and publications
WAME (Zielinski et al.) [2]	2023	Professional society guideline	Chatbots, LLMs, generative AI	Tool name/version, full prompt, date/time of query, extent and type of use [2]	Abstract and Methods for analytical use; Acknowledgments for writing [2]	AI cannot be an author [2]	Uploading manuscripts to AI breaches confidentiality; analytical use requires detailed disclosure [2]	Foundational; widely cited
WAME (Zielinski et al.) [85]	2023	Professional society guideline (revised)	Chatbots, generative AI	Same as above; adds disclosure by editors/reviewers [85]	Acknowledgments; Abstract and Methods [85]	AI cannot be an author [85]	Editors/reviewers must disclose AI use to each other and to authors [85]	Living policy; foundational
WAME (Chatbots, IA generativa...) [142]	2024	Professional society guideline (Portuguese)	Chatbots, generative AI	Version, date/time, complete prompt, extent and type [142]	Not specified; consistent with WAME [142]	AI cannot be an author [142]	Confidentiality; training-data retention risk [142]	Applicable to Portuguese-language scholarly communities

Source / organization	Year	Type of policy	AI use addressed	Required information	Location of disclosure	Authorship rule	Special restrictions	Relevance for scientific studies and publications
Ganjavi et al. [3]	2024	Bibliometric cross-sectional study (n=200)	Generative AI / LLMs	Tool name, version, purpose (75% of publishers); authorship prohibition (96%) [3]	Methods or Acknowledgments; cover letter; new section (Elsevier) [3]	Prohibited by 96–98% of publishers/journals [3]	Heterogeneity; 12 journals conflicted with their own publisher [3]	Empirical baseline of global publisher/journal policy adoption
Resnik & Hosseini, 2025 [29]	2025	Position paper (NIH-affiliated)	All AI tools	Mandatory when intentional and substantial; tool type/name, version, tasks, human oversight [29]	Methods or Introduction; supplements for prompts; acknowledgments for writing [29]	AI cannot be an author [29]	Optional disclosure withheld from reviewers to reduce bias [29]	Provides mandatory/optional/unnecessary disclosure taxonomy
Elsevier / Tulandi, 2023 [21]	2023	Journal policy	AI-assisted writing	Tool name, reason for use; human review statement [21]	Dedicated section "Declaration of AI and AI-assisted technologies" [21]	AI cannot be an author [21]	Only for language improvement, not for key researcher tasks [21]	Widely adopted template

Source / organization	Year	Type of policy	AI use addressed	Required information	Location of disclosure	Authorship rule	Special restrictions	Relevance for scientific studies and publications
Nature (Why Nature…) [6]	2023	Publisher policy	LLMs; image/video generators	LLM use documented in Methods or Acknowledgments; sources for AI-assisted data [6]	Methods or Acknowledgments [6]	LLMs cannot be authors [6]	Generative AI images/videos completely prohibited [6]	High-impact; widely cited
Kaebnick et al. (multiple versions) [1]	2023	Editorial statement	Generative AI, LLMs	Describe how AI was used; identify AI-generated content [1]	Introduction, Methods, appendix, supplemental material, notes or references [1]	Cannot be an author; lacks moral responsibility [1]	Editors should not use AI as sole basis for decisions [1]	Foundational bioethics framing
GAIT 2024 (Linder et al.) [4]	2025	Consensus guidance	Generative AI (all types)	Model/version, date last used, CRediT role mapping, specific prompts in supplement [4]	Methods or Acknowledgments; prompts in supplement [4]	Implied no authorship; authors retain responsibility [4]	No validated way to detect unreported AI plagiarism [4]	Practical CRediT-based approach; 124 participants from 46 countries

Source / organization	Year	Type of policy	AI use addressed	Required information	Location of disclosure	Authorship rule	Special restrictions	Relevance for scientific studies and publications
GAMER (Luo et al.) [9]	2025	Reporting guideline (9-item)	Generative AI in medical research	Tool, version, date, prompting technique, role in study, sections used, verification, privacy, impact on conclusions [9]	Methodology section [9]	Not explicitly stated [9]	Delphi experts from 26 countries; 51 experts [9]	Standardized reporting for medical research using GenAI
CHART (Huo et al.) [11]	2025	Reporting guideline (12 items, 39 subitems)	Generative AI chatbots for health advice	Tool name/version, open/closed-source, route of access, dates/locations of queries, exact prompts, evaluator details, reproducibility [11]	Open science section; CHART checklist submitted with manuscript [11]	Not specified [11]	Scope limited to chatbot health advice studies; RCTs excluded [11]	Living guideline; jointly published in 9 high-impact journals

Source / organization	Year	Type of policy	AI use addressed	Required information	Location of disclosure	Authorship rule	Special restrictions	Relevance for scientific studies and publications
TRIPOD-LLM (Gallifant et al.) [10]	2025	Reporting guideline (19 items, 50 subitems)	LLMs in healthcare research	Model name/version/training cutoff, prompting details, inference settings, evaluation procedures, IRB approval, open-science items [10]	Integrated across all standard manuscript sections [10]	Not specified [10]	Living guideline; panel meets every 3 months [10]	Comprehensive LLM reporting for clinical studies
REFINE (Mese et al.) [12]	2026	Reporting guideline (44 items, 6 sections)	Foundation models and LLMs in medicine	Model identity/version/cutoff, prompts, generation parameters, datasets, human oversight, safety/privacy safeguards [12]	Integrated per section; online checklist [12]	Not specified [12]	57 contributors, 17 countries; updates every 2 years [12]	Most comprehensive guideline for medical FM/LLM research

Source / organization	Year	Type of policy	AI use addressed	Required information	Location of disclosure	Authorship rule	Special restrictions	Relevance for scientific studies and publications
PROCESS 2025 (Agha et al.) [13]	2025	Reporting guideline update	AI in surgical case series	Declaration of AI use; purpose/scope; tool/version/date; data safeguards; human oversight; bias/ethics; reproducibility [13]	Section 5 of PROCESS checklist across relevant manuscript sections [13]	Human authors responsible; AI not listed [13]	49 surgical/scientific experts; 92% response rate [13]	First surgical case series guideline with AI domain
Hosseini et al., 2023 [18]	2023	Position paper	LLMs	Tool name, version, date, user, prompts, affected sections; in-text citations; supplementary materials [18]	Introduction or Methods; in-text citations; reference list; supplements [18]	AI cannot be an author or acknowledged [18]	Bans are unenforceable; argues for citation as software [18]	Proposes APA-style citation format for LLMs
Hosseini & Horbach, 2023 [28]	2023	Position paper	LLMs in peer review	Disclose if LLMs used for reviews; accept responsibility for reports [28]	Journals' websites or decision letters [28]	AI cannot be an author [28]	Confidentiality, risk of biases in review [28]	First systematic analysis of LLM use in peer review

Source / organization	Year	Type of policy	AI use addressed	Required information	Location of disclosure	Authorship rule	Special restrictions	Relevance for scientific studies and publications
Cochrane Rapid Reviews (Gartlehner et al.) [37]	2025	Position statement	LLMs in rapid reviews	Tool, tasks, model version, prompts, verification; AI Use Disclosure section [37]	”AI Use Disclosure” section of Cochrane reports [37]	AI cannot be author; human accountability [37]	Do not fully automate any step; RAISE principles [37]	First Cochrane statement on AI in rapid reviews
González-Rivera, 2026 [26]	2026	Scoping review + AI-Use-12 taxonomy	All AI types	12-item AI-Use-12: tool/provider, version/date, purpose, sections affected, human oversight, verification, prompts, images/code treatment, privacy, IP, compliance [26]	Methods, Acknowledgments, or dedicated section [26]	AI cannot be author; non-delegable human responsibility [26]	Automated detectors insufficient; detectors variable accuracy [26]	Comprehensive policy synthesis 2023–2025; bilingual templates

Source / organization	Year	Type of policy	AI use addressed	Required information	Location of disclosure	Authorship rule	Special restrictions	Relevance for scientific studies and publications
Yin et al., 2025 [78]	2025	Cross-sectional study of medical journals (n=340)	Generative AI	Disclosure and fact-checking required; top-SJR more comprehensive [78]	Varies by journal; some specify Methods, cover letter, acknowledgments [78]	AI authorship prohibited; consensus [78]	Some permit AI for language editing only; data analysis inconsistently addressed [78]	Large-scale empirical analysis of medical journal policies
Zhong et al., 2024 [51]	2024	Meta-research (n=189 radiology journals)	LLMs	Usage, tool name, verification, role; potential influence rarely mentioned [51]	Not standardized across journals [51]	LLMs cannot be authors [51]	Only 43.9% had any policy; ”potential influence” addressed by 4.2% [51]	Reveals policy gaps in radiology specifically
Kirby et al., 2026 [14]	2026	Cross-sectional analysis (n=100 neurology journals)	Generative AI, LLMs	Disclosure required (96%); AI-assisted writing permitted (93%); AI images varied (37% permitted) [14]	Not specified in detail [14]	Prohibited by 97% [14]	Only 1 journal endorsed CONSORT-AI or SPIRIT-AI [14]	Heterogeneous policies despite high adoption rates

Source / organization	Year	Type of policy	AI use addressed	Required information	Location of disclosure	Authorship rule	Special restrictions	Relevance for scientific studies and publications
Li et al., 2024 [5]	2024	Cross-sectional study (top-100 medical journals)	LLMs in peer review	Disclose in review report if AI used; confidentiality [5]	Review report [5]	Not specified (peer review context) [5]	59% banned AI in peer review; 91% prohibited uploading manuscripts [5]	Documents peer review AI prohibition landscape
Zahn-Muñoz et al., 2026 [101]	2026	Quantitative documentary analysis (n=1,119 journals)	Generative AI, chatbots	Disclosure required by 92.6%; location specified by 65.54% [101]	Methods (47.9%), dedicated section (38.1%), cover letter (27.8%) [101]	AI cannot be listed as author [101]	Only 27.2% of Latin American journals had guidelines [101]	Large-scale Latin American policy analysis
Knöchel et al., 2025 [120]	2025	Delphi consensus (n=16 experts)	Generative AI in research	Eight sequential principles: Regulations, Data Security, Quality Control, Originality, Bias Mitigation, Accountability, Transparency, Broader Impact [120]	Transparent reporting in publications [120]	AI cannot be an author [120]	Living guidelines insufficient; field-specific guidance needed [120]	Cross-disciplinary, principle-based framework

Source / organization	Year	Type of policy	AI use addressed	Required information	Location of disclosure	Authorship rule	Special restrictions	Relevance for scientific studies and publications
De Cassai et al. (RULE-AP) [44]	2026	Delphi consensus (n=53 editors-in-chief)	LLMs in anaesthesia/pain journals	Tool name, version, access date, scope of use [44]	Cover letter; Methods section [44]	AI cannot be author [44]	LLMs must not generate original content, data, references, conclusions, or entire manuscripts [44]	Specialty-specific consensus for anaesthesia/pain
ELEVATE-GenAI (Fleurence et al.) [145]	2024	Reporting guideline (ISPOR)	LLMs in HEOR	Model characteristics, accuracy, reproducibility, fairness/bias, deployment/efficiency, calibration, security/privacy [145]	Checklist submitted with manuscript [145]	Not specified [145]	Applies only when LLMs play substantive role in HEOR [145]	Field-specific guideline for health economics/outcomes research
APA Policy / Boutera, 2025 [140]	2023	Professional society policy	LLMs in scientific writing	Prompt and response quoted; tool name, version, company, conversation link [140]	Methods or Introduction [140]	AI cannot be author [140]	Reviewers must not enter drafts into AI tools [140]	APA citation format for AI outputs

Source / organization	Year	Type of policy	AI use addressed	Required information	Location of disclosure	Authorship rule	Special restrictions	Relevance for scientific studies and publications
Penabad-Camacho et al., 2024 [68]	2024	Guideline (editors, reviewers, authors)	All AI types	Model name/version/dates, prompts (initial, secondary, complementary), filtered use; bias mitigation; data protection [68]	All manuscript sections; method flowchart; table/figure notes; disclosures section [68]	AI cannot be author [68]	Unfiltered AI outputs not acceptable; editors must notify authors of AI use [68]	Comprehensive framework including prompt-net concept
Fontenot, 2025 [15]	2025	Position paper (nursing editors)	LLMs, ChatGPT, grammar tools	Detailed scope disclosure; IRB confirmation for data analysis use [15]	Within manuscript; transparency section [15]	AI cannot be author [15]	AI detectors inaccurate; decision-making framework for editors [15]	Framework for nurse editors
Tang et al., 2023 [25]	2023	Research paper (n=125 nursing journals)	Generative AI	Declaration of AI tool usage in manuscript body [25]	Separate declaration section [25]	AI cannot be author [25]	Only 37.6% of nursing journals required explicit AI statements in 2023 [25]	Baseline prevalence study

Source / organization	Year	Type of policy	AI use addressed	Required information	Location of disclosure	Authorship rule	Special restrictions	Relevance for scientific studies and publications
Ahn, 2024 [185]	2024	Survey (n=100 Korean medical journals)	GAI tools	100% of journals required declaration; technical details (tool, version, manufacturer) required by 27.8% [185]	Main manuscript (83.3%) [185]	Prohibited by 94.4% [185]	Only 18% had GAI guidelines in 2024 Q1 among Korean journals [185]	Korean journal landscape; global comparison
Moorhouse et al., 2026 [8]	2026	Systematic content analysis (n=3,384 articles)	Generative AI (ChatGPT, Grammarly)	Tool name/version, purpose, stage/scope, human oversight clause [8]	Methods or Acknowledgments subsection [8]	AI cannot be author [8]	Only 4.2% of articles included any AI disclosure; 2.8% declared use [8]	Empirical evidence of extremely low disclosure rates in practice

---

Synthesis

The 200 sources reviewed present a landscape of rapid normative development alongside persistent implementation deficits. The apparent contradiction between widespread policy adoption and low actual disclosure rates is not merely a mystery to be noted but can be resolved by examining the specific conditions under which disclosure rates are measured.

The most striking empirical finding is from Moorhouse et al. (2026), who found that only 4.2% of 3,384 applied linguistics articles published in 2024–2025 included any AI-related disclosure. [8] This is not necessarily evidence of widespread policy violation. Rather, it reflects three concurrent factors. First, many journals allow non-disclosure for language-only assistance, which is among the most common uses. [8] Second, policy enforcement relies entirely on author self-disclosure in the absence of reliable detection. [15] Third, policy-driven clustering is real—Moorhouse et al. found that disclosure was concentrated in journals with explicit requirements, confirming that mandatory policies do shift behavior. [8]

BaHammam (2025) provides the most precise analytical framework for explaining when disclosure does and does not occur: researchers avoid disclosure because policies are unclear about what triggers the requirement, because disclosure may carry perceived reputational costs, and because the benefit of disclosing minor language assistance is invisible to readers. [88] The solution he proposes—a four-level tiered framework ranging from no disclosure for basic proofreading to comprehensive disclosure with prompts and model versions for primary material generation—addresses exactly this ambiguity. [88]

This tiered logic maps well onto the mandatory/optional/unnecessary distinction of Resnik & Hosseini (2025), who anchor mandatory disclosure to intentionality and substantiality—specifically, whether AI makes decisions affecting research results, generates or synthesizes content, or analyzes content. [29] Policies that do not make this distinction—which is the majority—create a compliance burden for minor uses and undersupport documentation for consequential ones. The González-Rivera (2026) AI-Use-12 framework is the most comprehensive operationalization of this distinction, codifying 12 items ranging from tool/provider identification to IP compliance and peer-review prohibition confirmation. [26]

A second structural heterogeneity concerns the difference between policies primarily focused on text-generation transparency versus policies focused on methodological reproducibility. The Elsevier template and its derivatives address the former: they ask that authors name the tool and state the reason for use. [21] The reporting guidelines (GAMER, TRIPOD-LLM, REFINE, CHART, ELEVATE-GenAI, PROCESS 2025) address the latter: they require prompts, parameters, validation procedures, and data provenance documentation. [9, 10, 12] These two traditions have largely developed in parallel, and most individual journals have adopted the template-based approach without engaging with the methodological-documentation tradition. This gap is especially significant for systematic reviews, meta-analyses, and clinical prediction studies, where AI use in screening or data extraction can fundamentally affect findings without being visible in a simple "tool was used for language editing" disclosure.

The peer review domain presents the sharpest enforcement paradox. The prohibition on uploading manuscripts to AI tools during peer review is nearly universal in formal guidance. [5] Yet there is no reliable mechanism for detecting whether this prohibition is honored, and reviewer surveys suggest actual use is not negligible. [28] Hosseini & Horbach's (2023) analysis identifies the systemic pressure: reviewer fatigue, increasing submission volumes, and the appeal of AI-assisted efficiency create conditions where the ban may be widely circumvented. [28] The constructive response—requiring reviewer disclosure of any AI use while prohibiting manuscript uploads—appears in WAME, CMAJ, and an increasing number of journals, [2, 55] but monitoring remains aspirational.

Data-analysis AI use, particularly for qualitative research, represents the largest underregulated area. Fontenot (2025) flags it as requiring IRB-level attention. [15] Jones (2025) proposes a 20-question heuristic framework (TROUT-AI) that addresses the AI literacy of the research team, AI involvement in participant communication and informed consent, AI's role in sampling strategies, and detailed documentation of human-AI interaction in coding and thematic categorization. [40] This framework substantially exceeds current publisher requirements, which rarely address qualitative research AI use at all. Similarly, synthetic data generation by AI—an increasingly common technique—is addressed only by Resnik & Hosseini (2024) among the sources reviewed. [16]

Minimal Reporting Checklist

Based on convergent requirements across the included sources, particularly the WAME guidelines, [2] Elsevier's template, [21] the GAIT guidance, [4] the GAMER checklist, [9] and the González-Rivera AI-Use-12, [26] the following represents the minimum information researchers should document and report when using AI in scientific studies or

publications.

Trigger: Disclosure is required whenever AI use is intentional and substantially affects the content or process of the research or manuscript—i.e., when AI generates or processes content, data, or images, or when AI makes decisions that affect research results. [29] Basic grammar/spell-check is generally exempted. [7]

The minimum items to report are:

- Name and brand of the AI tool used (e.g., ”ChatGPT”, ”Gemini”, ”Claude”) [20]
- Version or model identifier and date of use (e.g., ”GPT-4o, November 2024”) [4]
- Provider/developer of the tool [2]
- Specific purpose for which AI was used [21]
- Manuscript section(s) or research stage(s) where AI was employed [20]
- Prompts or representative prompts used, particularly when AI generated content affecting results (may be placed in supplementary materials) [4]
- Statement that AI outputs were reviewed and edited by humans and that authors take full responsibility for the content [21]
- Confirmation that AI was not used in peer review and that no confidential manuscript or proprietary data was uploaded to AI tools [26]
- For studies using AI in data analysis, coding, screening, or interpretation: a description of validation procedures, comparison to human benchmarks, and how AI-influenced conclusions were verified [9]
- For studies involving sensitive or personal data: a statement on data protection measures taken when using AI tools [9]

For reporting guidelines applicable to specific study types, additional domain-specific checklists are available: CHART for chatbot health advice studies, [11] TRIPOD-LLM for LLM development/evaluation studies, [10] REFINE for foundation model studies in medicine, [12] GAMER for general medical research using GenAI, [9] ELEVATE-GenAI for HEOR research, [145] and PROCESS 2025 for surgical case series. [13]

References

1. Kaebnick G, Magnus D, Kao A, et al (2023) Editors' statement on the responsible use of generative artificial intelligence technologies in scholarly journal publishing. Developing World Bioethics. https://doi.org/10.1111/dewb.12424

2. Zielinski C, Winker MA, Aggarwal R, et al (2023) Chatbots, generative AI, and scholarly manuscripts: WAME recommendations on chatbots and generative artificial intelligence in relation to scholarly publications. Current Medical Research and Opinion. https://doi.org/10.1080/03007995.2023.2286102

3. Ganjavi C, Eppler M, Pekcan A, et al (2024) Publishers’ and journals’ instructions to authors on use of generative artificial intelligence in academic and scientific publishing: bibliometric analysis. British medical journal. https://doi.org/10.1136/bmj-2023-077192

4. Linder C, Nepogodiev D, Group G2024C (2025) Generative Artificial Intelligence Transparency in scientific writing: the GAIT 2024 guidance. Impact Surgery. https://doi.org/10.62463/surgery.134

5. Li Z, Xu H, Cao H, et al (2024) Use of Artificial Intelligence in Peer Review Among Top 100 Medical Journals. JAMA Network Open. https://doi.org/10.1001/jamanetworkopen.2024.48609

6. (2023) Why Nature will not allow the use of generative AI in images and video. Nature. https://doi.org/10.1038/d41586-023-01546-4

7. Krausman P (2023) Managing artificial intelligence. Journal of Wildlife Management. https://doi.org/10.1002/jwmg.22492

8. Moorhouse BL, Nejadghanbar H, Wu C, et al (2026) Emerging conventions in GenAI disclosure: how applied linguistics scholars disclose AI use. Applied Linguistics Review. https://doi.org/10.1515/applirev-2025-0291

9. Luo X, Tham Y, Giuffré M, et al (2025) Reporting guideline for the use of Generative Artificial intelligence tools in MEdical Research: the GAMER Statement. BMJ evidence-based medicine. https://doi.org/10.1136/bmjebm-2025-113825

10. Gallifant J, Afshar M, Ameen S, et al (2025) The TRIPOD-LLM reporting guideline for studies using large language models. Nature Medicine. https://doi.org/10.1038/s41591-024-03425-5

11. Huo B, Collins GS, Chartash D, et al (2025) Reporting Guideline for Chatbot Health Advice Studies: The CHART Statement. JAMA Network Open. https://doi.org/10.1001/jamanetworkopen.2025.30220

12. Mese I, D'Antonoli TA, Bluethgen C, et al (2026) Reporting checklist for foundation and large language models in medical research (REFINE): an international consensus guideline. Diagnostic and Interventional Radiology. https://doi.org/10.4274/dir.2026.263812

13. Agha R, Mathew G, Rashid R, et al (2025) Revised Preferred Reporting of Case Series in Surgery (PROCESS) Guideline: An update for the age of Artificial Intelligence. Premier Journal of Science. https://doi.org/10.70389/pjs.100080

14. Kirby K, Calvert N, Rowsey K, et al (2026) Artificial intelligence policies in neurology journals: a cross-sectional analysis. Frontiers in Neurology. https://doi.org/10.3389/fneur.2026.1766696

15. Fontenot J (2025) Artificial Intelligence Disclosure in Academic Nursing: A Framework for Editorial Policy and Practice. Nurse Author & Editor. https://doi.org/10.1111/nae2.70002

16. Resnik DB, Hosseini M (2024) The ethics of using artificial intelligence in scientific research: new guidance needed for a new tool. AI and Ethics. https://doi.org/10.1007/s43681-024-00493-8

17. Lubowitz JH (2023) Guidelines for the Use of Generative Artificial Intelligence Tools for Biomedical Journal Authors and Reviewers. Arthroscopy: The Journal of Arthroscopy And Related. https://doi.org/10.1016/j.arthro.2023.10.037

18. Hosseini M, Resnik D, Holmes KL (2023) The ethics of disclosing the use of artificial intelligence tools in writing scholarly manuscripts. Research ethics. https://doi.org/10.1177/17470161231180449

19. Leung TI, Cardoso T de A, Mavragani A, Eysenbach G (2023) Best Practices for Using AI Tools as an Author, Peer Reviewer, or Editor. Journal of Medical Internet Research. https://doi.org/10.2196/51584

20. (2023) CHATBOTS, GENERATIVE AI, AND SCHOLARLY MANUSCRIPTS WAME RECOMMENDATIONS ON CHATBOTS AND GENERATIVE ARTIFICIAL INTELLIGENCE IN RELATION TO SCHOLARLY PUBLICATIONS. JPMI - Journal of Postgraduate Medical Institute. https://doi.org/10.54079/jpmi.37.3.3284

21. Tulandi T (2023) Disclosure of Artificial Intelligence/ChatGPT-Generated Manuscripts. Journal of Obstetrics and Gynaecology Canada. https://doi.org/10.1016/j.jogc.2023.03.013

22. Wong RK (2023) Role of generative artificial intelligence in publishing. What is acceptable, what is not. The journal of extra-corporeal technology. https://doi.org/10.1051/ject/2023033

23. Sadler TD, Mensah F, Tam JS (2024) Artificial intelligence and the Journal of Research in Science Teaching. Journal of Research in Science Teaching. https://doi.org/10.1002/tea.21933

24. Kaebnick G, Magnus D, Kao A, et al (2023) Editors' statement on the responsible use of generative artificial intelligence technologies in scholarly journal publishing. Bioethics. https://doi.org/10.1111/bioe.13220

25. Tang A, Li K, Kwok K, et al (2023) The importance of transparency: Declaring the use of generative artificial intelligence (AI) in academic writing. Journal of Nursing Scholarship. https://doi.org/10.1111/jnu.12938

26. González-Rivera J (2026) How to Report the Use of Artificial Intelligence in Scientific Articles: A Scoping Review and Taxonomy of Editorial Policies. Revista Caribeña de Psicología. https://doi.org/10.37226/rcp.v10i1.17451

27. Cacciamani G, Eppler M, Ganjavi C, et al (2023) Development of the ChatGPT, Generative Artificial Intelligence and Natural Large Language Models for Accountable Reporting and Use (CANGARU) Guidelines. arXivorg. https://doi.org/10.48550/arXiv.2307.08974

28. Hosseini M, Horbach S (2023) Fighting reviewer fatigue or amplifying bias? Considerations and recommendations for use of ChatGPT and other large language models in scholarly peer review. Research Integrity and Peer Review. https://doi.org/10.1186/s41073-023-00133-5

29. Resnik DB, Hosseini M (2025) Disclosing artificial intelligence use in scientific research and publication: When should disclosure be mandatory, optional, or unnecessary? Accountability in Research. https://doi.org/10.1080/08989621.2025.2481949

30. Bhargava M, Bhardwaj P, Dasgupta R (2024) Artificial Intelligence in Biomedical Research and Publications: It is not about Good or Evil but about its Ethical Use. Indian Journal of Community Medicine. https://doi.org/10.4103/ijcm.ijcm_560_24

31. Hamm B, Martí-Bonmatí L, Sardanelli F (2024) ESR Journals editors' joint statement on Guidelines for the Use of Large Language Models by Authors, Reviewers, and Editors. European Radiology. https://doi.org/10.1007/s00330-023-10511-8

32. Huh S (2026) How editors perceive the use of generative artificial intelligence in writing academic papers: a narrative review. Journal of Korean Medical Association. https://doi.org/10.5124/jkma.26.0008

33. Park SH (2023) Use of Generative Artificial Intelligence, Including Large Language Models Such as ChatGPT, in Scientific Publications: Policies of KJR and Prominent Authorities. Korean Journal of Radiology. https://doi.org/10.3348/kjr.2023.0643

34. Kaebnick G, Magnus D, Kao A, et al (2023) Editors' Statement on the Responsible Use of Generative AI Technologies in Scholarly Journal Publishing. American Journal of Bioethics. https://doi.org/10.1080/15265161.2023.2292437

35. Kaebnick G, Magnus D, Kao A, et al (2023) Editors' Statement on the Responsible Use of Generative AI Technologies in Scholarly Journal Publishing. AJOB Neuroscience. https://doi.org/10.1080/21507740.2023.2257181

36. Alduais A, Qadhi S, Chaaban Y, Khraisheh M (2025) Utilizing Generative AI Responsibly and Ethically for Research Purposes in Higher Education: A Policy Analysis. Serials review. https://doi.org/10.1080/00987913.2025.2581429

37. Gartlehner G, Nussbaumer-Streit B, Hamel C, et al (2025) Responsible Integration of Artificial Intelligence in Rapid Reviews: A Position Statement From the Cochrane Rapid Reviews Methods Group. Cochrane evidence synthesis and methods. https://doi.org/10.1002/cesm.70063

38. Hacker P, Engel A, Mauer M (2023) Regulating ChatGPT and other Large Generative AI Models. Conference on Fairness, Accountability and Transparency. https://doi.org/10.1145/3593013.3594067

39. Ganguly A, Johri A, Ali A, McDonald N (2025) Generative artificial intelligence for academic research: evidence from guidance issued for researchers by higher education institutions in the United States. AI and Ethics. https://doi.org/10.1007/s43681-025-00688-7

40. Jones KML (2025) Generative AI in Qualitative Research and Related Transparency Problems: A Novel Heuristic for Disclosing Uses of AI. International Journal of Qualitative Methods. https://doi.org/10.1177/16094069251404329

41. Odom-Forren J (2023) The Role of ChatGPT in Perianesthesia Nursing. Journal of Perianesthesia Nursing. https://doi.org/10.1016/j.jopan.2023.02.006

42. Tuyen TH (2026) RESPONSIBLE USE OF AI-GENERATED CONTENT IN VIETNAMESE SCHOLARLY PUBLISHING: EVIDENCE FROM JOURNAL POLICIES AND EDITORIAL PRACTICES. Veredas do Direito. https://doi.org/10.18623/rvd.v23.n1.4029

43. Leopold S, Haddad F, Sandell LJ, Swiontkowski M (2023) Artificial intelligence applications and scholarly publication in orthopedic surgery. Journal of Orthopaedic Research. https://doi.org/10.1002/jor.25566

44. Cassai AD, Dost B, Augoustides JG, et al (2026) Responsible use of large language models in manuscript authorship, peer review, and editorial processes: a Delphi consensus among editors-in-chief of anaesthesia and pain medicine journals (RULE-AP). British Journal of Anaesthesia. https://doi.org/10.1016/j.bja.2026.01.029

45. Eggmann F, Hildebrand H, Bornstein M (2025) Who reviewed this? Toward responsible integration of large language models for peer review of scientific articles in dental medicine. Swiss dental journal. https://doi.org/10.61872/sdj-2025-03-01

46. (2023) Overview of the Publication Policies of Major Publishers in Europe and the USA Related to the Use of AI Technology. Research Papers. https://doi.org/10.37075/rp.2023.4.05

47. Ugwu NF, Igbinlade AS, Ochiaka RE, et al (2024) Clarifying Ethical Dilemmas of Using Artificial Intelligence in Research Writing: A Rapid Review. Higher Learning Research Communications. https://doi.org/10.18870/hlrc.v142.1549

48. Sallam M, Barakat M, Sallam M (2023) A Preliminary Checklist (METRICS) to Standardize the Design and Reporting of Studies on Generative Artificial Intelligence–Based Models in Health Care Education and Practice: Development Study Involving a Literature Review. Interactive Journal of Medical Research. https://doi.org/10.2196/54704

49. Hryciw BN, Seely AJE, Kyeremanteng K (2023) Guiding principles and proposed classification system for the responsible adoption of artificial intelligence in scientific writing in medicine. Frontiers Artif Intell. https://doi.org/10.3389/frai.2023.1283353

50. Sardana D, Fagan TR, Wright JT (2023) ChatGPT: A disruptive innovation or disrupting innovation in academia? The Journal of the American Dental Association (1939). https://doi.org/10.1016/j.adaj.2023.02.008

51. Zhong J, Xing Y, Hu Y, et al (2024) The policies on the use of large language models in radiological journals are lacking: a meta-research study. Insights into Imaging. https://doi.org/10.1186/s13244-024-01769-7

52. Park SH (2026) Large Language Models in Scientific Publishing: Policy Landscape for Authors, Reviewers, and Editors. Korean Journal of Radiology. https://doi.org/10.3348/kjr.2026.0166

53. Kolbinger F, Veldhuizen GP, Zhu J, et al (2024) Reporting guidelines in medical artificial intelligence: a systematic review and meta-analysis. Communications Medicine. https://doi.org/10.1038/s43856-024-00492-0

54. Nahar M, Lee S, Guillen B, Lee D (2024) Generative AI Policies under the Microscope: How CS Conferences Are Navigating the New Frontier in Scholarly Writing. arXivorg. https://doi.org/10.48550/arXiv.2410.11977

55. Stanbrook M, Weinhold M, Kelsall D (2023) A new policy on the use of artificial intelligence tools for manuscripts submitted to CMAJ. Canadian Medical Association Journal. https://doi.org/10.1503/cmaj.230949

56. Yu S, Hwang H (2025) The ethics of using artificial intelligence in writing medical research papers. Kosin Medical Journal. https://doi.org/10.7180/kmj.25.141

57. Gatrell C, Muzio D, Post C, Wickert C (2024) Here, there and Everywhere: On the Responsible Use of Artificial Intelligence (AI) in Management Research and the Peer-Review Process. Journal of Management Studies. https://doi.org/10.1111/joms.13045

58. Zielinski C, Winker MA, Aggarwal R, et al (2023) Chatbots, ChatGPT, and Scholarly Manuscripts: WAME Recommendations on ChatGPT and Chatbots in relation to scholarly publications. National Medical Journal of India. https://doi.org/10.21608/aeji.2023.282936

59. Robinson JR, Stey A, Schneider DF, et al (2025) Generative Artificial Intelligence in Academic Surgery: Ethical Implications and Transformative Potential. Journal of Surgical Research. https://doi.org/10.1016/j.jss.2024.12.059

60. Lin Z (2024) Towards an AI policy framework in scholarly publishing. Trends in Cognitive Sciences. https://doi.org/10.1016/j.tics.2023.12.002

61. Seghier ML (2023) Using ChatGPT and other AI-assisted tools to improve manuscripts readability and language. International journal of imaging systems and technology (Print). https://doi.org/10.1002/ima.22902

62. Abdurahman S, Ziabari AS, Moore AK, et al (2025) A Primer for Evaluating Large Language Models in Social-Science Research. Advances in Methods and Practices in Psychological Science. https://doi.org/10.1177/25152459251325174

63. Ning Y, Teixayavong S, Shang Y, et al (2023) Generative Artificial Intelligence in Healthcare: Ethical Considerations and Assessment Checklist. arXivorg. https://doi.org/10.48550/arXiv.2311.02107

64. Crawford J, Purvis A, Grieve A, Taylor L (2026) Authorship Statement for Generative Artificial Intelligence: Assuring Trust and Accountability. Journal of University Teaching and Learning Practice. https://doi.org/10.53761/v16abt43

65. Luo X, Tham Y, Daher M, et al (2025) Generative Artificial Intelligence Tools in Medical Research (GAMER): Protocol for a Scoping Review and Development of Reporting Guidelines. JMIR Research Protocols. https://doi.org/10.2196/64640

66. (2025) Announcement on Use of AI. Food Safety. https://doi.org/10.14252/foodsafetyfscj.d-25-00010

67. Muga G (2023) Editorial —Artificial Intelligence language models in scientific writing. Europhysics letters. https://doi.org/10.1209/0295-5075/ace3ef

68. Penabad-Camacho L, Morera-Castro M, Penabad-Camacho MA (2024) Guidelines for the use and reporting of Artificial Intelligence in scientific-academic journals. Revista Electrónica Educare. https://doi.org/10.15359/ree.28-s.21028

69. Brink SC (2024) Rise of the machines: artificial intelligence in plant science and publishing. Trends in Plant Science. https://doi.org/10.1016/j.tplants.2024.01.001

70. Lacassin R, Doré T, Gouiza M, et al (2025) Use of Artificial Intelligence in Academic Research: What Is Acceptable and What is Not. Tektonika. https://doi.org/10.55575/tektonika2025.3.2.119

71. Bjelobaba S, Waddington L, Perkins M, et al (2024) Research Integrity and GenAI: A Systematic Analysis of Ethical Challenges Across Research Phases. arXivorg. https://doi.org/10.48550/arXiv.2412.10134

72. Linardon J, Thomas JJ, Crow S, et al (2025) Conducting Eating Disorder Research in the Era of Generative AI: Researcher Perspectives and Guidelines From the International Journal of Eating Disorders. International Journal of Eating Disorders. https://doi.org/10.1002/eat.24543

73. Fareed M, Fatima M, Uddin J, et al (2025) A systematic review of ethical considerations of large language models in healthcare and medicine. Frontiers Digit Health. https://doi.org/10.3389/fdgth.2025.1653631

74. Aldeen S, Alryalat, Sultan I (2025) Systematic Review of Reporting guidelines for large language models used in healthcare research. High Yield Medical Reviews. https://doi.org/10.59707/hymruxpx7081

75. Harrath AE (2025) Navigating the New Frontier: An Analysis of Applied Linguistics Journals' Editorial Policies on AI Use in Scientific Publishing. International Journal of Linguistics and Translation Studies. https://doi.org/10.36892/ijlts.v6i3.593

76. Alnaimat F, Al-Halaseh S, Alsamhori ARF (2024) Evolution of Research Reporting Standards: Adapting to the Influence of Artificial Intelligence, Statistics Software, and Writing Tools. Journal of Korean medical science. https://doi.org/10.3346/jkms.2024.39.e231

77. Doskaliuk B, Zimba O, Yessirkepov M, et al (2025) Artificial Intelligence in Peer Review: Enhancing Efficiency While Preserving Integrity. Journal of Korean medical science. https://doi.org/10.3346/jkms.2025.40.e92

78. Yin S, Huang S, Xue P, et al (2025) Generative artificial intelligence (GAI) usage guidelines for scholarly publishing: a cross-sectional study of medical journals. BMC Medicine. https://doi.org/10.1186/s12916-025-03899-1

79. Frutuoso J (2025) Building a Safe and Transparent Workflow for Large Language Model (LLM)-Assisted Clinical Trials and Prediction Models: A Technical Report. Cureus. https://doi.org/10.7759/cureus.92571

80. Le X (2024) Assessment Reform in Higher Education: An Ethical Approach to Harness the Power of Generative Artificial Intelligence. Education, research and perspectives. https://doi.org/10.70953/erpv51.2412007

81. Guémann M, Médrinal C (2025) Ahead of time : generative AI policies for European Rehabilitation Journal. European Rehabilitation Journal. https://doi.org/10.52057/erj.v5i1.68

82. Aristombayeva M, Satybaldiyeva R, Maung BM, Lee D (2025) Guiding the uncharted: the emerging (and missing) policies on Generative AI in higher education. Frontiers in Education. https://doi.org/10.3389/feduc.2025.1644081

83. Ghnemat R, Saleh A (2026) Large language models for clinical artificial intelligence in healthcare a systematic review. Discover Artificial Intelligence. https://doi.org/10.1007/s44163-025-00784-x

84. Krasnoselskyi M, Artamonova N, Sukhina O, et al (2025) Plagiarism in the system of academic integrity in medical research (part 2). Український радіологічний та онкологічний журнал. https://doi.org/10.46879/ukroj.1.2025.113-128

85. Zielinski C, Winker MA, Aggarwal R, et al (2023) Generative AI, and Scholarly Manuscripts: WAME Recommendations on Chatbots and Generative Artificial Intelligence in Relation to Scholarly Publications. Open Access Macedonian Journal of Medical Sciences. https://doi.org/10.3889/oamjms.2023.11723

86. Khoruzhaya A, Varyukhina MD, Erizhokov RA, et al (2026) MEDAI-LLM-SUMM: a reporting checklist for medical text summarization studies using large language models. Frontiers in Digital Health. https://doi.org/10.3389/fdgth.2026.1761601

87. Khoruzha L (2025) Scientific Ethics and Integrity in the Context of Artificial Intelligence. Pedagogical education theory and practice Psychology Pedagogy. https://doi.org/10.28925/2311-2409.2025.431

88. BaHammam A (2025) The Transparency Paradox: Why Researchers Avoid Disclosing AI Assistance in Scientific Writing. Nature and Science of Sleep. https://doi.org/10.2147/NSS.S568375

89. Wright CY, Lartey M, Khomsi K, et al (2024) The African Journal Partnership Program's guidance on the use of AI in scholarly publishing. Ghana Medical Journal. https://doi.org/10.4314/gmj.v58i1.1

90. (2025) I Used AI in the Drafting of this Editorial. How Should I Reference AI's Contribution? American Journal of Health Promotion. https://doi.org/10.1177/08901171251324731

91. Medicine TETF in P (2025) Artificial Intelligence in Academic Publishing: Expectations for Authors and Reviewers in Frontier Preventive Medicine. Frontiers in Preventive Medicine. https://doi.org/10.64904/fpm25020

92. Park S, Suh CH, Lee JH, et al (2025) Minimum Reporting Items for Clear Evaluation of Accuracy Reports of Large Language Models in Healthcare (MI-CLEAR-LLM): 2025 Updates. Korean Journal of Radiology. https://doi.org/10.3348/kjr.2025.1522

93. Raitskaya L, Tikhonova E (2023) Academic Integrity: Author-Related and Journal-Related Issues. Journal of Language and Education. https://doi.org/10.17323/jle.2023.18489

94. An Y, Yu JH, James S (2025) Investigating the higher education institutions' guidelines and policies regarding the use of generative AI in teaching, learning, research, and administration. International Journal of Educational Technology in Higher Education. https://doi.org/10.1186/s41239-025-00507-3

95. Huo B, Collins GS, Chartash D, et al (2025) Reporting guideline for Chatbot Health Advice studies: the CHART statement. BMC Medicine. https://doi.org/10.1186/s12916-025-04274-w

96. Cornelissen J, Höllerer MA, Boxenbaum E, et al (2024) Large Language Models and the Future of Organization Theory. Organization Theory. https://doi.org/10.1177/26317877241239056

97. Noh GY (2025) Generative artificial intelligence and editorial ethics: a roadmap for Health & New Media Research. Health & New Media Research. https://doi.org/10.22720/hnmr.2025.00304

98. O'Brien C, Thayani Z, Smith T, et al (2025) Evaluating AI guidelines in leading family medicine journals: a cross-sectional study. BMC Primary Care. https://doi.org/10.1186/s12875-025-03044-0

99. Peterson C, Deschênes M-C (2025) AI Governance in Academia: Guidelines for Generative AI. The Florida AI Research Society. https://doi.org/10.32473/flairs.38.1.138855

100. Weaver KD (2024) The Artificial Intelligence Disclosure (AID) Framework: An Introduction. College & Research Libraries News. https://doi.org/10.48550/arXiv.2408.01904

101. Zahn-Muñoz C, Viancos-González P, Alarcón-Henríquez N, et al (2026) Policies and Guidelines for the Use of Artificial Intelligence in Latin American Journals Indexed in Scopus and Classified According to the Scimago Journal Rank (SJR). Publications. https://doi.org/10.3390/publications14010017

102. Penabad-Camacho L, Morera-Castro M, Penabad-Camacho MA (2024) Guía para uso y reporte de inteligencia artificial en revistas científico-académicas. Revista Electrónica Educare. https://doi.org/10.15359/ree.28-s.19830

103. Farotimi O, Dunn A, Lissa CV van, et al (2025) Guidance for manuscript submissions testing the use of generative AI for systematic review and meta-analysis. Research Synthesis Methods. https://doi.org/10.1017/rsm.2025.10058

104. Kaebnick G, Magnus D, Kao A, et al (2023) Editors' statement on the responsible use of generative AI technologies in scholarly journal publishing. Medicine, Health care and Philosophy. https://doi.org/10.1007/s11019-023-10176-6

105. Young J, Young JL (2025) AI in Manuscript Development, Review, and Publication: Ethical Use, Accountability, and Justice-Centered Scholarship. Journal of Urban Mathematics Education. https://doi.org/10.21423/jume-v18i2a851

106. Kaebnick G, Magnus D, Kao A, et al (2023) Editors' Statement on the Responsible Use of Generative AI Technologies in Scholarly Journal Publishing. The Hastings center report. https://doi.org/10.1002/hast.1507

107. Huo B (2025) Reporting Guideline for Chatbot Health Advice Studies: Chatbot Assessment Reporting Tool (CHART) Statement. Annals of Family Medicine. https://doi.org/10.1370/afm.250386

108. Machin-Mastromatteo J-D (2026) Recomendaciones para elaborar políticas editoriales sobre el uso de inteligencia artificial generativa en la publicación científica. Revista Estudios de la Información. https://doi.org/10.54167/rei.v3i2.2193

109. Bommasani R, Klyman K, Longpre S, et al (2023) The Foundation Model Transparency Index. arXivorg. https://doi.org/10.48550/arXiv.2310.12941

110. Smith G, Bello C, Bialic-Murphy L, et al (2024) Ten simple rules for using large language models in science, version 1.0. PLoS Comput Biol. https://doi.org/10.1371/journal.pcbi.1011767

111. Parker JL, Richard VM, Becker K (2023) Guidelines for the Integration of Large Language Models in Developing and Refining Interview Protocols. The Qualitative Report. https://doi.org/10.46743/2160-3715/2023.6801

112. Abbasi K (2023) Human, all too human – why artificial intelligence cannot "author" papers. Journal of the Royal Society of Medicine. https://doi.org/10.1177/01410768231179306

113. Thelwall Mr (2026) Large language models and responsible research evaluation: an extension of the Leiden Manifesto. Scientometrics. https://doi.org/10.1007/s11192-026-05552-x

114. Hufton A (2023) No artificial intelligence authors, for now. Patterns. https://doi.org/10.1016/j.patter.2023.100731

115. Blau W, Cerf VG, Enriquez J, et al (2024) Protecting scientific integrity in an age of generative AI. Proceedings of the National Academy of Sciences of the United States of America. https://doi.org/10.1073/pnas.2407886121

116. Marušić A (2023) JoGH policy on the use of artificial intelligence in scholarly manuscripts. Journal of Global Health. https://doi.org/10.7189/jogh.13.01002

117. Madeyski L, Kitchenham B, Shepperd MJ (2025) LLM4SCREENLIT: Recommendations on Assessing the Performance of Large Language Models for Screening Literature in Systematic Reviews. arXivorg. https://doi.org/10.48550/arXiv.2511.12635

118. Lingard L, Driessen E, Oswald K (2025) The blurred threshold of AI-use disclosure: International journal editor expectations of sufficiency and necessity. medRxiv. https://doi.org/10.1101/2025.07.17.25331725

119. Miao B, Chen IY, Williams CYK, et al (2024) Updating the Minimum Information about CLinical Artificial Intelligence (MI-CLAIM) checklist for generative modeling research. arXivorg. https://doi.org/10.48550/arXiv.2403.02558

120. Knöchel T-D, Schweizer KJ, Acar O, et al (2025) Core principles of responsible generative AI usage in research. AI and Ethics. https://doi.org/10.1007/s43681-025-00768-8

121. Nery C (2025) All about people! Journal of the Foot & Ankle. https://doi.org/10.30795/jfootankle.2025.v19.1901

122. Wahle JP, Ruas T, Mohammad SM, et al (2023) AI Usage Cards: Responsibly Reporting AI-Generated Content. ACM/IEEE Joint Conference on Digital Libraries. https://doi.org/10.1109/JCDL57899.2023.00060

123. Beshyah S, Aburawi E, Elkhammas E (2026) Responsible Use of Generative Artificial Intelligence in Scholarly Publishing: The IJMBS Expectations. Ibnosina Journal of Medicine and Biomedical Sciences. https://doi.org/10.1055/s-0046-1817823

124. Ibrahim H, Liu X, Denniston A (2021) Reporting guidelines for artificial intelligence in healthcare research. Clinical and Experimental Ophthalmology. https://doi.org/10.1111/ceo.13943

125. Anthuvan T, Prabhuram DrS, Raju G, et al (2025) BRING YOUR OWN AI (BYOAI) IN THE WORKPLACE: PATTERNS, PITFALLS, AND THE BYOAI-GOV™ FRAMEWORK FOR BEHAVIOR-AWARE GOVERNANCE. Lex localis - Journal of Local Self-Government. https://doi.org/10.52152/mmrgh422

126. Park S, Suh CH, Lee JH, et al (2024) Minimum Reporting Items for Clear Evaluation of Accuracy Reports of Large Language Models in Healthcare (MI-CLEAR-LLM). Korean Journal of Radiology. https://doi.org/10.3348/kjr.2024.0843

127. Park S, Suh CH (2024) Reporting Guidelines for Artificial Intelligence Studies in Healthcare (for Both Conventional and Large Language Models): What's New in 2024. Korean Journal of Radiology. https://doi.org/10.3348/kjr.2024.0598

128. Vasconcellos V (2023) Editorial – Inteligência artificial e coautoria de trabalhos científicos: discussões sobre utilização de ChatGPT em pesquisa e redação científicas. Revista Brasileira de Direito Processual Penal. https://doi.org/10.22197/rbdpp.v9i3.913

129. Reeping D, Hampton C, Özkan D (2025) Interrogating the Use of Large Language Models in Qualitative Research Using the Qualifying Qualitative Research Quality Framework. Studies in Engineering Education. https://doi.org/10.21061/see.174

130. Akgün M (2025) Author or prompter? Scientific writing, identity, and the Theseus paradox. Philosophy, Ethics, and Humanities in Medicine. https://doi.org/10.1186/s13010-025-00195-x

131. Gallifant J, Afshar M, Ameen S, et al (2024) The TRIPOD-LLM Statement: A Targeted Guideline For Reporting Large Language Models Use. medRxiv. https://doi.org/10.1101/2024.07.24.24310930

132. Spennemann D (2023) Children of AI: A Protocol for Managing the Born-Digital Ephemera Spawned by Generative AI Language Models. Publ. https://doi.org/10.3390/publications11030045

133. Carlson NA, Burbano VC (2025) The use of LLMs to annotate data in management research: Foundational guidelines and warnings. Strategic Management Journal. https://doi.org/10.1002/smj.70023

134. (2025) Transparencia y responsabilidad en el uso de inteligencia artificial en la investigación. Actualización continua de la política editorial de la Relime. Revista Latinoamericana de Investigación en Matemática Educativa. https://doi.org/10.12802/relime.24.2720

135. Molina JCC (2024) El uso de la IA como herramienta para la investigación académica: políticas editoriales y condicionantes éticos en el camino hacia su normalización. Derecom Derecho de la Comunicación. https://doi.org/10.5209/dere.98112

136. Selvi B, Eser RSA (2025) Digital Tools for Academic Writing: A Systematic Analysis of AI and Technology-Enhanced Support. Türkiye eğitim dergisi. https://doi.org/10.54979/turkegitimdergisi.1828027

137. Cardoso JG, Gammarano I (2025) Guia para o uso ético e responsável da inteligência artificial generativa no âmbito acadêmico. Revista de Ciências da Administração. https://doi.org/10.5007/2175-8077.2025.e109536

138. Hollister C, Schweikhard A, Hosier A, Williams J (2024) CIL's New Generative AI Policy. Communications in Information Literacy. https://doi.org/10.15760/comminfolit.2024.18.1.1

139. Gasparini L, Phillipson N, Capurro D, et al (2024) A survey of large language model use in a hospital, research, and teaching campus. Data & Policy. https://doi.org/10.1017/dap.2025.10044

140. Boutera A (2025) CHALLENGES OF USING ARTIFICIAL INTELLIGENCE TOOLS IN SCIENTIFIC WRITING. Metafizika Journal. https://doi.org/10.33864/2617-751x.2025.v8.i7.259-272

141. Omorogiuwa TBE, Mugumbate R, Harms-Smith L, et al (2025) Ethical and transparent use of generative Artificial Intelligence (AI): Ethics letter three (3) from the African Independent Ethics Committee (AIEC). African Journal of Social Work. https://doi.org/10.4314/ajsw.v15i1.11

142. (2024) Chatbots, IA generativa e manuscritos acadêmicos: recomendações da WAME sobre chatbots e inteligência artificial generativa em relação a publicações acadêmicas. BRASPEN journal. https://doi.org/10.37111/braspenj.2024.39.1.18

143. Sangwa DrS, Mutabazi P (2025) Generative AI and Academic Integrity in Online and Distance Learning: A Policy Index and Evidence for Assessment Redesign in the Global South. Open Journal of AI, Ethics & Society (ISSN: 3105-3076). https://doi.org/10.65655/dg1rsd17

144. Vondrovský V (2024) Editorial. Archeologické rozhledy. https://doi.org/10.35686/ar.2023.16

145. Fleurence R, Dawoud D, Bian J, et al (2024) ELEVATE-GenAI: Reporting Guidelines for the Use of Large Language Models in Health Economics and Outcomes Research: an ISPOR Working Group on Generative AI Report. Value in Health. https://doi.org/10.1016/j.jval.2025.06.018

146. Kaebnick G, Magnus D, Kao A, et al (2023) Editors' Statement on the Responsible Use of Generative AI Technologies in Scholarly Journal Publishing. Ethics & Human Research. https://doi.org/10.1002/eahr.500182

147. Kim J, Shin H-W (2026) Stage-Aware Governance of Large Language Models: Managing Uncertainty and Human Oversight in AI-Assisted Literature Review Systems. Systems. https://doi.org/10.3390/systems14020153

148. Perkins M, Roe J (2023) Academic publisher guidelines on AI usage: A ChatGPT supported thematic analysis. F1000Research. https://doi.org/10.12688/f1000research.142411.2

149. Solomon DH, Allen K, Katz P, et al (2023) ChatGPT, et al … Artificial Intelligence, Authorship, and Medical Publishing. ACR Open Rheumatology. https://doi.org/10.1002/acr2.11538

150. Aghemo A, Forner A, Valenti L (2023) Should Artificial Intelligence-based language models be allowed in developing scientific manuscripts? A debate between ChatGPT and the editors of Liver International. Liver international (Print). https://doi.org/10.1111/liv.15580

151. Burger B, Kanbach DK, Kraus S, et al (2023) On the use of AI-based tools like ChatGPT to support management research. European Journal of Innovation Management. https://doi.org/10.1108/ejim-02-2023-0156

152. Nath KA, Conway M, Fonseca R (2024) AI in Peer Review: Publishing's Panacea or a Pandora's Box of Problems? Mayo Clinic proceedings. https://doi.org/10.1016/j.mayocp.2023.11.013

153. Jeon K-H (2026) Limitations and mitigation strategies for using generative artificial intelligence in medical writing: a narrative review. Journal of Korean Medical Association. https://doi.org/10.5124/jkma.25.0163

154. Mangold S, Ream MA (2024) Artificial Intelligence in Graduate Medical Education Applications. Journal of Graduate Medical Education. https://doi.org/10.4300/jgme-d-23-00510.1

155. Hair JF, Sabol MA (2025) Artificial Intelligence (AI) in Competitive Intelligence (CI) Research. SDGs Studies Review. https://doi.org/10.37497/sdgs.v6istudies.43

156. Murphy C, Thomas FP (2023) Navigating the AI revolution: This journal's journey continues. Journal of Spinal Cord Medicine (JSCM). https://doi.org/10.1080/10790268.2023.2221593

157. Sodangi U, Isma'il A (2025) Responsible integration of generative artificial intelligence in academic writing: a narrative review and synthesis. Journal of Artificial Intelligence, Machine Learning and Neural Network. https://doi.org/10.55529/jaimlnn.52.13.23

158. Crawford J, Cowling M, Ashton-Hay S, et al (2023) Journal of University Teaching & Learning Practice Journal of University Teaching & Learning Practice. Journal of University Teaching and Learning Practice

159. Alduais A, Qadhi S, Chaaban Y, Khraisheh M (2025) The ETHICAL Protocol for Responsible Use of Generative AI for Research Purposes in Higher Education. The AI Magazine. https://doi.org/10.1002/aaai.70047

160. Hair J, Sabol MA (2024) Leveraging Artificial Intelligence (AI) in Competitive Intelligence (CI) Research. Revista Inteligência Competitiva. https://doi.org/10.24883/eaglesustainable.v15i.469

161. Alba C, Xi W, Wang C, An R (2025) ChatGPT Comes to Campus: Unveiling Core Themes in AI Policies Across U.S. Universities with Large Language Models. Technical Symposium on Computer Science Education. https://doi.org/10.1145/3641555.3705141

162. Gurnal P, Rana L (2025) Artificial Intelligence and Publishing Ethics: A Narrative Review and SWOT Analysis. Cureus. https://doi.org/10.7759/cureus.84098

163. Cheng A, Calhoun A, Reedy G (2025) Artificial intelligence-assisted academic writing: recommendations for ethical use. Advances in Simulation. https://doi.org/10.1186/s41077-025-00350-6

164. Moreno A, Bitterman D (2024) Toward Clinical-Grade Evaluation of Large Language Models. International Journal of Radiation Oncology, Biology, Physics. https://doi.org/10.1016/j.ijrobp.2023.11.012

165. Sithole STM, Ashraf SF (2025) Generative AI in Accounting Publishing: A Bibliometric and Thematic Analysis of Author Guidelines with Governance and Ethical Considerations. European Scientific Journal. https://doi.org/10.19044/esj.2025.v21n31p1

166. Huo B (2024) Protocol for the development of the Chatbot Assessment Reporting Tool (CHART) for clinical advice. BMJ Open. https://doi.org/10.1136/bmjopen-2023-081155

167. Ntsobi P (2024) Challenges, Benefits and Recommendations for Using Generative Artificial Intelligence in Academic Writing - A Case of ChatGPT. Medicon Engineering Themes. https://doi.org/10.55162/mcet.07.236

168. Dai Y, Lai S, Lim C, Liu A (2024) University policies on generative AI in Asia: promising practices, gaps, and future directions. Journal of Asian Public Policy. https://doi.org/10.1080/17516234.2024.2379070

169. Almufarreh A, Ahmad A, Arshad M, et al (2025) Ethical implications of ChatGPT and other large language models in academia. Frontiers Artif Intell. https://doi.org/10.3389/frai.2025.1615761

170. Hanafi AM, Ahmed M, Al-mansi MM, Al-Sharif OmarA (2025) Generative AI in Academia: A Comprehensive Review of Applications and Implications for the Research Process. International Journal of Engineering and Applied Sciences-October 6 University. https://doi.org/10.21608/ijeasou.2025.349520.1041

171. Chaka C, Shange T, Nkhobo T, Hlatshwayo V (2024) An Environmental Review of the Generative Artificial Intelligence Policies and Guidelines of South African Higher Education Institutions: A Content Analysis. International Journal of Learning, Teaching and Educational Research. https://doi.org/10.26803/ijlter.23.12.25

172. Pesapane F, Nicosia L, Cassano E (2025) The Instrument Is Not the Claim: LLMs and Responsibility in Radiology. European Journal of Radiology Artificial Intelligence. https://doi.org/10.1016/j.ejrai.2025.100063

173. Seghers B, Tůma O (2024) Artificial intelligence transforms research, but the integrity norms do not change. Developmental Medicine & Child Neurology. https://doi.org/10.1111/dmcn.15912

174. Goto A, Katanoda K (2023) Should We Acknowledge ChatGPT as an Author? Journal of Epidemiology. https://doi.org/10.2188/jea.JE20230078

175. Machin-Mastromatteo J-D (2023) Implicaciones y políticas editoriales de la inteligencia artificial. Revista Estudios de la Información. https://doi.org/10.54167/rei.v1i2.1448

176. Fleurence R, Bian J, Wang X, et al (2024) Generative AI for Health Technology Assessment: Opportunities, Challenges, and Policy Considerations. arXivorg. https://doi.org/10.48550/arXiv.2407.11054

177. Cho WI, Cho E, Cho K (2023) PaperCard for Reporting Machine Assistance in Academic Writing. arXivorg. https://doi.org/10.48550/arXiv.2310.04824

178. Kaur D (2026) Impact of Large Language Models on Research Writing and Publication Practices. International Journal of Sciences and Innovation Engineering. https://doi.org/10.70849/ijsci03022699663

179. Najm M, Najm MM (2024) Balancing innovation with responsibility: A policy proposal for ethical artificial intelligence use in medical scholarly publication. Yemen Journal of Medicine. https://doi.org/10.32677/yjm.v3i1.4552

180. Luo X, Tham Y, Daher M, et al (2024) Protocol for developing the reporting guidelines for the use CHatbots and other gEnerative AI tools in mEdical Research (CHEER). medRxiv. https://doi.org/10.1101/2024.10.15.24315506

181. Goldberg A, Ullah I, Khuong TGH, et al (2024) Usefulness of LLMs as an Author Checklist Assistant for Scientific Papers: NeurIPS'24 Experiment. arXivorg. https://doi.org/10.48550/arXiv.2411.03417

182. Adams B, Dodge S, Purves R (2024) Research articles in the GPT era. Journal of Spatial Information Science. https://doi.org/10.5311/josis.2024.28.383

183. Huo B, Collins GS, Cacciamani GE, Guyatt GH (2025) Reporting guidelines for studies involving generative artificial intelligence applications: what do I use, and when? npj Digital Medicine. https://doi.org/10.1038/s41746-025-02113-z

184. Lin Z (2024) Beyond principlism: practical strategies for ethical AI use in research practices. AI and Ethics. https://doi.org/10.1007/s43681-024-00585-5

185. Ahn S (2024) Generative AI Guidelines in Korean Medical Journals: A Survey Using Human-AI Collaboration. medRxiv. https://doi.org/10.1101/2024.03.08.24303960

186. Reason T, Klijn S, Rawlinson W, et al (2025) Using Generative Artificial Intelligence in Health Economics and Outcomes Research: A Primer on Techniques and Breakthroughs. PharmacoEconomics - Open. https://doi.org/10.1007/s41669-025-00580-4

187. Bond A, Cilliers D, Retief F, et al (2024) Using an Artificial intelligence chatbot to critically review the scientific literature on the use of Artificial intelligence in Environmental Impact Assessment. Impact Assessment and Project Appraisal. https://doi.org/10.1080/14615517.2024.2320591

188. Chakravorti T, Wang X, Venkit P, et al (2025) Social Scientists on the Role of AI in Research. Proceedings of the AAAI/ACM Conference on AI, Ethics, and Society. https://doi.org/10.48550/arXiv.2506.11255

189. Phu ND, Thanh PM, Nghia TT (2025) GENERATIVE ARTIFICIAL INTELLIGENCE AND AUTHORSHIP GOVERNANCE IN VIETNAMESE SCIENTIFIC JOURNALS: LEGAL CHALLENGES AND POLICY OPTIONS. Veredas do Direito. https://doi.org/10.18623/rvd.v22.n7.3954

190. Ribeiro B (2023) O ChatGPT e as Publicações Científicas. revista de Medicina Desportiva Informa. https://doi.org/10.23911/chatgpt_2023_jul

191. Manyengo P (2024) Managing Research in Higher Learning Institutions (HLIs) in Tanzania : A Systematic Review on the best Practices for using Artificial Intelligence. JOURNAL OF ISSUES AND PRACTICE IN EDUCATION. https://doi.org/10.61538/jipe.v15i2.1392

192. Cooper N, Clark A, Lecomte N, et al (2024) Harnessing large language models for coding, teaching and inclusion to empower research in ecology and evolution. Methods in Ecology and Evolution. https://doi.org/10.1111/2041-210X.14325

193. Daoudi M (2025) Ethical limits and suggestions for improving the use of AI in scientific research, academic publishing, and the peer review process, based on deontological and consequentialist viewpoints. Discover Education. https://doi.org/10.1007/s44217-025-00696-z

194. Blythe PA, Kulis C, McGraw AP, et al (2025) Comments on "AI and the advent of the cyborg behavioral scientist". Journal of Consumer Psychology. https://doi.org/10.1002/jcpy.1453

195. Hamoda T, Wyns C, Pinggera G, et al (2025) Artificial Intelligence in Scientific Writing: Balancing Innovation and Efficiency with Integrity: Perspectives and Position Statements of Global Andrology Forum Expert Group. The World Journal of Men's Health. https://doi.org/10.5534/wjmh.240007

196. (2024) AI-powered literature search: some observations and concerns. Khyber Medical University Journal. https://doi.org/10.35845/kmuj.2024.23740

197. Breznau N, Nguyen HHV (2025) An Introduction to Generative Artificial Intelligence for Academics. F1000Research. https://doi.org/10.12688/f1000research.166513.1

198. Carleton NM, Thakkar S (2020) How to Approach and Interpret Studies on AI in Gastroenterology. Gastroenterology. https://doi.org/10.1053/j.gastro.2020.04.001

199. Zrubka Z, Kovács L, Nezhad HM, et al (2025) Artificial Intelligence in Medicine: A Systematic Review of Guidelines for the Reporting and Interpretation of Studies. Acta Polytechnica Hungarica. https://doi.org/10.12700/aph.22.10.2025.10.8

200. Allen JW, Schaefer OG, Mann SP, et al (2024) Augmenting research consent: should large language models (LLMs) be used for informed consent to clinical research? Research ethics. https://doi.org/10.1177/17470161241298726
