# Supplementary material for: Disclosure is not documentation: an open science framework for documenting generative AI use in scholarly research and publication workflows
Source: Res Integr Peer Rev. 2026 Aug 15;11:47. doi: 10.1186/s41073-026-00245-8 (PMC13483690; doi:10.1186/s41073-026-00245-8)
Supplement: Supplementary file 2 [file 41073_2026_245_MOESM1_ESM.docx]

AI-Use Documentation

for the manuscript "Disclosure is not documentation: An open science framework for documenting generative AI use in scholarly research and publication workflows"

Current manuscript version: V4.1 | First submitted manuscript version: V2.1 (30 April 2026) | Documentation updated: 7 August 2026 | Time zone: Europe/Berlin

*Supplementary material status: Additional file 1. This document provides the finalized project-specific record of AI-assisted workflows used during manuscript preparation and framework development.*

**Document card**

| **Field** | **Entry** |
| --- | --- |
| Manuscript title | Disclosure is not documentation: An open science framework for documenting generative AI use in scholarly research and publication workflows |
| Manuscript version | V4.1 |
| Author | Jan Christopher Cwik |
| Discipline / context | Scientific writing, Open Science, publication ethics, clinical psychology and psychotherapy as a sensitive example field |
| Documentation type | Project-specific AI-use documentation, prompt/use log, human-review record, and copy-ready AI-use declaration |
| Visibility status | Supplementary material (Additional file 1); author-reviewed and finalized for resubmission |
| Source basis for this documentation | Uploaded documents 01-08, the manuscript files, and author-provided usage information: ChatGPT Pro for language correction, argumentative stringency/coherence checks, and manuscript revision/consistency review, including documented prompt records; Grammarly for language correction, with version documented as Grammarly 1.163.1.0 and WebUI 2.14.4 and the language setting American English; Elicit for mapping under the Scale plan (academic). |

# 1. Purpose and scope

This document records the AI-assisted tools and workflows reported for the manuscript and accompanying documentation materials. It follows the logic of the uploaded documentation package: disclosure in the manuscript is complemented by process documentation covering tool, provider or access route, model/version information where available, date or period of use, purpose, input context, output use, human review, final relevance, and data-protection status.

The documentation is proportionate to the type of AI use. Grammarly use for spelling, grammar, and style is treated as low-relevance language support. ChatGPT Pro use for language correction and argumentative stringency checks is treated as low to medium relevance because it may affect wording and the organization or coherence of arguments. Elicit use for exploratory source and policy mapping is treated as extended documentation because it contributed to the evidence-mapping workflow that informed the framework.

Relevant target-journal and publisher AI-use requirements were reviewed and documented in Section 10. The manuscript, AI-use declaration, and supplementary AI-use documentation were cross-checked for consistency before resubmission.

# 2. Documentation standards applied

- Transparency: AI use is described by task, not merely by naming the tool.
- Human responsibility: all AI-generated or AI-assisted suggestions are subject to author review; AI tools are not authors.
- Proportionality: routine language correction is documented briefly, while evidence mapping receives extended documentation.
- Privacy-sensitive openness: no sensitive, personal, clinical, confidential peer-review, or third-party manuscript content should be publicly disclosed; redaction or summary-level documentation is used where needed.
- Traceability: the date, access route, purpose, input category, output use, human review, and final relevance are documented for the reported AI-assisted activities.

**Documentation level by use category**

| **Documentation level** | **Use in this project** | **Documentation consequence** |
| --- | --- | --- |
| Green - low relevance | Grammarly spelling, grammar, punctuation, and routine style support. | Tool and purpose are documented; exact prompts are not applicable. |
| Yellow - medium relevance | ChatGPT Pro language correction and argumentative stringency/coherence checks of author-generated manuscript text. | Tool, date, exact author-provided prompt without reproducing the manuscript content, input category, output use, author review, and final relevance are documented. |
| Red - high relevance / extended documentation | Elicit-supported exploratory evidence and policy mapping used to identify source types, policy domains, and possible reporting requirements. | Extended documentation records the guiding question, scope, output limits, N retained sources, manual verification, and non-systematic status. |
| Dark red - sensitive / restricted | Not reported for this project. | No personal clinical raw data, identifiable case material, confidential reviewer materials, sensitive participant data, or third-party unpublished manuscripts should be entered into public AI systems. |

# 3. Tool inventory

**AI-assisted tools reported for the project**

| **Tool** | **Provider / access route** | **Model or version status** | **Reported purpose** | **Date / period** |
| --- | --- | --- | --- | --- |
| ChatGPT Pro | OpenAI; ChatGPT Pro web interface as reported by author. | Current ChatGPT Pro web interface on 30 April 2026, as reported by the author. ChatGPT Pro / OpenAI web interface; tool, provider, access route, and date of use documented. | Language correction, readability improvement, argumentative stringency/coherence checking, peer-review-related revision support based on author-paraphrased revision instructions, manuscript-supplement consistency checking, and final copyediting. | 30 April 2026; 1 July 2026; 7 August 2026, Europe/Berlin. |
| Grammarly | Grammarly writing assistant; visible in the author-provided screenshot as Grammarly add-in/pane with American English selected. | Grammarly 1.163.1.0; WebUI 2.14.4; language setting: American English, according to the author-provided screenshot. | Spelling, grammar, punctuation, readability, and language correction of author-generated manuscript text. | Used during manuscript preparation; version/status documented by screenshot on 30 April 2026. |
| Elicit | Elicit; Scale plan (academic), as reported by the author. Manuscript notes that the user interface did not display a specific software or model version. | Specific software/model version not displayed in the interface according to the manuscript. | AI-assisted exploratory source and policy mapping for AI-use disclosure and documentation requirements. | 24 April 2026, Europe/Berlin, according to the manuscript methods section. |

# 4. Compact AI-use log

**Compact project-specific AI-use log**

| **ID** | **Purpose and context** | **Output use and human review** |
| --- | --- | --- |
| AIUSE-001 | ChatGPT Pro was used on 30 April 2026 for language correction, readability improvement, and argumentative stringency/coherence checking of author-generated manuscript text. No personal clinical raw data, identifiable case material, confidential reviewer content, sensitive participant data, or unpublished third-party manuscripts were reported as inputs. | Suggestions were reviewed by the author and adopted selectively. The author retained responsibility for final wording, argumentation, source interpretation, and scientific accuracy. The exact author-provided prompt for 30 April 2026 is documented in Section 5; the manuscript text that followed the prompt is not reproduced in the public prompt log. |
| AIUSE-002 | Grammarly was used for spelling, grammar, punctuation, style, and language correction of author-generated manuscript text. The author-provided screenshot documents Grammarly 1.163.1.0, WebUI 2.14.4, and American English as the selected language setting. The exact date/period of Grammarly use was not separately specified. | Automated suggestions were reviewed and accepted or rejected by the author. Use was limited to language-level support and did not contribute new scientific content, sources, analyses, or interpretations. |
| AIUSE-003 | Elicit was used on 24 April 2026 for an AI-assisted exploratory evidence and policy mapping under the Elicit Scale plan (academic), as reported by the author. The guiding question was: What information do current policies require or recommend that researchers disclose or document when using AI tools in scientific studies, manuscript preparation, data analysis, literature searches, peer review, or publication processes? | Elicit-supported outputs were used to identify source types, policy domains, and possible reporting requirements. The manuscript states that N = 253 sources were retained after Elicit-supported screening and that the final AI-generated synthesis report included the N = 200 sources with the highest Elicit screening scores due to tool processing limits. All substantive policy claims retained in the final manuscript were manually reviewed by the author and verified against the cited primary policy or guidance sources. |
| AIUSE-004 | ChatGPT Pro was used during preparation of manuscript V3.1 to support revision following the first round of peer review. The author paraphrased and dictated the substance of relevant reviewer comments as revision instructions; the original reviewer reports and verbatim reviewer wording were not entered. The review focused on argumentative coherence, redundancy reduction, scientific style, and cross-manuscript consistency. | Suggestions were reviewed individually and adopted selectively by the author. Reviewer comments, source interpretation, scientific claims, references, and final responses remained under author control; no AI-generated references or unverified substantive claims were adopted. |
| AIUSE-005 | ChatGPT Pro was used during preparation of manuscript V4.1 to support final revision. The author paraphrased and dictated the substance of relevant reviewer comments as revision instructions; the original reviewer reports and verbatim reviewer wording were not entered. The review included consistency checking between the manuscript and supplementary AI-use documentation, wording refinement, repetition reduction, clarification of human verification, source support in tables, and final copyediting. | All proposed changes were reviewed and approved by the author. Policy and source claims retained in the manuscript were manually verified against cited sources. Final decisions concerning content, wording, references, tables, supplementary materials, and reviewer responses were made by the author. |

# 5. Detailed record: ChatGPT Pro

**Detailed records for ChatGPT Pro use**

| **Field** | **Entry** |
| --- | --- |
| Prompt / use ID | AIUSE-001 |
| Tool / provider / access route | ChatGPT Pro / OpenAI / web interface. |
| Model / version | Current ChatGPT Pro web interface on 30 April 2026, as reported by the author. Specific visible model/version not provided. Visible tool name, provider, access route, date, and exact prompt were documented. |
| Date and time | 30 April 2026, Europe/Berlin. |
| Manuscript phase / section | Manuscript preparation and revision; the documented interaction on 30 April 2026 concerned author-generated manuscript text. |
| Purpose | Language correction; readability and style improvement; argumentative stringency and coherence check. |
| Input / context | Author-generated manuscript text or excerpts. For the documented 30 April 2026 use, the author-provided prompt was followed by the manuscript text. The manuscript text itself is not reproduced in this AI-use documentation. No personal clinical raw data, identifiable case material, confidential reviewer content, sensitive participant data, or unpublished third-party manuscripts were reported as inputs. |
| Data protection status | The input consisted of the author's own unpublished manuscript text. No personal clinical raw data, identifiable case material, confidential reviewer content, sensitive participant data, or unpublished third-party manuscripts were entered. |
| Exact prompt | **Original author-provided ChatGPT prompt from 30 April 2026 (German; the manuscript text that followed the final line is not reproduced in this public documentation):**  Bitte überprüfe das gesamte Manuskript sorgfältig hinsichtlich Redundanzen, Stringenz der Argumentation und eines natürlichen wissenschaftlichen Schreibstils. Achte insbesondere darauf, mechanisch wirkende Aufzählungen zu vermeiden und Übergänge zwischen Abschnitten, Argumenten und Befunden flüssiger zu gestalten.  Mache mir anschließend konkrete Änderungsvorschläge, ohne Sinn, Inhalt oder Argumentationslogik des Manuskripts zu verändern. Alle von mir eingefügten Referenzen und Zitationen müssen vollständig erhalten bleiben. Bitte achte zudem darauf, dass der jeweils zitierte Sinn der Quellen nicht verfälscht oder verschoben wird.  Überprüfe außerdem Rechtschreibung, Grammatik, Zeichensetzung und sprachliche Präzision. Formuliere Verbesserungsvorschläge so, dass der Text stilistisch klarer, stringenter und weniger redundant wird, aber weiterhin meinem wissenschaftlichen Ton entspricht.  Bitte strukturiere deine Rückmeldung wie folgt: Übergreifende Einschätzung zu Redundanzen, Stringenz und Stil Konkrete Änderungsvorschläge mit Angabe der betreffenden Textstellen Vorschläge für alternative Formulierungen, sofern sinnvoll Hinweise zu Rechtschreibung, Grammatik und sprachlicher Glättung Besondere Hinweise, falls Referenzen, Argumentationslogik oder zitierter Sinn gefährdet sein könnten  Wichtig: Bitte keine Referenzen entfernen, ersetzen oder inhaltlich umdeuten. Änderungen sollen ausschließlich der sprachlichen, strukturellen und argumentativen Verbesserung dienen.  Manuskript: [Author-generated manuscript text followed here; not reproduced in this AI-use documentation.]  **English translation of the original German prompt (provided for accessibility; this translation was not the prompt submitted to ChatGPT):**  *Please carefully review the entire manuscript for redundancies, the coherence and rigor of the argumentation, and a natural scientific writing style. In particular, please avoid mechanically structured lists and make the transitions between sections, arguments, and findings more fluent.*  *Then provide specific suggestions for revisions without changing the meaning, content, or argumentative logic of the manuscript. All references and citations that I have inserted must be retained in full. Please also ensure that the meaning conveyed by the cited sources is not distorted or shifted.*  *Please also check spelling, grammar, punctuation, and linguistic precision. Formulate suggestions for improvement so that the text becomes stylistically clearer, more coherent, and less redundant while continuing to reflect my scientific writing style.*  *Please structure your feedback as follows:* *Overall assessment of redundancies, argumentative coherence and rigor, and style* *Specific suggestions for revisions, indicating the relevant passages* *Suggestions for alternative wording, where appropriate* *Comments on spelling, grammar, and linguistic refinement* *Specific warnings if references, argumentative logic, or the intended meaning of cited sources might be compromised*  *Important: Please do not remove, replace, or reinterpret any references. Changes should be made exclusively to improve the language, structure, and argumentation.*  [The author-generated manuscript text was provided after this prompt but is not reproduced here to avoid unnecessary duplication of manuscript content.] |
| Relevant output | Language edits, readability suggestions, notes on argumentative stringency/coherence, section-specific change suggestions, alternative formulations where useful, and warnings where references, argumentative logic, or cited meaning might be at risk. No AI-generated references or new empirical claims should be adopted without independent verification. |
| Output use | Partly used / selectively adopted. Suggestions were evaluated by the author and incorporated only where appropriate. |
| Human review | Author reviewed all suggestions, checked consistency with the manuscript argument, verified source-related claims, revised final wording, and approved the final text. |
| Final relevance | Primarily language quality and coherence; possible influence on presentation and flow of the argument, but not on data analysis, empirical results, or independent source verification. |
| Documentation status | The exact author-provided prompt, date of use, tool/provider/access route, purpose, input category, output handling, human review, and final relevance are documented. |

**Detailed record for ChatGPT Pro use – manuscript V3.1**

| **Field** | **Entry** |
| --- | --- |
| Prompt / use ID | AIUSE-004 |
| Tool / provider / access route | ChatGPT Pro / OpenAI / web interface. |
| Date and time | 1 July 2026, Europe/Berlin. |
| Manuscript phase / section | Revision of manuscript V3.1 following the first round of peer review. |
| Purpose | Revision support and consistency review in response to peer-review feedback. |
| Input / context | Author-generated manuscript text and author-generated revision instructions derived from the peer-review process. The substance of reviewer comments was paraphrased and dictated by the author for revision purposes; the original reviewer reports and their verbatim wording were not entered into ChatGPT. No personal clinical raw data, identifiable case material, sensitive participant data, confidential third-party manuscripts, or unpublished third-party materials were entered. |
| Prompt documentation | Please review the revised manuscript and the reviewer comments systematically. Check whether each reviewer comment has been addressed adequately and whether the revisions are internally consistent across the manuscript. Pay particular attention to argumentative coherence, remaining redundancies, scientific style, consistency of terminology, and the correspondence between claims and cited sources. Do not remove, replace, invent, or reinterpret references. Identify any passages that still require clarification or revision and propose concrete wording changes where appropriate. The final scientific decisions, interpretation of sources, and responses to reviewers remain with the author. |
| Relevant output | Revision suggestions concerning reviewer-response coverage, argumentative coherence, redundancy, scientific style, terminology, and manuscript consistency. |
| Output use | Suggestions were evaluated by the author and adopted selectively where appropriate. |
| Human review | The author reviewed all suggestions, checked them against the reviewer comments and manuscript sources, retained responsibility for source interpretation and scientific claims, and approved the final revisions. |
| Final relevance | Support for revision and presentation of manuscript V3.1; no independent authorship, data analysis, or autonomous source selection. |
| Documentation status | Task, purpose, input category, prompt/instruction, output handling, human review, and manuscript relevance are documented. |

**Detailed record for ChatGPT Pro use – manuscript V4.1**

| **Field** | **Entry** |
| --- | --- |
| Prompt / use ID | AIUSE-005 |
| Tool / provider / access route | ChatGPT Pro / OpenAI / web interface. |
| Date and time | 7 August 2026, Europe/Berlin. |
| Manuscript phase / section | Final revision of manuscript V4.1 and supplementary AI-use documentation. |
| Purpose | Final consistency review, documentation alignment, wording refinement, repetition reduction, human-verification clarification, table/source-support review, and copyediting. |
| Input / context | Author-generated manuscript text and author-generated revision instructions derived from the peer-review process. The substance of reviewer comments was paraphrased and dictated by the author for revision purposes; the original reviewer reports and their verbatim wording were not entered into ChatGPT. No personal clinical raw data, identifiable case material, sensitive participant data, confidential third-party manuscripts, or unpublished third-party materials were entered. |
| Prompt documentation | Please review the current revised manuscript and the accompanying supplementary AI-use documentation for final resubmission. Check consistency between the manuscript, the AI-use declaration, and the supplementary documentation; identify remaining repetition, overstated claims, incomplete or inconsistent descriptions of human verification, and missing or insufficient source support in tables. Check language, grammar, punctuation, terminology, table formatting, and internal consistency. Do not invent, remove, replace, or reinterpret references or policy claims. Any substantive claim or citation-related suggestion must remain subject to manual verification by the author. Provide concrete revision suggestions while preserving the manuscript's meaning, argument, and scientific responsibility. |
| Relevant output | Suggestions concerning manuscript-supplement consistency, wording, repetition, verification documentation, table source support, copyediting, and final presentation. |
| Output use | Suggestions were evaluated by the author and adopted selectively where appropriate. |
| Human review | The author reviewed and approved all changes, manually verified retained policy and source claims against cited sources, and made all final decisions concerning content, wording, references, tables, supplementary materials, and reviewer responses. |
| Final relevance | Support for final revision, documentation alignment, and presentation of manuscript V4.1 and Additional file 1. |
| Documentation status | Task, purpose, input category, prompt/instruction, output handling, human review, and manuscript relevance are documented. |

# 6. Detailed record: Grammarly

**Detailed record for Grammarly use**

| **Field** | **Entry** |
| --- | --- |
| Prompt / use ID | AIUSE-002 |
| Tool / provider / access route | Grammarly writing assistant; visible in the author-provided screenshot as Grammarly add-in/pane with American English selected. |
| Model / version | Grammarly 1.163.1.0; WebUI 2.14.4; language setting: American English, according to the author-provided screenshot. |
| Date and time | During manuscript preparation; version and language setting documented by the author-provided screenshot dated 30 April 2026. |
| Manuscript phase / section | Language editing of author-generated manuscript text. |
| Purpose | Spelling, grammar, punctuation, style, readability, and language correction. |
| Input / context | Author-generated manuscript text or excerpts. No personal clinical raw data, identifiable case material, confidential reviewer content, sensitive participant data, or unpublished third-party manuscripts were reported as inputs. |
| Data protection status | Author-generated manuscript text was processed for language support. No personal clinical raw data, identifiable case material, confidential reviewer content, sensitive participant data, or unpublished third-party manuscripts were entered. |
| Exact prompt | Not applicable; Grammarly provides automated writing suggestions rather than a prompt-based interaction. |
| Relevant output | Automated spelling, grammar, punctuation, and style suggestions. |
| Output use | Partly used / selectively accepted or rejected by the author. |
| Human review | The author reviewed all suggestions and retained final responsibility for wording, meaning, and scientific accuracy. |
| Final relevance | Low-level language support; no reported contribution to study design, analysis, interpretation, sources, claims, figures, or conclusions. |
| Documentation status | Grammarly 1.163.1.0, WebUI 2.14.4, American English setting, purpose, input category, output use, and human review are documented. |

# 7. Detailed record: Elicit exploratory mapping

**Detailed record for Elicit-supported mapping**

| **Field** | **Entry** |
| --- | --- |
| Prompt / use ID | AIUSE-003 |
| Tool / provider / access route | Elicit; Scale plan (academic), as reported by the author. |
| Model / version | The manuscript states that the Elicit user interface did not display a specific software or model version. |
| Date and time | 24 April 2026, Europe/Berlin, according to the manuscript methods section. |
| Project phase / section | Source and policy mapping; AI-assisted exploratory evidence mapping in the Methods section. |
| Guiding question | What information do current policies require or recommend that researchers disclose or document when using AI tools in scientific studies, manuscript preparation, data analysis, literature searches, peer review, or publication processes? |
| Purpose | To identify relevant source types, policy domains, recurring expectations, restrictions, unresolved issues, and possible reporting requirements for AI-use disclosure and documentation. |
| Input / context | Exploratory mapping question and search/mapping context related to AI use in research, writing, peer review, publication processes, and policy guidance. No personal, clinical, or confidential data were reported as inputs. |
| Data protection status | No sensitive or personal data reported. Mapping concerned public or scholarly source/policy domains. |
| Relevant output | Candidate sources, policy domains, possible reporting requirements, and an AI-generated synthesis report. Manuscript states that N = 253 sources were retained after Elicit-supported screening; the final AI-generated synthesis report included N = 200 highest-scoring sources because of tool processing limitations. |
| Output use | Indirect and partial. Elicit-supported mapping informed the framework by identifying recurring issues such as disclosure expectations, authorship and accountability, methodological reporting, confidentiality, peer review, data-related uses, and restricted or prohibited applications. |
| Human review | The author manually reviewed and contextualized the Elicit-supported output, added selected recent articles, verified substantive policy claims against the cited primary policy or guidance sources, and treated the mapping as exploratory rather than systematic. |
| Final relevance | Substantive methodological support for framework development and source/policy mapping, but not an exhaustive systematic review. |
| Limitations | The mapping may have missed relevant institutional policies, journal-specific webpages, non-English sources, or recently updated publisher guidance. Researchers using the framework should check current policies of target journals, publishers, institutions, ethics boards, and funders. |
| Documentation status | Elicit Scale plan (academic), date of use, guiding question, screening counts, synthesis limit, output use, and human-verification procedure are documented. |

# 8. Human review, verification, and source checking

The author remains responsible for the accuracy, originality, integrity, interpretation, and final wording of the manuscript and accompanying materials. AI-assisted suggestions were not treated as authoritative sources. Language suggestions were reviewed and revised by the author. Elicit-supported mapping outputs were treated as exploratory inputs to be checked against primary sources and current policy documents.

**Human review and verification log**

| **Review domain** | **Review step documented for this project** | **Status** |
| --- | --- | --- |
| Language and readability | ChatGPT Pro and Grammarly suggestions were reviewed and selectively adopted by the author. | Completed for the final supplementary record. |
| Argumentative stringency | ChatGPT Pro was used to identify coherence or stringency issues; final decisions about structure and argument were made by the author. | Completed for the final supplementary record. |
| Sources and policy claims | Elicit-supported output was used exploratorily; substantive policy claims retained in the final manuscript were manually reviewed and verified against the cited primary policy or guidance sources. | Completed for the final manuscript and supplementary record. |
| References and citations | No source was retained solely because it appeared in AI output. References and citations retained in the final manuscript were manually checked against the original source or a reliable bibliographic record. | Completed for the final manuscript. |
| Clinical / sensitive content | No personal clinical raw data, identifiable case material, sensitive participant data, original reviewer reports or verbatim reviewer wording, or third-party unpublished manuscripts were entered. For revision support, reviewer comments were represented only through author-paraphrased and dictated revision instructions. | Completed; safeguards documented. |
| Methods and mapping claims | The Elicit mapping is described as exploratory, not systematic, and its limitations are stated. | Completed and documented in the manuscript and this file. |

# 9. Data protection, confidentiality, and redaction record

**Data protection and redaction record**

| **Content type** | **Entered into AI tools?** | **Documentation / safeguard** |
| --- | --- | --- |
| Author-generated manuscript text | Yes, for ChatGPT Pro and Grammarly language/stringency support as reported. | Documented as author-generated manuscript text. For revision support, the author paraphrased and dictated the substance of relevant reviewer comments; original reviewer reports and verbatim reviewer wording were not entered. No sensitive personal, clinical, participant-related, or unpublished third-party material was entered. |
| Exploratory mapping question and literature/policy search context | Yes, for Elicit mapping as reported. | No personal data. Record date, tool, question, output use, and manual verification. |
| Personal raw data or identifiable participant data | No reported use. | Do not enter into public AI tools; no public prompt-log disclosure. |
| Clinical case material, therapy/session notes, diagnostic details, crisis information | No reported use. | Do not enter into public AI tools. Use only synthetic or strongly redacted examples if needed. |
| Confidential reviewer comments or peer-review materials | No reported use. | Do not enter into public AI tools unless explicitly permitted and protected; document internally only if relevant. |
| Unpublished third-party manuscripts or collaboration documents not approved for release | No reported use. | Do not enter into public AI tools; protect confidentiality and copyright. |
| Copyrighted long-form source texts or protected manuals/tests | No reported use. | Avoid uploading unless rights and tool terms permit it; do not publish protected content in prompt logs. |

# 10. Target-journal and publisher policy record

This section records the target-journal and publisher AI-use requirements consulted for the manuscript and supplementary documentation.

**Target-journal and publisher policy record**

| **Policy item** | **Project note** | **Status** |
| --- | --- | --- |
| Target journal / publisher | Research Integrity and Peer Review / BMC / Springer Nature. Target-journal submission instructions and BMC/Springer Nature AI-related editorial policies checked on 24 April 2026 | [x] checked |
| AI-use declaration required? | BMC/Springer Nature policy states that LLM use should be properly documented in the Methods section or a suitable alternative part of the manuscript; Springer Nature guidance also states that AI use should be declared transparently, while AI-assisted copy editing alone need not be declared. The manuscript therefore includes both a Methods description and an AI use declaration, with detailed supplementary AI-use documentation. | [x] checked |
| AI-assisted language editing / grammar tools | ChatGPT Pro and Grammarly were used for language correction, readability improvement, grammar, spelling, punctuation, style, and argumentative stringency/coherence checks. Pure AI-assisted copy editing for readability, grammar, spelling, punctuation, and tone does not need to be declared under BMC/Springer Nature policy, provided human accountability is retained. Because ChatGPT Pro was also used for argumentative stringency/coherence checks and because the manuscript concerns AI-use documentation, this use is nevertheless disclosed voluntarily and documented in the AI-use declaration and supplement. | [x] checked |
| AI-assisted evidence mapping / literature support | Elicit-supported exploratory source and policy mapping contributed to framework development and is therefore documented in the Methods section, AI-use declaration, and supplementary AI-use documentation. It is reported as exploratory and supporting evidence, not as a systematic review. Data extraction was conducted for 253 retained sources, while the final AI-generated synthesis report included the 200 highest-scoring sources because of tool-processing limitations. This is consistent with the supplement’s documentation of Elicit as extended documentation rather than routine language support. | [x] checked |
| Required details | Reported details include tool/provider/access route, model or version information where available, date or period of use, purpose, input category, output use, human review, source verification, responsibility statement. ChatGPT Pro: OpenAI web interface, documented uses on 30 April, 1 July, and 7 August 2026. Grammarly: Grammarly 1.163.1.0, WebUI 2.14.4, American English, documented by the author-provided screenshot. Elicit: Scale plan (academic), used on 24 April 2026. | [x] checked |
| Prohibited or restricted content | BMC/Springer Nature policy does not allow AI authorship and requires accountability by human authors. It also states that AI-assisted copy editing does not remove human responsibility. Springer Nature guidance says prompts should not include personal, sensitive, confidential, or copyrighted material; reviewers should not upload manuscripts into generative AI tools; and AI-supported peer-review evaluation must be declared by reviewers. For this project, no personal clinical raw data, identifiable case material, sensitive participant data, confidential peer-review material, or unpublished third-party manuscripts were entered into public AI systems. | [x] checked |
| Repository / supplement link | OSF repository / DOI: 10.17605/OSF.IO/A439J. The project-specific AI-use documentation is provided with the manuscript as Additional file 1. The manuscript text supplied after the documented ChatGPT prompt is not duplicated in the supplementary record. | [x] completed |

Target journal and publisher AI-use policies were checked on 24 April 2026. The manuscript AI-use declaration and supplementary AI-use documentation were reviewed for consistency with Research Integrity and Peer Review / BMC / Springer Nature requirements.

# 11. AI-use declaration

The following declaration documents the AI-assisted activities reported for the manuscript and accompanying materials.

**AI-use declaration**

Generative AI and AI-assisted writing tools were used supportively during the preparation of this manuscript and accompanying documentation materials. ChatGPT Pro (OpenAI, web interface; author-reported use of the current ChatGPT Pro web interface on 30 April 2026, Europe/Berlin; specific visible model/version not separately documented in the provided materials) was used for language correction, readability improvement, and argumentative stringency/coherence checks of author-generated manuscript text. The documented ChatGPT prompt asked for a review of redundancies, argumentative stringency, natural scientific writing style, transitions, spelling, grammar, punctuation, and linguistic precision, and explicitly instructed the tool not to remove, replace, or reinterpret references or to change the manuscript's meaning, content, or argumentative logic. Grammarly (Grammarly 1.163.1.0; WebUI 2.14.4; language setting: American English, documented in an author-provided screenshot; exact date/period of use not separately specified) was used for spelling, grammar, punctuation, style, readability, and language correction. Elicit (Scale plan, academic) was used on 24 April 2026 for an AI-assisted exploratory evidence and policy mapping to identify relevant source types, policy domains, and possible reporting requirements concerning AI-use disclosure and documentation. The Elicit-supported mapping was not a systematic review and did not replace manual source verification. No personal clinical raw data, identifiable case material, sensitive participant data, confidential peer-review material, or unpublished third-party manuscripts were entered into public AI systems. AI-generated or AI-assisted suggestions were reviewed, edited, verified, and approved by the author; suggestions were adopted only where they were consistent with the manuscript's argument and independently checked sources. The author takes full responsibility for the final content, interpretation, wording, and integrity of the manuscript and accompanying materials. ChatGPT Pro was additionally used during revision of manuscript V3.1 (1 July 2026) and V4.1 (7 August 2026) for peer-review-related revision support, manuscript-supplement consistency checking, review of human-verification descriptions and table source support, wording refinement, repetition reduction, and final copyediting. For these revision steps, the author paraphrased and dictated the substance of relevant reviewer comments as revision instructions; the original reviewer reports and verbatim reviewer wording were not entered into ChatGPT. The corresponding German prompts and English translations are documented in this supplementary record. All resulting suggestions were reviewed by the author and adopted selectively; final decisions regarding content, argumentation, sources, citations, tables, supplementary materials, reviewer responses, and wording remained with the author.

# 13. Submission and repository checklist

**Final review checklist**

| **Done** | **Checklist item** | **Evidence / note** |
| --- | --- | --- |
| [x] | AI tools used are documented with provider/access route and model/version information where available. | ChatGPT Pro, Grammarly, and Elicit listed in Section 3; ChatGPT prompt records for manuscript preparation and revisions are documented in Section 5; Grammarly version and Elicit plan are documented. |
| [x] | Purpose of each AI use is described. | Language correction; stringency/coherence check; exploratory mapping. |
| [x] | Input/context materials are described without exposing sensitive content. | Author-generated manuscript text; mapping question; no sensitive clinical or personal data reported. |
| [x] | Outputs and final use are documented. | Language suggestions selectively adopted; Elicit mapping used indirectly and manually verified. |
| [x] | Human review and author responsibility are explicit. | Sections 8 and 11. |
| [x] | No AI-generated sources, citations, or policy statements were adopted without verification. | Completed against the final reference list and source/policy record. |
| [x] | Data protection and confidentiality safeguards are documented. | Section 9. |
| [x] | Target-journal policy has been checked and recorded. | Completed; see Section 10. |
| [x] | AI-use declaration has been inserted into the appropriate manuscript or supplement location. | Completed; see Section 11. |
| [x] | The public version is redacted or summary-level where exact prompts, manuscript excerpts, or sensitive content should not be shared. | Exact prompt text is documented, but the manuscript text that followed the prompt is omitted from this supplementary documentation. |
| [x] | Version number and file name are clear. | Final file name recorded in Section 14. |

# 14. Versioning and file name

Final supplementary file name:

AI-Use_Documentation_Disclosure_is_not_documentation_FINAL_v4-1_2026-08-07.docx

**Version log**

| **Version** | **Date** | **Change** | **Responsible / review** | **Status** |
| --- | --- | --- | --- | --- |
| v0.1 | 2026-04-30 | Initial draft created from manuscript V1.0, uploaded documents 01-08, and author-provided AI-use information. | Author review required. | Draft |
| v0.2 | 2026-04-30 | Added Grammarly version information from author-provided screenshot (1.163.1.0, WebUI 2.14.4; American English setting) and Elicit subscription information (Scale plan, academic). Documentation record expanded with available tool/version information, prompt records, Elicit subscription information, and policy-check fields. | Author-provided update / AI documentation update. | Updated draft |
| v0.3 | 2026-04-30 | Added the exact author-provided ChatGPT Pro prompt from 30 April 2026, documented that the manuscript text following the prompt is not reproduced, and updated the AI-use declarations and confirmation checklist accordingly. | Author-provided update / AI documentation update. | Updated draft |
| v1.0 | 2026-04-30 | Initial project-specific AI-use documentation prepared for author review and subsequent manuscript submission. | Author review | Author approval \| Completed |
| Manuscript V1.1 | 2026-04-30 | Pre-submission manuscript stage documented during preparation of the initial AI-use record; original AI-use dates, tool information, and prompt records retained. | Author review / manuscript development | Pre-submission version |
| First submission – manuscript V2.1 | 2026-04-30 | AI-use documentation aligned with manuscript V2.1 for the first journal submission. The record documented the reported ChatGPT Pro, Grammarly, and Elicit activities used during manuscript preparation. | Author review | First submitted version |
| Revision – manuscript V3.1 | 2026-07-01 | AI-use documentation aligned with revised manuscript V3.1 following peer review while retaining the original AI-use dates, tool information, prompt records, and provenance information. | Author review / revision | Revised version submitted |
| Final supplementary documentation – manuscript V4.1 | 2026-08-07 | Finalized supplementary AI-use documentation aligned with manuscript V4.1. The prompt/use record was extended to document ChatGPT-assisted revision support for manuscript V3.1 and V4.1, alongside the previously documented AI-assisted activities. All relevant tool information, purposes, inputs, output handling, and human-verification procedures were checked for consistency with the manuscript; the policy-verification record and AI-use declaration were finalized; and the documentation was designated as Additional file 1. | Author review / finalization | Final supplementary version for resubmission |

# 15. Source basis: uploaded documents 01-08

**Uploaded materials used as the basis for this AI-use documentation**

| **Document** | **How it was used in this project-specific documentation** |
| --- | --- |
| 01 Documentation Manual | Applied principles of transparency, human responsibility, proportionality, prompt/use documentation, redaction, and manuscript/supplement/repository logic. |
| 02 Prompt-Log Templates | Used to structure the compact use log and detailed records for ChatGPT Pro, Grammarly, and Elicit. |
| 03 Checklists | Used to create the final review checklist, data-protection checks, and policy-check items. |
| 04 AI-Use Declarations | Used to create the short and extended copy-ready AI-use declaration. |
| 05 Example Documentations | Used to adapt wording for clinical psychology and psychotherapy contexts and to avoid disclosure of sensitive clinical content. |
| 06 Sources and Guidelines | Used as the source/policy anchor list and to emphasize target-journal policy tracking and source verification. |
| 07 Editing and Versioning | Used for version status, recommended file name, change log, public/redacted versioning, and release checks. |
| 08 Example Prompt | Used for quality criteria: documents should be standalone, traceable, template-based, privacy-sensitive, and manually verified. |

# 16. Source and policy anchors

The following anchors informed the source- and policy-orientation materials used for this project-specific documentation.

- Target-journal AI policy and submission instructions.
- COPE position on authorship and AI tools: AI tools are not authors; human authors remain accountable.
- ICMJE recommendations on AI use by authors, if the target journal follows medical or health-publishing standards.
- APA Journals and APA Style guidance, if submitting to a psychology journal or using APA reporting conventions.
- Publisher policy, for example Elsevier, Nature/Springer, Wiley, PLOS, Sage, Taylor & Francis, or the relevant target publisher.
- GDPR and institutional data-protection rules for any personal, clinical, or sensitive data context.
- OSF, TOP, and FAIR-oriented documentation principles for traceable, versioned, and reusable open materials.

# 17. Limitations of this AI-use documentation

- The documented German ChatGPT prompts and their English translations are included in this supplementary record. Author-generated manuscript text supplied with the prompts is not reproduced to avoid unnecessary duplication of manuscript content.
- The ChatGPT records identify ChatGPT Pro, OpenAI, the web interface, dates and purposes of use, prompt records, output handling, and human review. Grammarly is documented as version 1.163.1.0 with WebUI 2.14.4 and American English selected; Elicit is documented as the Scale plan (academic).
- The Elicit mapping is documented as exploratory and supporting, not as an exhaustive systematic review.
- The target journal and publisher AI-use requirements relevant to this submission were reviewed and documented in the policy record.
- The manuscript, AI-use declaration, and supplementary AI-use documentation were cross-checked for consistency before resubmission.
